# Supplementary material for: A phase I/IIa safety and efficacy trial of intratympanic gamma-secretase inhibitor as a regenerative drug treatment for sensorineural hearing loss
Source: Nat Commun. 2024 Mar 1;15:1896. doi: 10.1038/s41467-024-45784-0 (PMC10907343; doi:10.1038/s41467-024-45784-0)
Supplement: Supplementary file 1 — Supplementary Infomation [file 41467_2024_45784_MOESM1_ESM.pdf]

Supplementary Appendix

Safety and efficacy of intratympanic gamma-secretase inhibitor as a regenerative drug treatment for sensorineural hearing loss

Table of contents

List of Investigators and Authors 4

Data and Safety Monitoring Board (DSMB) 4

Table S1 Inclusion and exclusion criteria 5

Secondary endpoints 6

Secondary outcomes at 6 and 12 weeks 6

Table S2 Level of tinnitus as measured by the TFI 8

Secondary outcomes at month 6 and 12 8

Post-hoc exploratory analysis and mixed-effect model 11

Methods 11

Results Pure-Tone Audiometry at 6 and 12 months 12

Results Speech Perception in Noise at 6 and 12 months 12

Figure S1 Pure-tone audiograms of all patients that completed 12 weeks and 6 and 12 months follow up 13

Supplement on Distortion Product Otoacoustic Emissions 14

Section 1: Analysis based on original DP amplitude/SNR data 15

Table S3 (A) Statistically significant effects of the Repeated Measures ANCOVA, per DPOAE frequency (DP amplitudes at low-level primaries) 17

Table S3 (B) Statistically significant effects of the Repeated Measures ANCOVA, per DPOAE frequency (DP SNRs at low-level primaries) 18

Table S3 (D) Statistically significant effects of the Repeated Measures ANCOVA, per DPOAE frequency (DP SNRs at high-level primaries) 20

Figure S2 (A) Change from baseline: DPOAE Amplitudes/ Low-level primaries 21

Figure S2 (B) Change from baseline: DPOAE SNRs / Low-level primaries 22

Figure S2 (C) Change from baseline: DPOAE Amplitudes/ High-level primaries 23

Figure 2 (D) Change from baseline: DPOAE SNRs/ High-level primaries 24

Figure S3 "Clinically relevant" changes of the DP amplitudes and SNRs 25

Figure S4 (A) 'clinically relevant' DPOAE improvement 26

Figure S4 (B) 'clinically relevant' DPOAE deterioration 27

Figure S5 (A) Runs-test for pattern randomness of 'clinically relevant DPOAE improvements 28

Figure S5 (B) Runs-test for pattern randomness of 'clinically relevant DPOAE deterioration 29

Figure S6 (A) Bernoulli-test for pattern randomness of 'clinically relevant DPOAE improvement 30

Figure S6 (B) Bernoulli-test for pattern randomness of 'clinically relevant DPOAE deterioration 31

Section 2: Analysis based on DP amplitude data, after adaptation for SNR and signal averaging epochs 32

Table S4 (A) Statistically significant effects of the Repeated Measures ANCOVA, per DPOAE frequency (DP amplitudes at low-level primaries) 34

Figure S7 (A) Change from baseline: DPOAE Amplitudes/ Low-level primaries..............................36

Figure S7 (B) Change from baseline: DPOAE Amplitudes/ High-level primaries 37

Figure S8 “Clinically relevant” changes of the DP amplitudes and SNRs 38

Figure S9: (A) ‘clinically relevant’ DPOAE improvement, (B) ‘clinically relevant’ DPOAE deterioration 39

Figure S10: Runs-test for pattern randomness of (A) ‘clinically relevant DPOAE improvements, (B) ‘clinically relevant DPOAE deterioration..40

Figure S11 (A) Bernoulli-test for pattern randomness of ‘clinically relevant DPOAE improvement .41

Figure S11 (B) Bernoulli-test for pattern randomness of ‘clinically relevant DPOAE deterioration..42

Comparative assessment of findings of Section 1 and Section 2 43

Attachment 1: DPOAE amplitude estimates (adapted for SNR and signal averaging epochs)..44

Attachment 2: Bernoulli-based test for randomness of the distribution along frequency of clinically important outcomes..46

References 46

List of Investigators and Authors

Name	Institute	Land
Arram Elizabeth	University College London	United Kingdom
Bibas Athanasios	National & Kapodistrian University of Athens	Greece
Bilhet Asger	Nordic Bioscience	Denmark
Blackshaw Helen	University College London	United Kingdom
Cooper Hannah	University College London	United Kingdom
Dalhoff Ernst	University Hospital Tübingen	Germany
Edge Albert	Audion Therapeutics BV	the Netherlands
Hojgaard Karin	University College London	United Kingdom
Iliadou Eleftheria	National & Kapodistrian University of Athens	Greece
Khalil Sherif	University College London	UK
Kikidis Dimitris	National & Kapodistrian University of Athens	Greece
Lowenheim Hubert	University Hospital Tübingen	Germany
Markatos Nikos	National & Kapodistrian University of Athens	Greece
Middelink Leonie	Middelinc. Utrecht	the Netherlands
Mueller Marcus	University Hospital Tübingen	Germany
Pastiadis Kostas	National & Kapodistrian University of Athens	Greece
Saeed, Shakeel	University College London	United Kingdom
Rutten Rolf Jan	Audion Therapeutics BV, Amsterdam	the Netherlands
Schade-Mann Thore	University Hospital Tübingen	Germany
Schilder, Anne	University College London	United Kingdom
Schneider Fritz	University Hospital Tübingen	Germany
van Diggelen Femke	TTopstart BV	the Netherlands
van Es Helmuth	Audion Therapeutics BV	the Netherlands
Vardonikolaki Katerina	National & Kapodistrian University of Athens	Greece
Wilke August	Eli Lilly and company	UK
Wolpert, Stephan	University Hospital Tübingen	Germany
Yildirim Omursen	University College London	UK

Data and Safety Monitoring Board (DSMB)

Paul J. Govaerts	The Eargroup, Antwerp-Deurne
Lawrence R. Lustig	Columbia University College of Physicians and Surgeons New York
Hinrich Staecker	University of Kansas Medical Center, Otolaryngology, Head and Neck, Surgery Kansas City
Preben Homøe	Zealand University Hospital, Køge, Department of Otorhinolaryngology

Table S1 Inclusion and exclusion criteria

Inclusion criteria	Exclusion criteria
1. Male or female between 18 and 80 years of age<br>2. A primary complaint of hearing loss of ≤ 20 years in duration, the history suggesting this hearing loss to be of age-related, noise induced or idiopathic origin<br>3. A bilateral, symmetrical (<15 dBHL difference) SNHL (SNHL) with a pure tone average threshold across the frequencies 0.5, 1, 2, 4 and 8 kHz of between 25 and 60 dBHL with 2 or more frequencies less than 60 dBHL<br>4. Participants must have been offered the option of hearing aids prior to being approached to participate in this trial. Use of new hearing aids, defined as first-time use, or upgrades are not permitted from three months prior to trial entry until the last follow-up assessment<br>5. Able to understand and follow study personnel instructions, read and understand study documents and provide informed consent<br>6. Willing and able to attend all study visits<br>7. Willing and able to use adequate hearing protection and to refrain from engaging in exposure to loud noise if sufficient hearing protection is not possible<br>8. Willing to refrain from wearing the hearing aid in the treated ear from the first day of IMP administration until the last study visit<br>9. Willing and able to protect ear canal and middle ear from water exposure from the first day of IMP administration to one week after the last administration<br>10. Are not pregnant or breast feeding and do not plan to become pregnant or father a child during the study. Female participants (of childbearing potential) must be willing to use a highly effective method of contraception throughout the study and must be willing to submit to pregnancy test(s). Male participants must agree to ensure the use of a highly effective method of contraception method with their female partners throughout the study as described above, unless they have had a prior vasectomy.	1. Presenting with a primary complaint of tinnitus<br>2. A ‘true’ air-bone gap ≥15 dBHL in 3 or more contiguous frequencies between 0.5, 1, 2, 4 kHz<br>3. History of suspected or diagnosed genetic cause of hearing loss<br>4. Suspected or known diagnosis of inner ear pathology, congenital hearing loss, fluctuating hearing loss, Meniere disease or secondary endolymphatic hydrops, perilymph fistula, cochlear barotrauma, radiation-induced hearing loss, retro-cochlear lesion (including vestibular schwannoma)<br>5. Evidence of acute or chronic otitis media or otitis externa on examination; or a history of middle ear pathology and/or surgery (history of ventilation tubes allowed)<br>6. Any therapy known as ototoxic (e.g. aminoglycosides, cisplatin, loop diuretics, quinine, etc.) within 12 months of screening<br>7. Ongoing or planned systemic or local drug-based therapy for inner ear hearing loss or tinnitus during the study<br>8. Ongoing or planned anticoagulative medication. Aspirin and NSAID use is permitted prior to and during the study<br>9. Participants with a history of cancer in the past five years, except for adequately treated basal cell or squamous cell carcinoma of the skin, or in women having resected cervical atypia, or resected carcinoma in situ of the cervix<br>10. Participants considered by the Investigator to have an ear canal too narrow to allow for trans-tympanic drug application<br>11. Participants who are physically unable to complete the bi-thermal air caloric testing using VNG<br>12. Documented history of alcohol and drug abuse within 12 months of screening<br>13. Any clinically significant co-morbidity that would interfere with trial participation<br>14. Known hypersensitivity, allergy or intolerance to the study medication or vehicle or any history of severe abnormal drug reaction<br>15. Concurrent participation in another clinical trial or participation in another clinical trial within 30 days prior to study entry<br>16. Prior participation in this trial

Secondary endpoints

Secondary outcomes at 6 and 12 weeks (6 and 12 months are post hoc)

Pure tone audiometry and speech in noise audiometry, site specific

Germany:

Subgroup analyses per trial site showed that the 12 patients at the German site showed a mean estimated -4.00 dB improvement in the average pure-tone air-conduction hearing threshold across 2, 4, and 8 kHz in the treated ear at 6 weeks (95% CI -7.03 to -0.97; P=0.012) and -4.45 dB at 12 weeks (95% CI 7.86 to 1.23; P=0.010). Their mean SRT50n did not change from baseline to 6 weeks (estimated change 1.25 dB; 95% CI -0.65 to 3.16; P=0.186) and 12 weeks (estimated change -1.97 dB; 95% CI -4.18 to + 0.24; P= 0.077). Three patients (N=11; 27%) showed an SRT50n improvement of ≥2 dB at 6 weeks and 6 patients (N=11; 55%) at 12 weeks.

UK:

The 24 patients at the UK site showed no change in average pure-tone hearing threshold across 2, 4, and 8 kHz in the treated ear at 6 weeks (estimated change 0.28 dB; 95% CI -1.34 to 1.89; P=0.732) and at 12 weeks (estimated change 0.76 dB; 95% CI -0.60 to 2.13; P=0.269)) Their mean SRT50n worsened from baseline to 6 weeks (estimated change 1.59 dB; 95% CI 0.59 to 2.59; P=0.002) and 12 weeks (estimated change 1.46 dB; 95% CI 0.35 to 5.58; P= 0.011. One patient (N=24; 4%) showed an SRT50n improvement of ≥2 dB at 6 weeks and 2 patients (N=24; 8%) at 12 weeks.

Greece:

The 8 patients at the Greek site showed no change in average pure-tone hearing threshold across 2, 4, and 8 kHz in the treated ear at 6 weeks (estimated change 0.00 dB; 95% CI -4.88 to 4.88; P=1.00) and at 12 weeks (estimated change 1.97 dB; 95% CI -1.81 to 5.74; P=0.253). Their mean SRT50n did not change from baseline to 6 weeks (estimated change -1.59 dB; 95% CI -3.61 to 0.43 P=0.111) and at 12 weeks (estimated change -1.19 dB; 95% CI -4.34 to 1.96; P= 0.389). Two patients (N=8; 25%) showed an SRT50n improvement of ≥2 dB at 6 weeks and 2 patients (N=7; 29%) at 12 weeks.

Speech in noise audiometry: Change from baseline at 6 and 12 weeks
(difference between treated ear and untreated ear, site speciffic)

Germany:
Based on the treated – untreated data, 4 patients showed an improvement of at least -2 dB SNR at week 6 and 6 at week 12. Of these patients, 4 also showed an improvement as per the Pure Tone Audiometry Analysis.

UK:
Based on the treated minus untreated data, in 2 patients had an improvement of at least -2dB SNR at week 6 and 7 patients an improvement of at least -2 dB SNR at week 12. Of these, 4 patients also showed improvement as per the Pure Tone Audiometry Analysis.

Greece:
Based on the treated – untreated data, 2 patients showing an improvement of at least -2 dB SNR both at week 6 and 12. Of these patients, 1 patient also showed an improvement as per the Pure Tone Audiometry Analysis.

Middle ear immittance as tested by tympanometry and ART to determine middle ear pressure, volume and compliance values and acoustic threshold reflex shift
Overall there was no clinically significant difference observed on middle ear function and tympanometry between baseline and week 12. In addition, none of the patients had a persistent perforation due to the procedure.

DPOAE - SNR and absolute levels
*Week 12: There was no significant effect observed on the DPOAEs outcomes on a group level at week 6 and week 12. However, for some higher frequencies, differences are seen. For that reason, a post hoc analysis is conducted on a per patient level (also see **"Supplement on Distortion Product Otoacoustic Emissions")***

Cochlear dead regions as tested by the Threshold Equalising Noise test
There was no significant effect observed on the dead region outcomes on a group level at week 6 and week 12.

Level of tinnitus as measured by the TFI

The changes in TFI did not respond well with the tinnitus reported as Adverse Event, nor with the responses to the individual tinnitus assessment on an individual level. This is the reason for not reported these results.

Table S2 Level of tinnitus as measured by the TFI

	scr	w6	w12	m6	m12
mild	31	25	27	23	16
mod	8	9	2	6	3
sev	5	9	13	9	9
n	44	43	42	38	28

Balance: Change from baseline at 12 weeks, as measured by a clinical balance assessment – including History and Examination (Eye Movements, Head Thrust, modified Romberg, Unterberger, Bithermal Air Calorics using VNG), and Dizziness Handicap Inventory

The hearing and balance specific safety endpoints (DHI, TFI, Eye movements, Modified Romberg, Unterberger, Head Trust Test, VNG Air Calorics, Facial Nerve function, Taste Assessment, Tinnitus Assessment) showed no changes during study.

Secondary outcomes at month 6 and 12

Hearing Handicap Inventory

Overall, no significant changes in HHI scores were seen at a group level.

Hearing Aid Questionnaire

There are no statistically significant changes in scores from baseline to month 6 and month 12.

DPOAE - SNR and absolute levels

Month 12: For the mITT population, the outcomes of the DPOAEs in terms of amplitude and Signal to Noise Ratio (SNR) for the treated and the untreated ear for all patients. At group level DPOAE outcomes did not change from baseline to 6 and 12 months.

Safety – Adverse Events

Brief Summary of Adverse Events:

In total, 347 AEs were reported for 44 (100%) patients.

Of these, most AEs were of a Grade 1 (mild): 277 AEs in 44 patients (100%), followed by 70 Grade 2 (moderate) AEs in 28 patients (63.6%). There were no grade 3 (severe) AEs during or after administration of IMP.

In total, 2 SAEs were reported for 2 patients. These were both grade 2 (moderate) and not related to the study procedure or IMP. No SAEs resulted in death and none of the AEs resulted in a discontinuation from the study.

Overall, 2 AEs in 2 patients were classified as 'definitely' related to the IMP and 19 AEs in 9 patients were classified as 'definitely' related to the study procedure.

Most Frequently Reported Adverse Events:

The most frequent AEs occurred in the following MedDRA SOCs:

- *General disorders and administration site conditions: 126 events in 42 out of 44 patients (95.5%)*
- *Ear and labyrinth disorders: 94 events in 32 out of 44 patients (72.7%)*
- *Injury, poisoning and procedural complications: 32 events in 18 out of 44 patients (40.9%)*
- *Nervous system disorders: 25 events in 18 out of 44 patients (40.9%)*

On MedDRA Preferred Term (PT) levels the most frequent reported AEs were:

- *Injection site pain: 113 events in 40 out of 44 patients (90.9%)*
- *Tinnitus: 24 events in 17 out of 44 patients (38.6%)*
- *Ear congestion: 25 events in 13 out of 44 patients (29.5%)*
- *Dizziness: 12 events in 10 out of 44 patients (22.7%)*

Categorisation of All Adverse Events

Severity of All Adverse Events

Overall, most reported AEs were of a Grade 1 (mild) nature as per the investigator's opinion: 277 AEs in 44 patients. There were 70 Grade 2 (moderate) AEs reported for 28 patients. No Grade 3 (severe) AEs were reported during or after administration of IMP.

One grade 3 AE was reported for patient 322-1-011. This patient experienced a complete atrioventricular block, which was, however, observed during the screening visit, before the start of any treatment. The grade 3 AE was 'not related' to the IMP nor the procedure. The patient was not enrolled in the study.

Relationship with the Investigational Medicinal Product (Local and Systemic Adverse Events)

All patients (N = 44; 100%) experienced AEs related to the IMP (local and systemic events).

- 34 AEs classified as 'not related' to the IMP in 22 patients (50.0%)
- 61 AEs classified as 'unlikely' related to the IMP in 26 patients (59.1%)
- 149 AEs classified as 'possibly' related to the IMP in 32 patients (72.7%)
- 101 AEs classified as 'probably' related to the IMP in 30 patients (68.2%)

2 AEs were classified as 'definitely' related to the IMP and were experienced by 2 patients:

- Patient 321-1-010 experienced pain during the injection in the left (dosed) ear.
- Patient 321-1-026 experienced ear pain during the injection in the dose ear.

Relationship with the Procedure (Local and Injection Site Adverse Events)

All patients (N = 44; 100%) experienced AEs related to the procedure (injection site events).

- 41 AEs classified as 'not related' to the procedure in 23 patients (52.3%)
- 41 AEs classified as 'unlikely' related to the procedure in 20 patients (45.5%)
- 122 AEs classified as 'possibly' related to the procedure in 39 patients (88.6%)
- 124 AEs classified as 'probably' related to the procedure in 30 patients (68.2%)

19 AEs were classified as 'definitely' related to the procedure in 9 patients. Most commonly reported events were pain during the injection in the dosed ear, sensation of a blocked ear, or throat pain.

<u>Post-hoc exploratory analysis and mixed-effect model</u>

Methods

A three-level linear mixed-effect model was used to account for repeated measures and the multilevel structure of the pure-tone audiometry and speech perception in noise data at 6 and 12 months. Patient age, baseline audiometric values, follow-up timepoint, and timepoint-by-treatment interaction were entered as fixed factors. Patient (random intercept) and timepoint (random slope) were entered as random factors. An unstructured covariance matrix was used.

For pure- tone audiometry we analysed the mean change from baseline to 6 and 12 months the average pure-tone threshold across 2, 4, and 8 kHz, as well as at individual frequencies, including 12.5 and 16 kHz. In addition, we analysed at 6 and 12 months the number and percentage of participants showing: a) a positive change of ≥10 dB in pure-tone air conduction- threshold at any frequency, and b) a positive change of ≥10 dB in pure-tone air-conduction threshold in two or more adjacent frequencies, or ≥20 dB in a single frequency. Finally, we defined consistent hearing improvement over time as a positive change of ≥10 dB at a certain frequency which was also present at two or more consecutive follow-up visits.
For speech perception in noise we analysed the mean change in SRT50n from baseline to 6 and 12 months. In addition, we analysed at 6 and 12 months the number and percentage of participants showing a positive change of ≥2 dB in the SRTn50.

For Otoacoustic emissions, a repeated measures ANCOVA was conducted for each DPOAE frequency on changes in amplitudes and/or SNRs from baseline to 6 and 12 weeks, 6 months and 12 months. Timepoint, treatment, timepoint-by-treatment interaction were within-patient factors; trial site was the between-patient factor, and baseline values were covariates. Due to data complexity, a clinically relevant change was defined as a statistically significant estimated mean change in DPOAE amplitude or SNR (repeated measures ANCOVA) from baseline to 2 or more consecutive follow-up timepoints. A Wald-Wolfowitz Runs Test and a Bernoulli-statistics test for Randomness were additionally conducted. Analyses were performed with SAS (v9.4), SPSS (v26), and R (v3.3.1).

Results Pure-Tone Audiometry at 6 and 12 months

The mean pure-tone threshold across 2, 4, and 8 kHz did not change significantly from baseline to 6 months in the treated ear (estimated change -0.44 dB; 95% confidence interval [CI] -2.01 to 1.13; P=0.578) nor from baseline to 12 months (-0.05 dB; CI -2.01 to 1.90; P=0.956).

Nineteen patients (N=38; 50%) showed an improvement of ≥10 dB in one or more individual frequencies (including 12.5 and 16 kHz) from baseline to 6 months and 14 (N=32; 44%) from baseline to 12 months. Six patients (N=38; 17%) showed an improvement of ≥10 dB in two or more adjacent frequencies, or of ≥20 dB in one or more individual frequencies at 6 months and 6 patients (N=32; 19%) at 12 months.

Results Speech Perception in Noise at 6 and 12 months

The mean speech reception threshold in noise, measured with a words-in-babble test and expressed as the SRT50n, did not change from baseline to 6 months (estimated change -0.19 (CI -1.23 – 0.86), P=0.724) and 12 months (estimated change 0.20 (CI -1.23 – 1.62), P=0.780) in the treated ear. Ten patients (N=38; 26%) showed an improvement in SRT50n of ≥2 dB at 6 months and 8 patients (N=32; 25%) at 12 months.

Figure S1 Pure-tone audiograms of all patients that completed 12 weeks and 6 and 12 months follow up

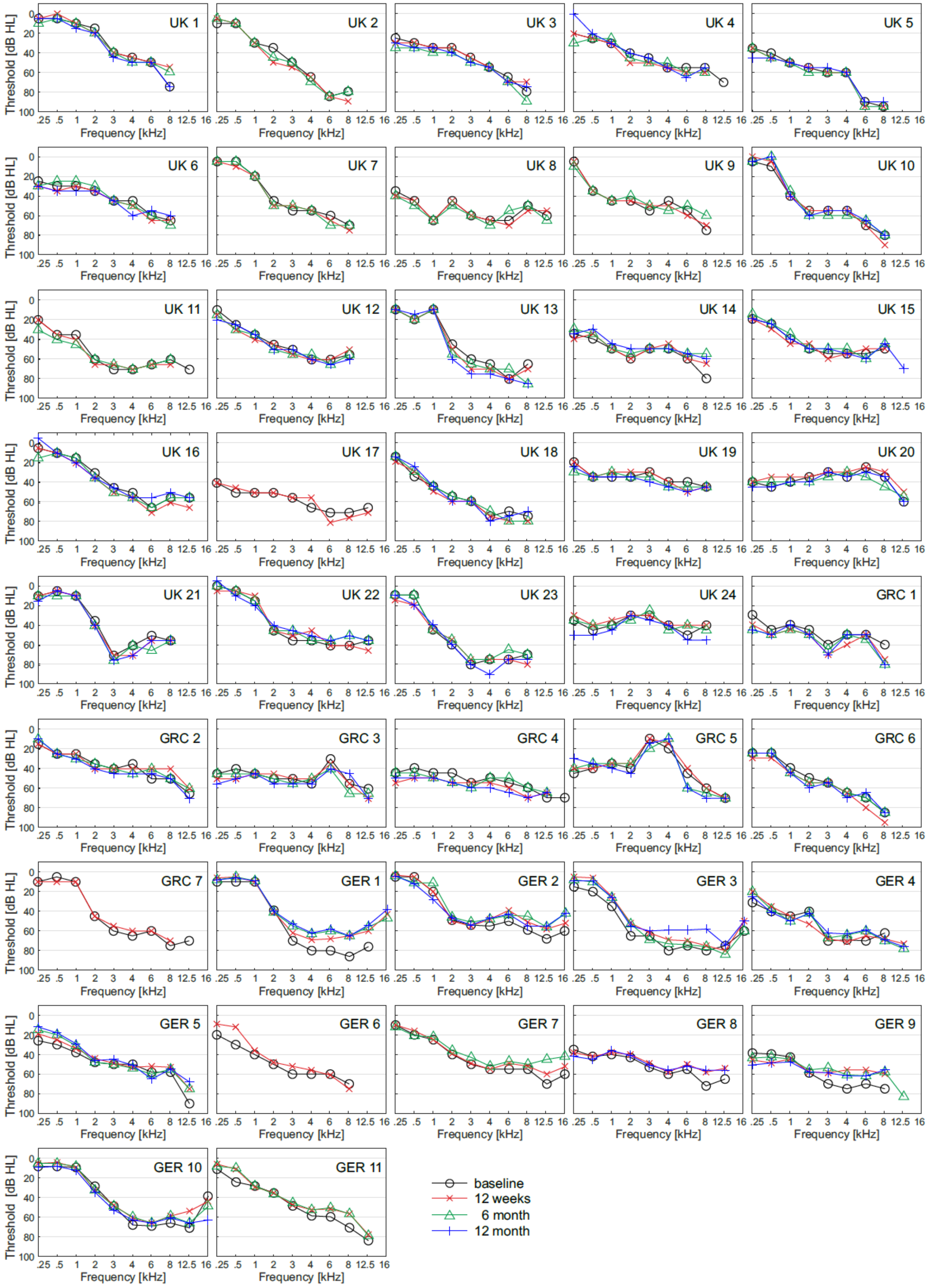

Presented timepoints are baseline, 12 weeks (N= 42), 6 month (N= 38) and 12 months (N=32).

Frequencies not shown indicate that thresholds are beyond the maximum output of the audiometer.

Supplement on Distortion Product Otoacoustic Emissions

The accurate estimation of the major DPOAE measures, namely the amplitudes and the SNRs, rely heavily on the acoustic conditions during the acquisition and the processing algorithm of the OAE signals (Dillier, 2005), (Zelle et al., 2017).

In clinical diagnosis frameworks and practice, achieving clear-cut evaluations and assessment of the DP indices, regardless of measuring equipment specificities, is traditionally pursued by imposing specific and strict acceptability thresholds for the background acoustic noise and the minimum distortion of the measurement systems as well as criteria for the interpretation of the obtained DPOAE measures. Additionally, as the clinical interpretation of DPOAE is often done in line to a “gold standard” test of hearing acuity such as the PTA, DP amplitudes and/or SNRs are often demanded to comply with specified ranges of values that “ensure” unambiguous and reliable depiction of the DP mechanisms.

In this manner, DPOAE amplitudes < -20dBSPL are considered absent, regardless of the SNR (Reavis et al., 2011), (Dhar & Hall III, 2011). This limit stems from measurements of distortion of typical DPOAE recording systems (Reavis et al., 2011), (Gorga et al., 1994), which might otherwise obscure the detection of natural OAE spectral spikes. However, it must be pointed out that the level of distortion from the measurement system is dependent upon the level of primaries and it is often much lower than -20dBSPL (Gorga et al., 1994), (Reavis et al., 2011).

Additionally, it is commonly accepted that DPOAE amplitudes at SNRs > 6dB are considered valid and the DPs are also interpreted as “present” if their level is higher than -20dBSPL (the system distortion limit mentioned previously). Indeed, as the SNR increases so does the confidence that the measured DP amplitude is closer to the true value, which strengthens the clinical diagnosis.

Despite the obvious advantages for measurements’ reliability, very often “the use of such criteria often results in a loss of data points” (Dhar et al., 1998) and impedes several analytical and methodological approaches. Moreover, commonly, contemporary DPOAE measurement equipment produce estimates of DPOAE amplitudes through a process of time domain averaging which aims at “cleaning” the recordings of noise (Zelle et al., 2017), (Interacoustics, 2019), (Zelle et al., 2017) and, thus, increase the validity of the DP estimates and allow for equipment-reported DP amplitudes even lower than the above-mentioned typical distortion floor of -20dBSPL.

Under these considerations, and since the purpose of the current research is to investigate the full range of the DP responses, beyond the typically applied validity criteria for the diagnostic practice (e.g., SNR > 6dB), our analysis includes all available information on DPOAE amplitudes/SNR, avoiding any strategy for rejection of specific ranges of data values.

To further scrutinize the DP responses, even beyond the readings of the DPOAE equipment (obtained through the averaging process mentioned previously), we repeated our analysis, but now, by using DPOAE amplitudes estimates which also take into account the estimated SNRs and the number of averaged epochs of the recorded signals (Dhar et al., 1998), (Backus, 2007). The details on obtaining these "adapted" DPOAE amplitudes estimates are given in Attachment 1.

In order to complement the investigation of randomness of patterns (along DP frequencies) of the distributions of clinically relevant improvements/deteriorations, in addition to the Wald-Wolfowitz Runs-test, we also employ a test based on indices of Bernoulli/Poisson statistics. The details on this test are given in Attachment 2.

In the following sections we present and compare the results of the analyses of both the original DP amplitudes/SNRs data and the DP amplitudes estimates after the adaptations for SNR and number of signal averaging epochs

Section 1: Analysis based on original DP amplitude/SNR data

The post-hoc repeated measures ANCOVA was conducted for each DPOAE frequency on changes in amplitudes and SNRs from baseline to 6 weeks and 3, 6 and 12 months (namely 6, 12, 24 and 48 weeks), with timepoint, treatment, and timepoint-by-treatment interaction were within-patient factors; trial site between-patient factor, and age, baseline values as covariates. The analysis was conducted separately for low- and high-level tone primaries.

For brevity, Table S3 presents statistically significant ($p \leq 0.05$) effects only. In each cell, the numbers represent the $partial - \eta^2$ values of effect for the respective covariates or factors. The cells' color represents typical ranges of effect size ($\eta^2 \geq 0.14$: large, $0.06 \leq \eta^2 < 0.14$: medium, and $\eta^2 < 0.06$: small effect size). The baseline values and the trial site consistently showed larger effects, with timepoint, age and ear showing smaller effects.

Figure S2 shows the mean estimated changes (+/- 2SE) from baseline of DP amplitudes and SNRs, at each frequency for the treated and untreated ear, high-level and low-level primaries and across trial sites and along timepoints. From these data, the heatmap (Figure S3) depicts for each frequency the average of mean estimated changes from baseline of the DP amplitudes and SNRs, at the timepoints that met the criteria of "clinically relevant" changes (see main text, Methods-Statistical Analysis section). Improvements (i.e., positive changes from baseline) are shown in green and deteriorations (i.e., negative changes from baseline) are shown in red. At low-level tone primaries, improvements in the treated ear were detected at most frequencies below 1200 Hz, whereas in high-level tone primaries, an improvement in the treated ear may be observed at 4-5kHz. However, there were also some reductions in SNRs in the treated ear, mainly in the high-level primaries, mostly below 2kz.

Figure S4 shows both the averages and the 95%CIs of the “clinically relevant” changes of Figure S3. Note that neither of the 95%CIs of “clinically relevant” improvements include 0, whereas almost all of the 95%CIs of the deteriorations include 0. This implies an increased level of confidence of true improvement at frequencies where positive average DP changes are observed compared to a lower level of confidence for true deterioration at frequencies where negative average DP changes are observed.

The frequency patterns of “clinically relevant” improvements and deteriorations were tested for randomness using a Wald-Wolfowitz Runs-test (each test with 10million bootstraps), in order to estimate the cumulative probability function of number of runs $\boldsymbol{P_{cum}(r)}$, in the case of random patterns. Figure S4 shows the probability function $\boldsymbol{P(r)}$ and the cumulative distribution function $\boldsymbol{P_{cum}(r)}$;the diamond markers show the $\boldsymbol{P_{cum}(r_0)}$, $\boldsymbol{r_0}$=number of runs in the pattern in each case. These results show that the probability of randomness of appearance of the frequency patterns of “clinically relevant” improvement was lowest in the treated ear, especially in DPOAE amplitudes at low-level primaries [$\boldsymbol{P_{cum}(r_0)}$=0.086, $\boldsymbol{r_0}$=9], and DPOAE amplitudes [$\boldsymbol{P_{cum}(r_0)}$=0.20, $\boldsymbol{r_0}$=9] and SNR [$\boldsymbol{P_{cum}(r_0)}$=0.16, $\boldsymbol{r_0}$=5] at high-level primaries. The respective cumulative probabilities of randomness in case of deteriorations were larger than 0.35.

Using the Bernoulli-based tests (Figure S6) similar results with the Runs-test were obtained for the improvement, namely, possibly non-random patterns were identified only at the treated ear and both low-level and high-level primaries regarding the DP amplitudes, and in the treated ear’s SNRs at high-level primaries. Characteristic deteriorations were only detected in the SNRs at the treated ear and at high-level primaries.

Table S3 (A) Statistically significant effects of the Repeated Measures ANCOVA, per DPOAE frequency (DP amplitudes at low-level primaries)

A

Frequency (Hz)	Age	Baseline	Clinic	Ear(T/U)	Time	Ear(T/U) *by* Age	Ear(T/U) *by* Baseline	Ear(T/U) *by* Clinic	Time *by* Age	Time *by* Baseline	Time *by* Clinic	Time *by* Ear(T/U)	Time *by* Ear(T/U) *by* Age	Time *by* Ear(T/U) *by* Baseline	Time *by* Ear(T/U) *by* Clinic
498		0.28	0.34												
547		0.23	0.48					0.12							
596	0.16	0.18	0.22		0.06			0.12				0.05			
645		0.17	0.34	0.07							0.07				
703		0.23	0.24									0.09	0.10		0.14
771		0.36	0.23												
840		0.23	0.19		0.05										
918		0.42	0.22	0.13	0.08	0.17	0.07		0.09		0.09				0.08
996		0.43	0.40								0.07			0.06	
1094		0.27	0.15						0.05						0.08
1191		0.14	0.27												
1299	0.08	0.13	0.14				0.09	0.26							
1416		0.45	0.28		0.09				0.08						
1543	0.08	0.24	0.14									0.05			0.12
1680		0.31	0.14		0.09				0.10			0.05	0.06		0.11
1836		0.31	0.28		0.15			0.12	0.18						0.15
2002		0.46	0.30		0.15				0.15		0.08		0.05		0.12
2178		0.50	0.34												
2383		0.42	0.33	0.07		0.12		0.16				0.06	0.07		
2598		0.48	0.18		0.07				0.08						
2832		0.13	0.11								0.11	0.05	0.05		0.11
3086		0.40	0.16								0.11				
3359	0.11	0.26	0.19									0.06	0.09	0.07	
3672		0.28	0.12						0.05		0.08				
4004	0.07	0.21	0.37						0.05		0.14				0.12
4365		0.20	0.12								0.20				
4756		0.32	0.16					0.12			0.10	0.05	0.07		0.10
5186		0.23	0.29	0.11		0.09					0.18				0.09
5654		0.31	0.20								0.12	0.15	0.15		
6172		0.30	0.35	0.14		0.11					0.15	0.06	0.09		0.15
6729		0.35	0.29												
7334		0.35	0.13		0.05				0.11		0.16	0.14	0.16		0.08
7998	0.12	0.40	0.27			0.09					0.12	0.07	0.06		
8721		0.42	0.40											0.06	0.12
9512		0.46	0.34				0.11					0.20	0.17	0.05	0.10

(A) DP amplitudes at low-level primaries. Effect sizes of statistically significant factors and covariates of the Repeated Measures ANCOVA, per DPOAE frequency ($p \leq 0.05$). Values in cells are the $partial - \eta^2$ values of effect and colors represent categories of effect size (vivid: large, mid: medium, pale: small). All tests were two-sided, without multiple-comparisons correction. After Bonferonni corrections, none of these effect sizes were statistically significant.

Table S3 (B) Statistically significant effects of the Repeated Measures ANCOVA, per DPOAE frequency (DP SNRs at low-level primaries)

B

Frequency (Hz)	Age	Baseline	Clinic	Ear(T/U)	Time	Ear(T/U) *by* Age	Ear(T/U) *by* Baseline	Ear(T/U) *by* Clinic	Time *by* Age	Time *by* Baseline	Time *by* Clinic	Time *by* Ear(T/U)	Time *by* Ear(T/U) *by* Age	Time *by* Ear(T/U) *by* Baseline	Time *by* Ear(T/U) *by* Clinic
498		0.43												0.08	0.15
547	0.09	0.46	0.20					0.11				0.09	0.08		
596	0.11	0.28	0.17		0.06				0.11		0.14	0.05			0.11
645		0.39	0.15												
703		0.40									0.08	0.06	0.07		0.18
771		0.37	0.11								0.08				0.07
840		0.30			0.05										
918		0.29			0.08										0.09
996		0.42										0.05	0.05		
1094		0.36					0.11	0.11		0.05				0.08	0.09
1191		0.18		0.10				0.20	0.05						
1299		0.20						0.18		0.06					
1416		0.36			0.09				0.05	0.05		0.07	0.06	0.05	
1543		0.26								0.06	0.20			0.06	0.17
1680		0.29			0.09				0.07						0.07
1836		0.40	0.19		0.15			0.25							0.15
2002		0.44			0.15				0.13		0.07	0.06	0.07		0.14
2178	0.07	0.31								0.06					
2383		0.51	0.11					0.18			0.14	0.15	0.12	0.07	0.09
2598		0.47			0.07				0.05	0.06	0.11				0.08
2832		0.23	0.12					0.10							0.11
3086		0.45												0.06	0.08
3359		0.41													
3672		0.27								0.06					
4004	0.11	0.18	0.12						0.11	0.06	0.15				0.08
4365	0.15	0.34									0.12				
4756	0.11	0.39					0.08	0.13		0.06	0.10	0.05	0.06		0.08
5186	0.13	0.22									0.13				0.08
5654		0.19									0.14	0.19	0.18		0.08
6172		0.25		0.11		0.13					0.09	0.13	0.14		0.14
6729		0.37													0.10
7334		0.32			0.05						0.12		0.05		0.09
7998		0.34									0.09				
8721		0.43	0.14											0.06	0.18
9512		0.47	0.13									0.13	0.15	0.05	0.08

(B) DP SNRs at low-level primaries. Effect sizes of statistically significant factors and covariates of the Repeated Measures ANCOVA, per DPOAE frequency (p≤0.05). Values in cells are the $partial-\eta^2$ values of effect and colors represent categories of effect size (vivid: large, mid: medium, pale: small). All tests were two-sided, without multiple-comparisons correction. After Bonferonni corrections, none of these effect sizes were statistically significant.

Table S3 (C) Statistically significant effects of the Repeated Measures ANCOVA, per DPOAE frequency (DP amplitudes at high-level primaries)

C

Frequency (Hz)	Age	Baseline	Clinic	Ear(T/U)	Time	Ear(T/U) *by* Age	Ear(T/U) *by* Baseline	Ear(T/U) *by* Clinic	Time *by* Age	Time *by* Baseline	Time *by* Clinic	Time *by* Ear(T/U)	Time *by* Ear(T/U) *by* Age	Time *by* Ear(T/U) *by* Baseline	Time *by* Ear(T/U) *by* Clinic
498		0.44	0.27								0.15	0.05	0.05	0.06	
547		0.34	0.19								0.14				
596		0.09		0.10		0.08		0.20						0.05	0.10
645		0.27	0.20					0.11			0.17			0.14	0.15
703		0.28	0.28					0.12				0.06	0.06		
771		0.37	0.20							0.06	0.09	0.17	0.18		
840		0.25	0.20					0.12			0.10				
918		0.25	0.35		0.10				0.11						
996		0.27	0.26	0.11		0.09			0.05	0.10	0.14				
1094		0.28	0.28		0.09				0.11						
1191	0.13		0.32								0.10	0.05	0.06		
1299		0.31	0.31								0.08			0.05	
1416		0.33	0.35						0.05	0.06	0.11				
1543		0.37	0.26							0.05	0.12	0.07	0.07		
1680	0.07	0.16	0.26	0.11	0.05	0.16			0.06	0.06	0.10				
1836		0.50	0.58				0.08			0.06	0.08	0.07	0.07		
2002		0.35	0.26								0.12				
2178		0.38	0.17					0.12							
2383		0.52	0.29									0.08	0.08		
2598		0.41	0.14		0.08				0.08			0.06			
2832		0.40		0.14		0.11					0.10	0.13	0.12	0.09	0.13
3086		0.38	0.19								0.22	0.06	0.06		0.12
3359		0.43									0.11				0.08
3672		0.37			0.05						0.13	0.05	0.06		
4004		0.22						0.12		0.05	0.32	0.13	0.14	0.07	0.13
4365		0.30									0.28	0.14	0.15		
4756		0.20			0.06						0.16		0.05		
5186					0.10			0.14	0.07		0.15				
5654		0.09						0.17			0.10	0.06			
6172		0.32				0.10					0.08				0.10
6729		0.52	0.26		0.09				0.09						
7334		0.40			0.09			0.23	0.07			0.06			
7998	0.10	0.35					0.14				0.14			0.09	
8721		0.19												0.09	
9512	0.18	0.10	0.11		0.09			0.09	0.10						

(C) DP amplitudes at high-level primaries. Effect sizes of statistically significant factors and covariates of the Repeated Measures ANCOVA, per DPOAE frequency ($p \leq 0.05$). Values in cells are the $partial-\eta^2$ values of effect and colors represent categories of effect size (vivid: large, mid: medium, pale: small). All tests were two-sided, without multiple-comparisons correction. After Bonferonni corrections, none of these effect sizes were statistically significant.

Table S3 (D) Statistically significant effects of the Repeated Measures ANCOVA, per DPOAE frequency (DP SNRs at high-level primaries)

D

Frequency (Hz)	Age	Baseline	Clinic	Ear(T/U)	Time	Ear(T/U) *by* Age	Ear(T/U) *by* Baseline	Ear(T/U) *by* Clinic	Time *by* Age	Time *by* Baseline	Time *by* Clinic	Time *by* Ear(T/U)	Time *by* Ear(T/U) *by* Age	Time *by* Ear(T/U) *by* Baseline	Time *by* Ear(T/U) *by* Clinic
498		0.45	0.13					0.14			0.08				
547		0.43	0.11		0.11			0.13	0.10						
596		0.16													
645		0.40			0.06			0.14	0.07		0.09			0.08	
703		0.35			0.08			0.13	0.06	0.07					
771		0.27										0.14	0.15		
840		0.34									0.11		0.05		
918		0.22	0.13		0.11			0.16	0.11						0.10
996		0.36								0.09		0.07			
1094		0.32			0.15				0.17		0.09				
1191	0.08		0.23								0.08				
1299		0.31													
1416		0.18			0.05					0.05					0.15
1543		0.30									0.08	0.05	0.05		0.13
1680		0.31						0.17							
1836	0.11	0.45	0.21		0.12			0.11	0.10	0.05	0.08	0.05	0.05	0.05	
2002		0.34	0.13				0.08				0.08	0.07	0.07		
2178		0.48									0.08				
2383	0.09	0.56			0.05						0.24				
2598		0.46			0.07				0.06		0.16	0.07	0.06		
2832		0.49			0.06				0.06		0.15				
3086		0.32									0.17	0.06			0.12
3359		0.29			0.07			0.14	0.06			0.09	0.06		0.10
3672		0.41							0.05		0.13	0.05			
4004		0.21									0.18				0.10
4365		0.19	0.20								0.18	0.10	0.12		0.09
4756		0.10									0.26				
5186			0.26		0.06			0.21	0.08		0.11				0.13
5654		0.20						0.15			0.13				
6172	0.09	0.36	0.18												0.12
6729		0.43	0.22		0.07				0.06		0.08				
7334	0.08	0.32			0.07			0.15	0.06		0.09				
7998	0.11	0.37		0.11			0.16	0.10			0.08				
8721	0.10	0.12				0.09						0.09		0.08	0.08
9512	0.23	0.09	0.11						0.09		0.07				

(D) DP SNRs at high-level primaries. Effect sizes of statistically significant factors and covariates of the Repeated Measures ANCOVA, per DPOAE frequency (p≤0.05). Values in cells are the $partial - \eta^2$ values of effect and colors represent categories of effect size (vivid: large, mid: medium, pale: small). All tests were two-sided, without multiple-comparisons correction. After Bonferonni corrections, none of these effect sizes were statistically significant.

Figure S2 (A) Change from baseline: DPOAE Amplitudes/ Low-level primaries

A

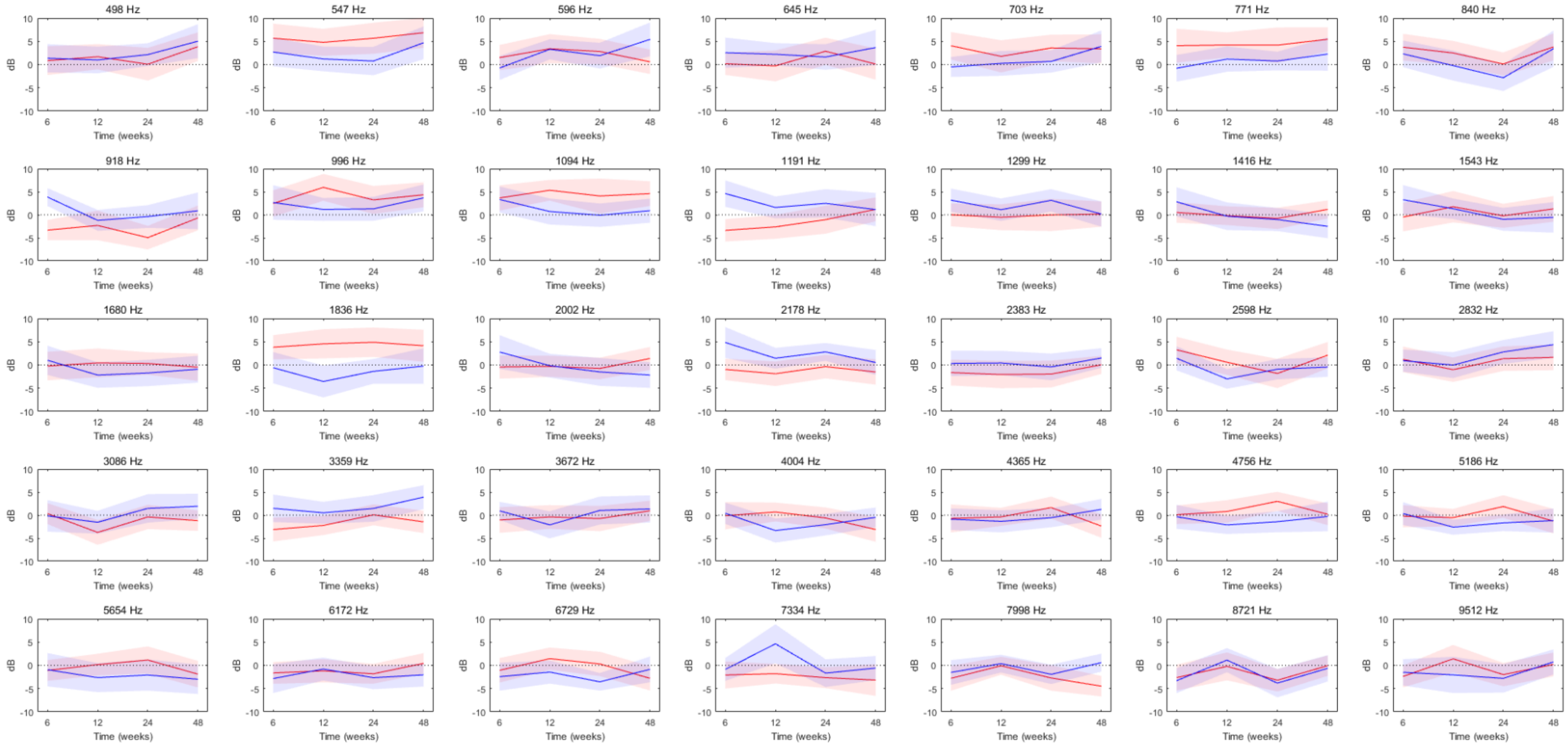

(A) DP amplitudes at low-level primaries. Mean estimated changes (shadings: +/- 2SE) from baseline at each frequency, across trial sites and along timepoints 6 weeks, 3, 6 and 12 months (namely 6, 12, 24 and 48 weeks). Red lines/shadings: treated ear, and blue lines/shadings: untreated ear.

Figure S2 (B) Change from baseline: DPOAE SNRs / Low-level primaries

B

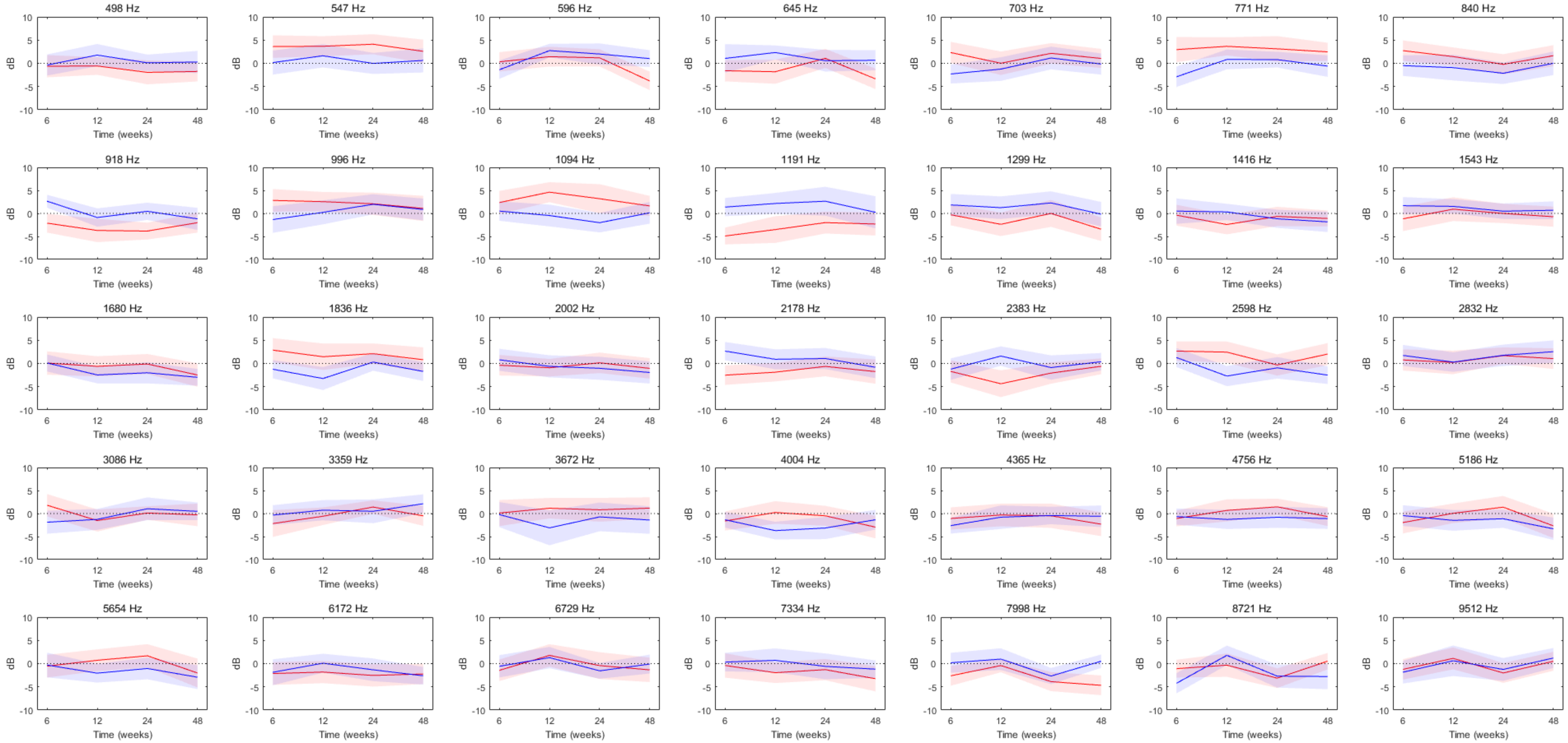

(B) DP SNRs at low-level primaries. Mean estimated changes (shadings: +/- 2SE) from baseline at each frequency, across trial sites and along timepoints 6 weeks, 3, 6 and 12 months (namely 6, 12, 24 and 48 weeks). Red lines/shadings: treated ear, and blue lines/shadings: untreated ear.

Figure S2 (C) Change from baseline: DPOAE Amplitudes/ High-level primaries

C

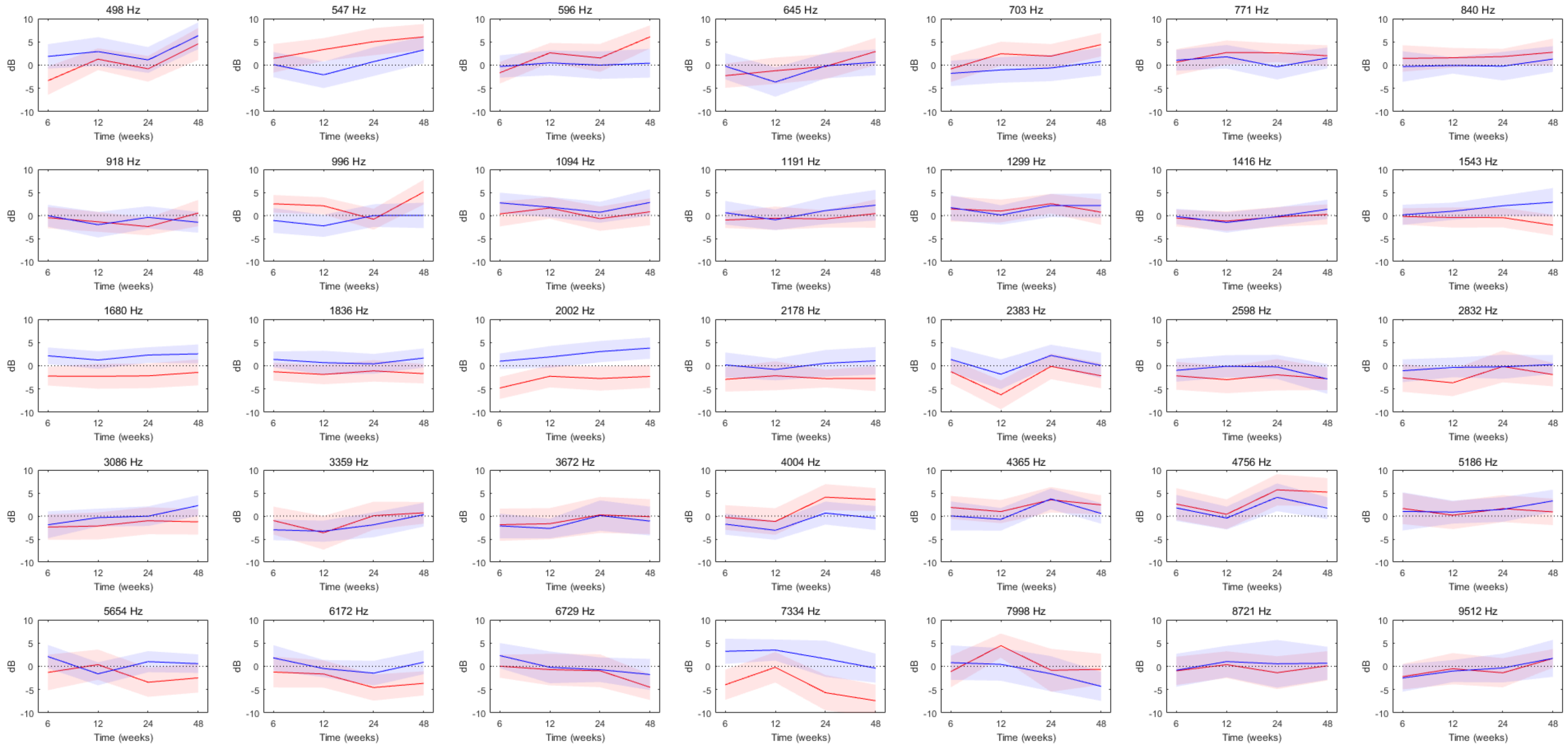

(C) DP amplitudes at high-level primaries. Mean estimated changes (shadings: +/- 2SE) from baseline at each frequency, across trial sites and along timepoints 6 weeks, 3, 6 and 12 months (namely 6, 12, 24 and 48 weeks). Red lines/shadings: treated ear, and blue lines/shadings: untreated ear.

Figure 2 (D) Change from baseline: DPOAE SNRs/ High-level primaries

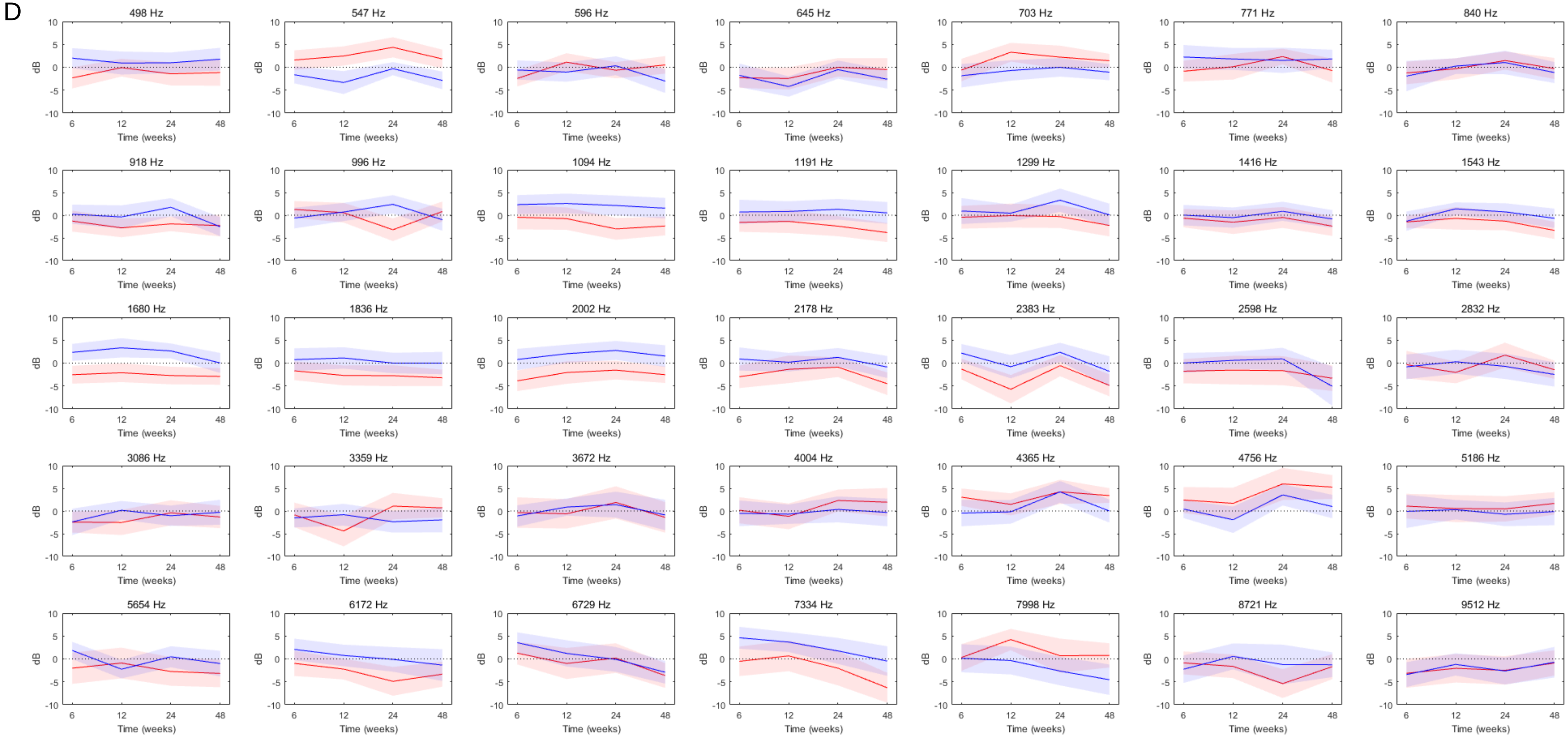

(D) DP SNRs at high-level primaries. Mean estimated changes (shadings: +/- 2SE) from baseline at each frequency, across trial sites and along timepoints 6 weeks, 3, 6 and 12 months (namely 6, 12, 24 and 48 weeks). Red lines/shadings: treated ear, and blue lines/shadings: untreated ear.

Figure S3 "Clinically relevant" changes of the DP amplitudes and SNRs

	Low-level primaries				High-level primaries			
	Amplitude		SNR		Amplitude		SNR	
Frequency (Hz)	Treated ear	Untreated ear	Treated ear	Untreated ear	Treated ear	Untreated ear	Treated ear	Untreated ear
498						3.6		
547	5.7		3.5		4.8		3.4	
596	3.1							
645							-2.4	
703	3.5							
771	4.5		3		2.7			
840								
918			-3.2				-2.3	
996	4.5		2.7		2.3			
1094	4.4		3.9				-2.7	2.5
1191	-3		-4.2				-3.1	
1299								
1416								
1543								
1680						2.42	-2.6	2.8
1836	4.3						-2.9	
2002						3.4		2.4
2178					-2.7			
2383								
2598			2.5					
2832		3.6						
3086								
3359	-2.7					-3.1		
3672								
4004				-3.4	3.8			
4365					3		3.8	
4756					5.4		5.6	
5186								
5654								
6172			-2.4		-4.1		-4.1	
6729								
7334					-6.5	3.4		4.1
7998	-3.6		-4.3					
8721				-2.7				
9512								

Graded heatmap and average values of "clinically relevant" changes of the DP amplitudes and SNRs (improvements shown in green, deteriorations in red).

Figure S4 (A) 'clinically relevant' DPOAE improvement

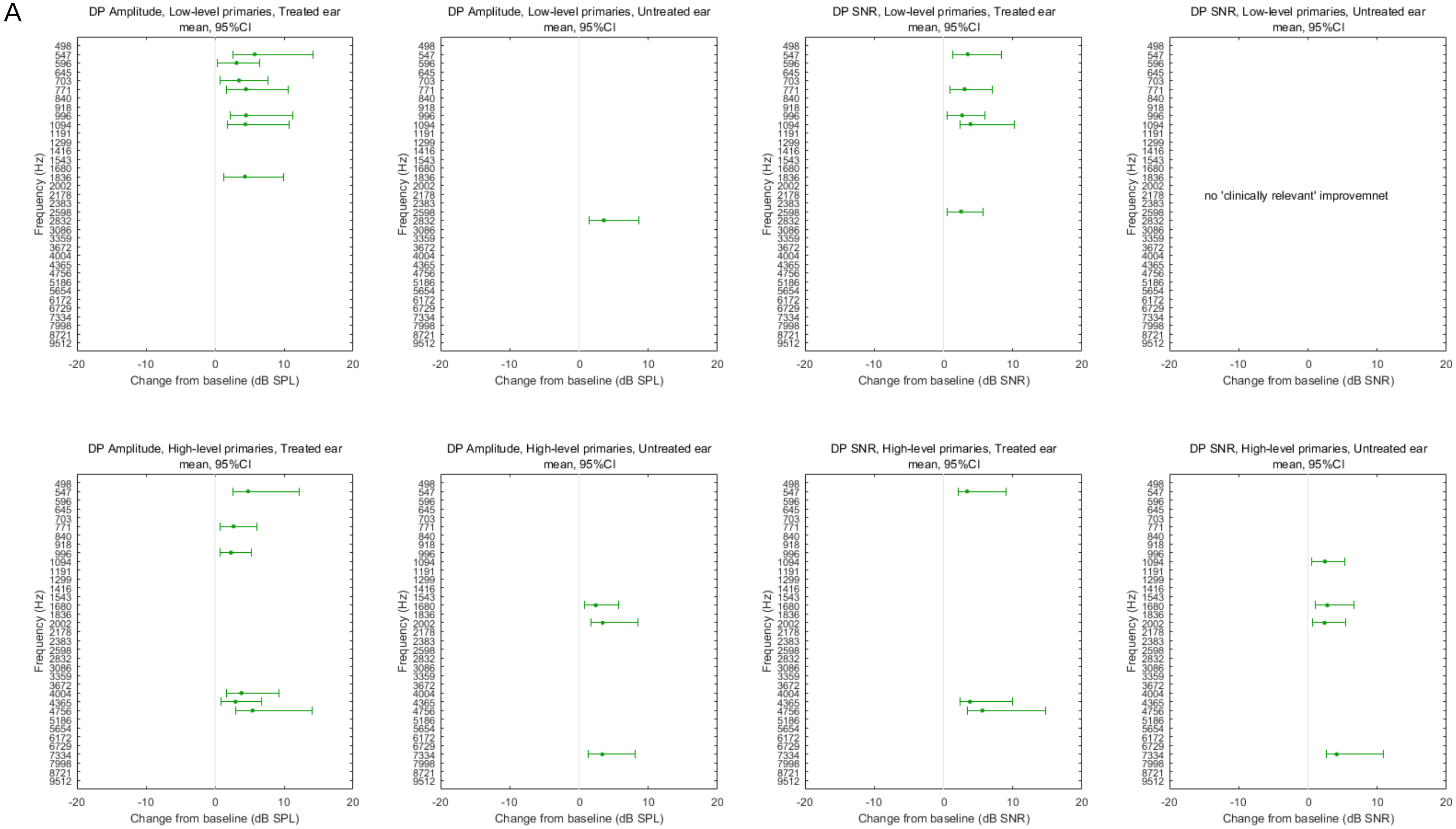

Average and 95%CI of "clinically relevant" improvements for DP amplitudes and SNRs, low-level and high-level primaries and treated/untreated ear.

Figure S4 (B) ‘clinically relevant’ DPOAE deterioration

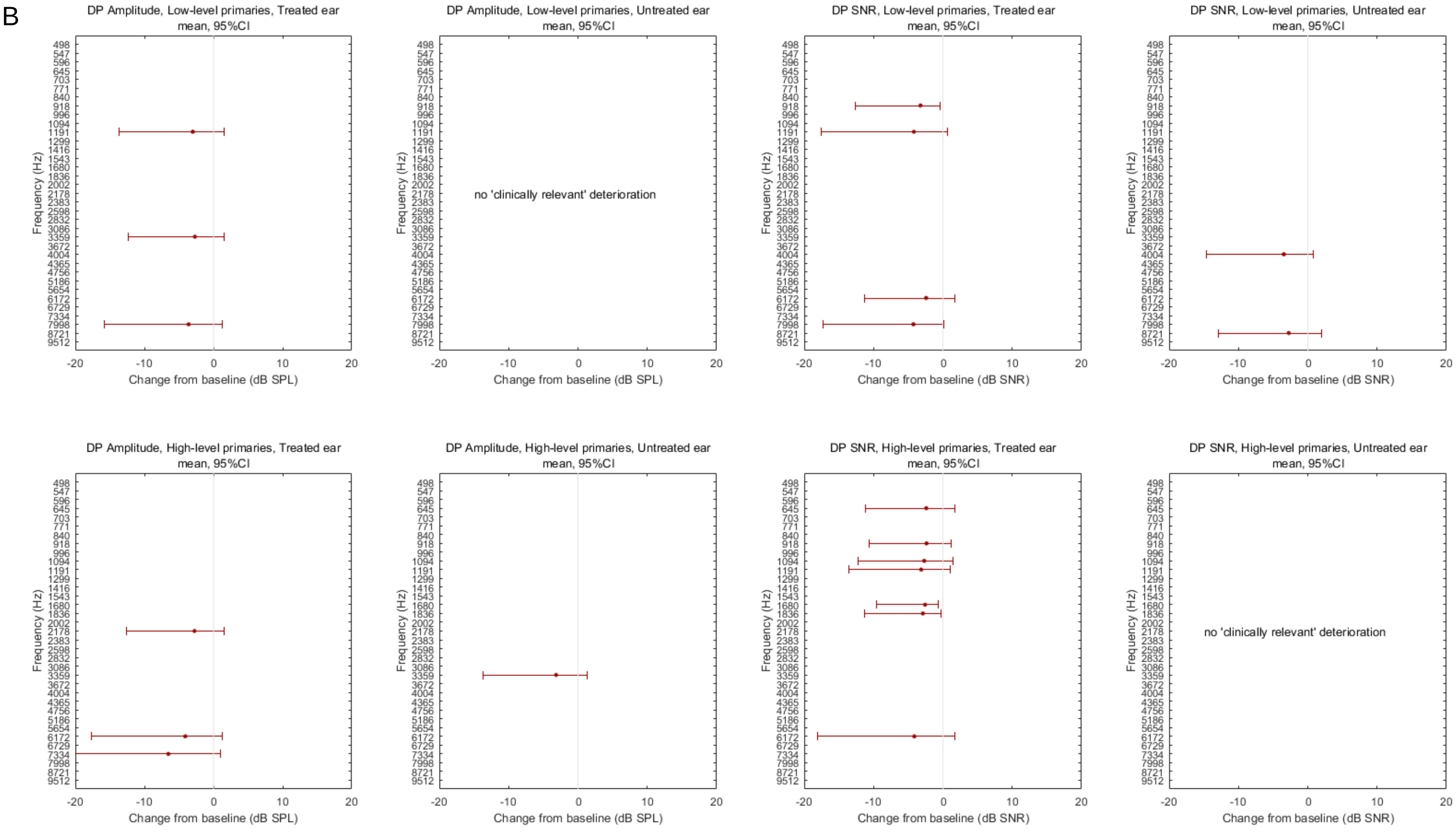

Average and 95%CI of “clinically relevant” deteriorations for DP amplitudes and SNRs, low-level and high-level primaries and treated/untreated ear.

Figure S5 (A) Runs-test for pattern randomness of ‘clinically relevant DPOAE improvements

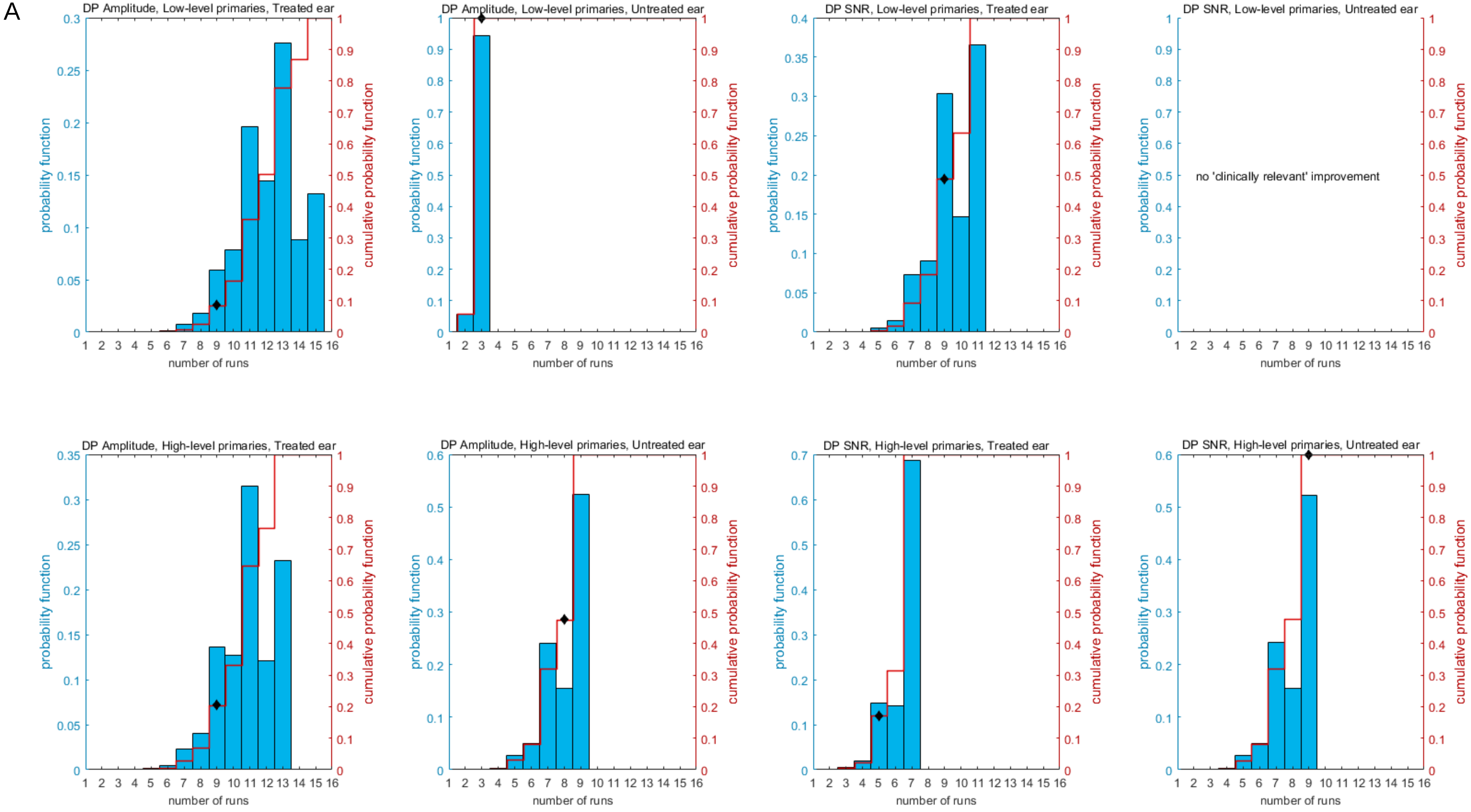

Probability functions $\boldsymbol{P}(\boldsymbol{r})$ and cumulative distribution functions $\boldsymbol{P}_{cum}(\boldsymbol{r})$ (red lines) for the Wald-Wolfowitz Runs-test for randomness of appearance of frequency patterns of “clinically relevant” **improvements.** The diamond markers mark the $\boldsymbol{P}_{cum}(\boldsymbol{r}_0)$, $\boldsymbol{r}_0$=number of runs in the pattern.

Figure S5 (B) Runs-test for pattern randomness of ‘clinically relevant DPOAE deterioration

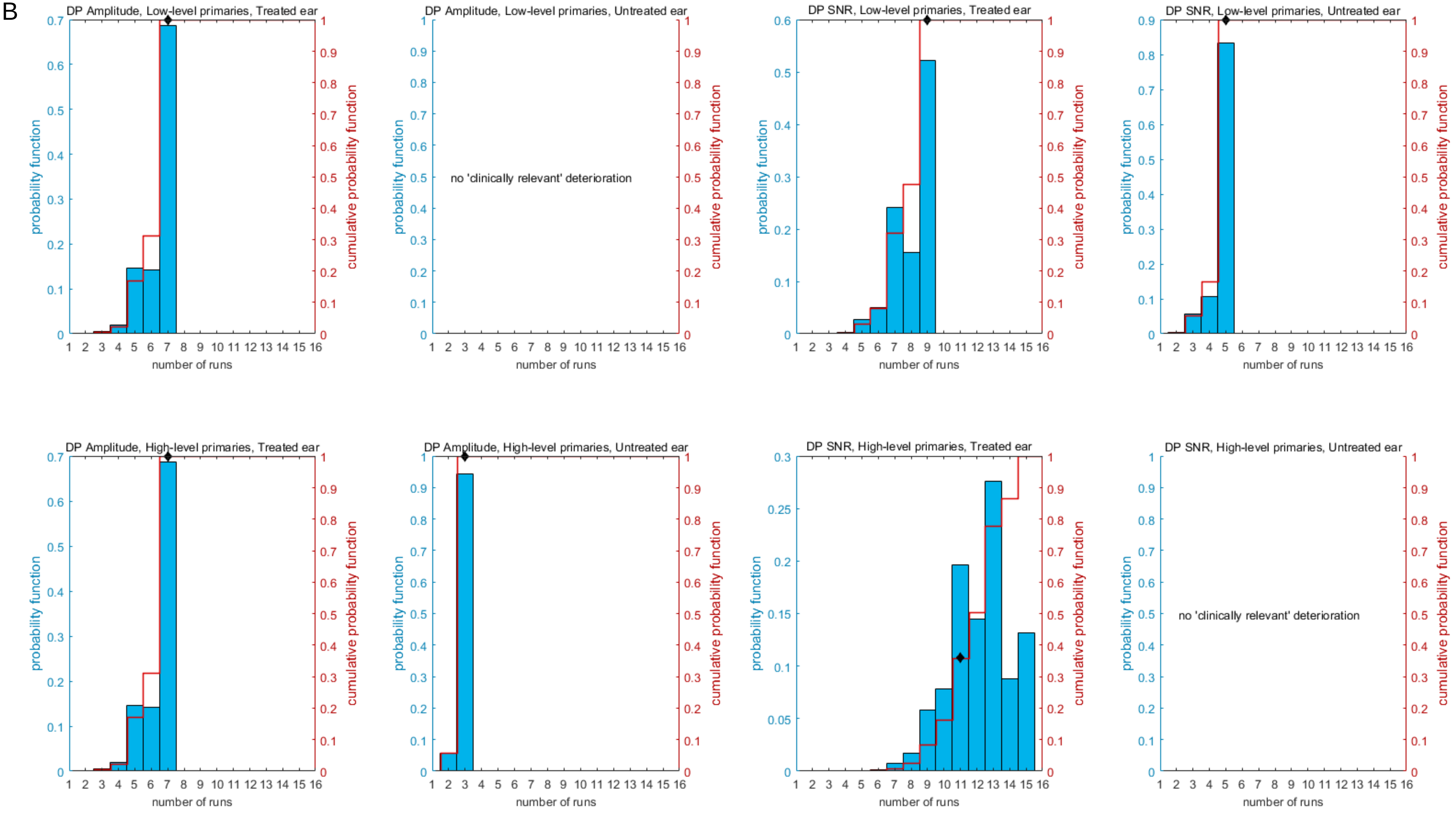

Probability functions $\boldsymbol{P}(\boldsymbol{r})$ and cumulative distribution functions $\boldsymbol{P}_{\boldsymbol{cum}}(\boldsymbol{r})$ (red lines) for the Wald-Wolfowitz Runs-test for randomness of appearance of frequency patterns of “clinically relevant” **deteriorations**. The diamond markers mark the $\boldsymbol{P}_{\boldsymbol{cum}}(\boldsymbol{r_0})$, $\boldsymbol{r_0}$=number of runs in the pattern.

Figure S6 (A) Bernoulli-test for pattern randomness of ‘clinically relevant DPOAE improvement

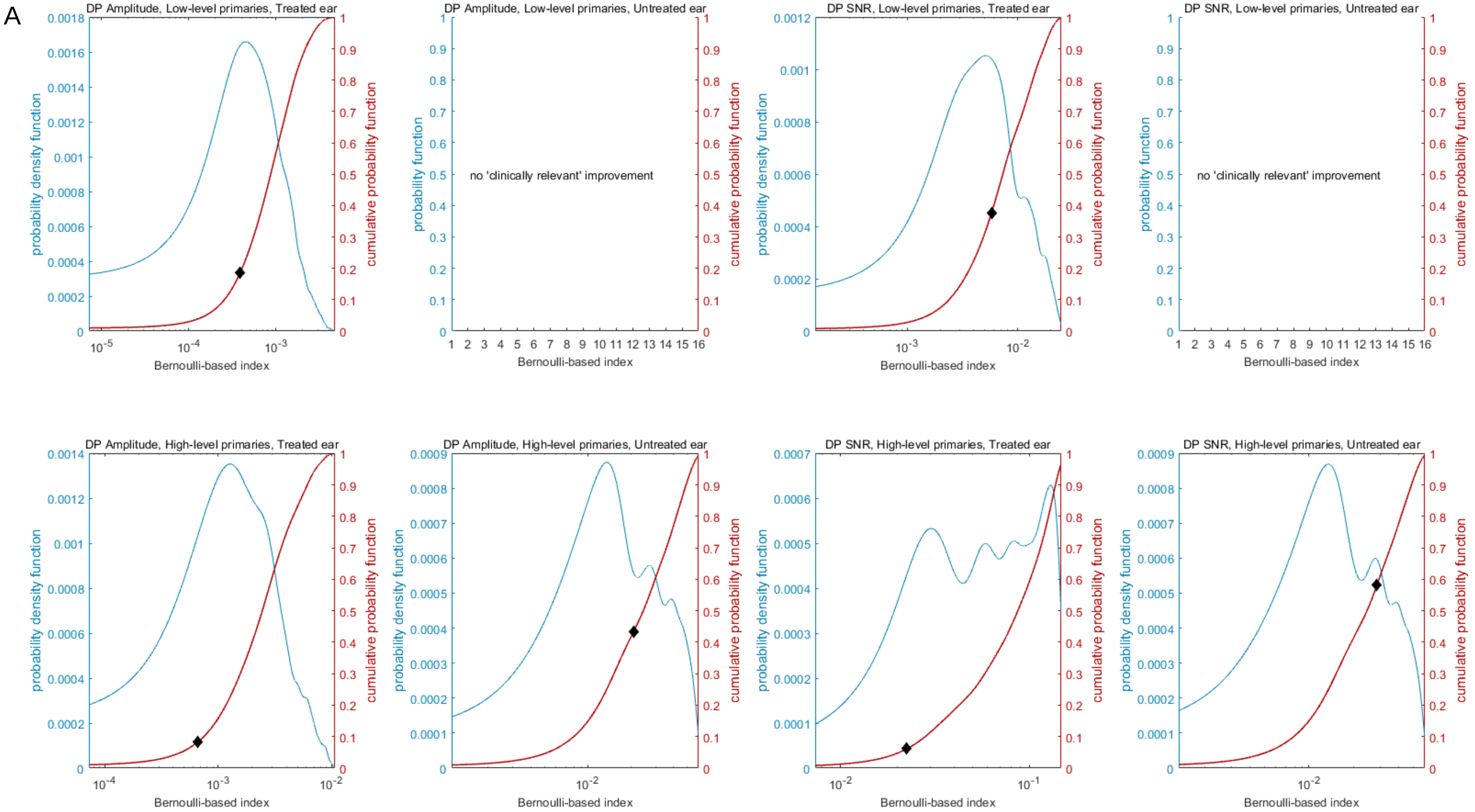

Probability functions $P(p)$ and cumulative distribution functions $P_{cum}(p)$ (red lines) for the Bernoulli-test for randomness of appearance of frequency patterns of “clinically relevant” **improvements.** The diamond markers indicate the $P_{cum}(p_0)$, p_0 =Bernoulli-based index in the pattern.

Figure S6 (B) Bernoulli-test for pattern randomness of ‘clinically relevant DPOAE deterioration

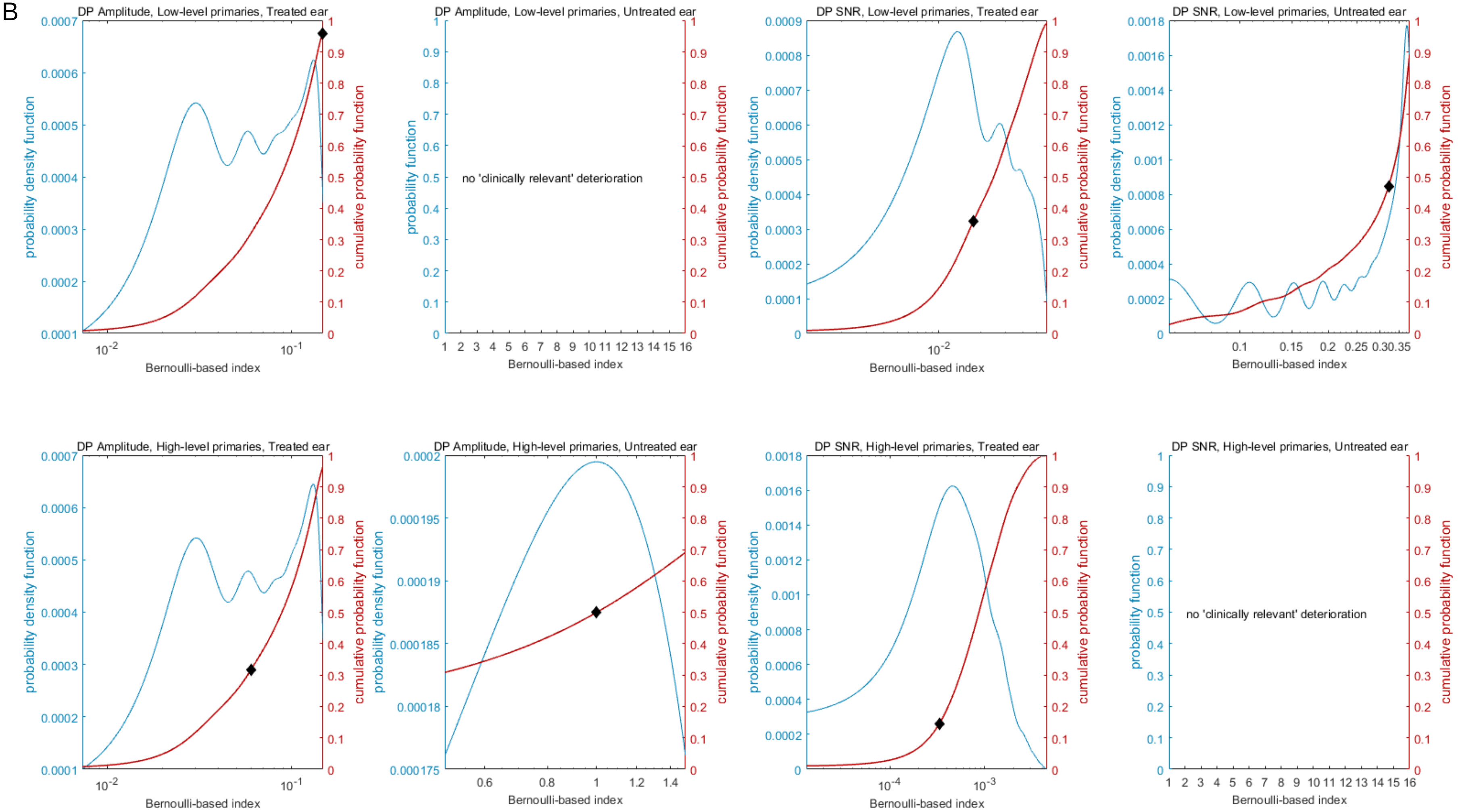

Probability functions $\boldsymbol{P}(\boldsymbol{p})$ and cumulative distribution functions $\boldsymbol{P_{cum}}(\boldsymbol{p})$ (red lines) for the Bernoulli-test for randomness of appearance of frequency patterns of “clinically relevant” **improvements.** The diamond markers indicate the $\boldsymbol{P_{cum}}(\boldsymbol{p_0})$, $\boldsymbol{p_0}$ =Bernoulli-based index in the pattern.

Section 2: Analysis based on DP amplitude data, after adaptation for SNR and signal averaging epochs

In this section, we present the results of the analysis of DPOAE amplitudes after adaptation for SNRs and number of signal averages, according to Attachment 1.

Following the same analysis procedures with the original DP amplitudes data which were reported in the previous section, the post-hoc repeated measures ANCOVA was conducted for each DPOAE frequency on changes in amplitudes from baseline to 6 weeks and 3, 6 and 12 months (namely 6, 12, 24 and 48 weeks), with timepoint, treatment, and timepoint-by-treatment interaction were within-patient factors; trial site as between-patient factor, and baseline values as covariates (both of the treated and untreated ears). Age did not show significant effects, and thus it was removed from the final model. Again, the analysis was conducted separately for low- and high-level tone primaries. The baseline values and the trial site consistently showed larger effects (Table S4).

Figure S6 shows the mean estimated changes from baseline of DP amplitudes, at each frequency for the treated and untreated ear, high-level and low-level primaries and across trial sites and along timepoints, with shaded areas indicating +/- 1.64SE (we relaxed somewhat the 1.96 critical value as the multiplier of SE, in order to ensure that the very few estimated means with confidence intervals which only marginally included 0 (e.g., within ca. 0.1dB) also reach statistical significance, namely the respective CIs were taken as 90% instead of 95%). From these data, the heatmap (Figure S8) depicts for each frequency the average of mean estimated changes from baseline of the

We observe that at low-level primaries, improvements in the treated ear were detected at most frequencies below 1200 Hz, whereas in high-level tone primaries, an improvement in the treated ear may be observed at 4-5kHz and at several frequencies below 1400 Hz. Also, in the untreated ear, improvements are observed for high-level primaries, again mostly below 1500Hz. However, there were also some deteriorations mostly in the untreated ear, in the high-level primaries, and at frequencies >5-6kHz.

Figure S9 shows both the averages and the 95%CIs of the “clinically relevant” changes of Figure S8.

The frequency patterns of “clinically relevant” improvements and deteriorations were tested for randomness using a Wald-Wolfowitz Runs-test (each test with 10million bootstraps), in order to estimate the cumulative probability function of number of runs $\boldsymbol{P_{cum}(r)}$, in the case of random patterns. Figure S9 shows the probability function $\boldsymbol{P(r)}$ and the cumulative distribution function $\boldsymbol{P_{cum}(r)}$;the diamond markers show the $\boldsymbol{P_{cum}(r_0)}$, $\boldsymbol{r_0}$=number of runs in the pattern in each case. These results show that the probability of randomness of appearance of the frequency patterns of “clinically relevant” improvement was lowest in the treated ear, in DPOAE

amplitudes at low-level primaries [$\boldsymbol{P_{cum}}(\boldsymbol{r_0})$=0.04, $\boldsymbol{r_0}$=9], and DPOAE amplitudes [$\boldsymbol{P_{cum}}(\boldsymbol{r_0})$=0.02, $\boldsymbol{r_0}$=9] at high-level primaries. The cumulative probabilities of randomness in case of deteriorations for high-level primaries were [$\boldsymbol{P_{cum}}(\boldsymbol{r_0})$=0.005, $\boldsymbol{r_0}$=4] in the untreated ear, and [$\boldsymbol{P_{cum}}(\boldsymbol{r_0})$=0.19, $\boldsymbol{r_0}$=8] in the treated ear. In all other cases the cumulative probabilities were larger than 0.48.

Using the Bernoulli-based tests (Figure S11) similar results with the Runs-test were obtained for the improvement, namely possibly non-random patterns were identified only at the treated ear and both lo-level and high-level primaries. However, any characteristic deteriorations were only detected in the untreated ear and at high-level primaries.

Table S4 (A) Statistically significant effects of the Repeated Measures ANCOVA, per DPOAE frequency (DP amplitudes at low-level primaries)

A

Frequency (Hz)	Baseline(T)	Baseline(U)	Clinic	Ear(T/U)	Time	Ear(T/U) by Baseline(T)	Ear(T/U) by Baseline(U)	Ear(T/U) by Clinic	Time by Baseline(T)	Time by Baseline(U)	Time by Clinic	Time by Ear(T/U)
498	0.360	0.285	0.361			0.737	0.665					
547	0.478	0.662	0.484			0.762	0.869					
596	0.248	0.152	0.296		0.066	0.619	0.563					
645		0.379	0.250			0.640	0.789					
703	0.534	0.112	0.196			0.840	0.621					
771	0.297	0.179				0.848	0.607					
840		0.365	0.255			0.693	0.742					
918	0.159	0.205	0.230			0.654	0.697					
996	0.193	0.329	0.267			0.715	0.701					
1094	0.444		0.247			0.821	0.573					
1191		0.129	0.152			0.561	0.716					
1299	0.137					0.693	0.563	0.186		0.065		
1416	0.200	0.292	0.159			0.785	0.762					
1543	0.342		0.177			0.770	0.760					
1680	0.139	0.412	0.180			0.730	0.775		0.087			
1836	0.280	0.280	0.371			0.773	0.641					
2002	0.406	0.315	0.353			0.738	0.653					
2178	0.224	0.320	0.320			0.603	0.586					
2383	0.226	0.352	0.433			0.587	0.680					
2598	0.471	0.125	0.322			0.638	0.438					
2832		0.302				0.667	0.491					
3086	0.113	0.577	0.291			0.764	0.851					
3359	0.290	0.639	0.432	0.099		0.788	0.879	0.193	0.094			
3672	0.440	0.578	0.225			0.796	0.814					
4004	0.517		0.354			0.835	0.710	0.193			0.117	
4365		0.288				0.594	0.529				0.110	
4756		0.144				0.372	0.429					
5186	0.632		0.318	0.175		0.884	0.679	0.146			0.135	
5654	0.472	0.460	0.386			0.750	0.783					
6172	0.134	0.295	0.438			0.373	0.547					
6729	0.477	0.376	0.514			0.709	0.588					
7334	0.489	0.499	0.508			0.651	0.603		0.099			
7998	0.171	0.174	0.355			0.585	0.497					
8721	0.155	0.315	0.452			0.674	0.723			0.104		
9512	0.149	0.358	0.448			0.580	0.699					0.088

(A) DP amplitudes at low-level primaries. Effect sizes of statistically significant factors and covariates of the Repeated Measures ANCOVA, per DPOAE frequency ($p \leq 0.05$). Values in cells are the $partial - \eta^2$ values of effect and colors represent categories of effect size (vivid: large, mid: medium, pale: small). All tests were two-sided, without multiple-comparisons correction. After Bonferonni corrections, none of these effect sizes were statistically significant.

Table S4 (B) Statistically significant effects of the Repeated Measures ANCOVA, per DPOAE frequency (DP amplitudes at high-level primaries)

B

Frequency (Hz)	Baseline(T)	Baseline(U)	Clinic	Ear(T/U)	Time	Ear(T/U) *by* Baseline(T)	Ear(T/U) *by* Baseline(U)	Ear(T/U) *by* Clinic	Time *by* Baseline(T)	Time *by* Baseline(U)	Time *by* Clinic	Time *by* Ear(T/U)
498	0.443	0.462	0.303		0.075	0.772	0.731				0.102	
547	0.164	0.437	0.327		0.088	0.790	0.756		0.082			
596		0.537	0.158		0.107	0.474	0.761					0.082
645	0.134	0.250	0.314			0.710	0.760			0.081	0.112	
703	0.149	0.198	0.271			0.681	0.660					
771	0.300	0.119				0.853	0.836					
840		0.556	0.235			0.663	0.846					
918	0.107		0.299			0.527	0.390					
996		0.105	0.239			0.529	0.452					
1094			0.153			0.525	0.553					
1191		0.137	0.146			0.413	0.544					
1299	0.104	0.192	0.255			0.590	0.709					
1416			0.222			0.383	0.338				0.101	
1543		0.123	0.173			0.327	0.321					
1680		0.096	0.186	0.201		0.610	0.638			0.083		
1836		0.525	0.329	0.102		0.630	0.641					
2002	0.112		0.160	0.228		0.593	0.522			0.080		
2178		0.171		0.260		0.701	0.592					
2383	0.145	0.223	0.213			0.397	0.390					
2598	0.441		0.204	0.100		0.624	0.395					
2832	0.314	0.114		0.278		0.610	0.305					
3086	0.221	0.139	0.158	0.159		0.566	0.448				0.110	
3359	0.320	0.380	0.173			0.564	0.725					
3672	0.373			0.110		0.667	0.524					
4004	0.169	0.127				0.495	0.580				0.134	
4365	0.299	0.163				0.745	0.663		0.116		0.103	
4756	0.362					0.825	0.677					
5186		0.253			0.066	0.639	0.614				0.105	
5654	0.228					0.639	0.712					
6172	0.194	0.236				0.632	0.616					
6729	0.216	0.242	0.315			0.615	0.648					
7334	0.492	0.139	0.229		0.078	0.750	0.592					
7998	0.292	0.116	0.258			0.726	0.541					
8721	0.116		0.380			0.641	0.559					
9512	0.129		0.417			0.584	0.653					

(B) DP amplitudes at high-level primaries. Effect sizes of statistically significant factors and covariates of the Repeated Measures ANCOVA, per DPOAE frequency ($p \leq 0.05$). Values in cells are the $partial - \eta^2$ values of effect and colors represent categories of effect size (vivid: large, mid: medium, pale: small). All tests were two-sided, without multiple-comparisons correction. After Bonferonni corrections, none of these effect sizes were statistically significant.

Figure S7 (A) Change from baseline: DPOAE Amplitudes/ Low-level primaries

A

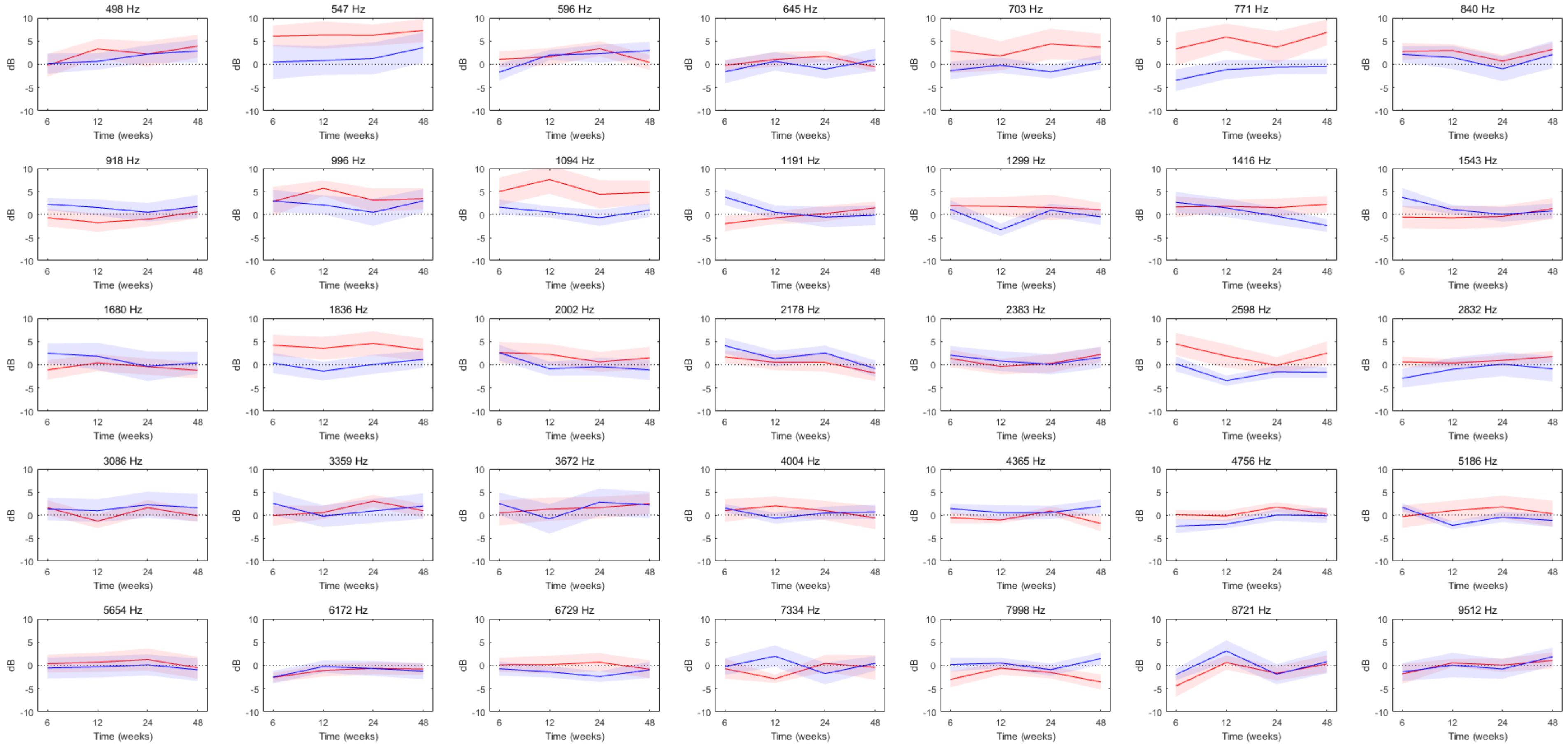

(A) DP amplitudes at low-level primaries. Mean estimated changes (shadings: +/- 2SE) from baseline at each frequency, across trial sites and along timepoints 6 weeks, 3, 6 and 12 months (namely 6, 12, 24 and 48 weeks). Red lines/shadings: treated ear, and blue lines/shadings: untreated ear.

Figure S7 (B) Change from baseline: DPOAE Amplitudes/ High-level primaries

B

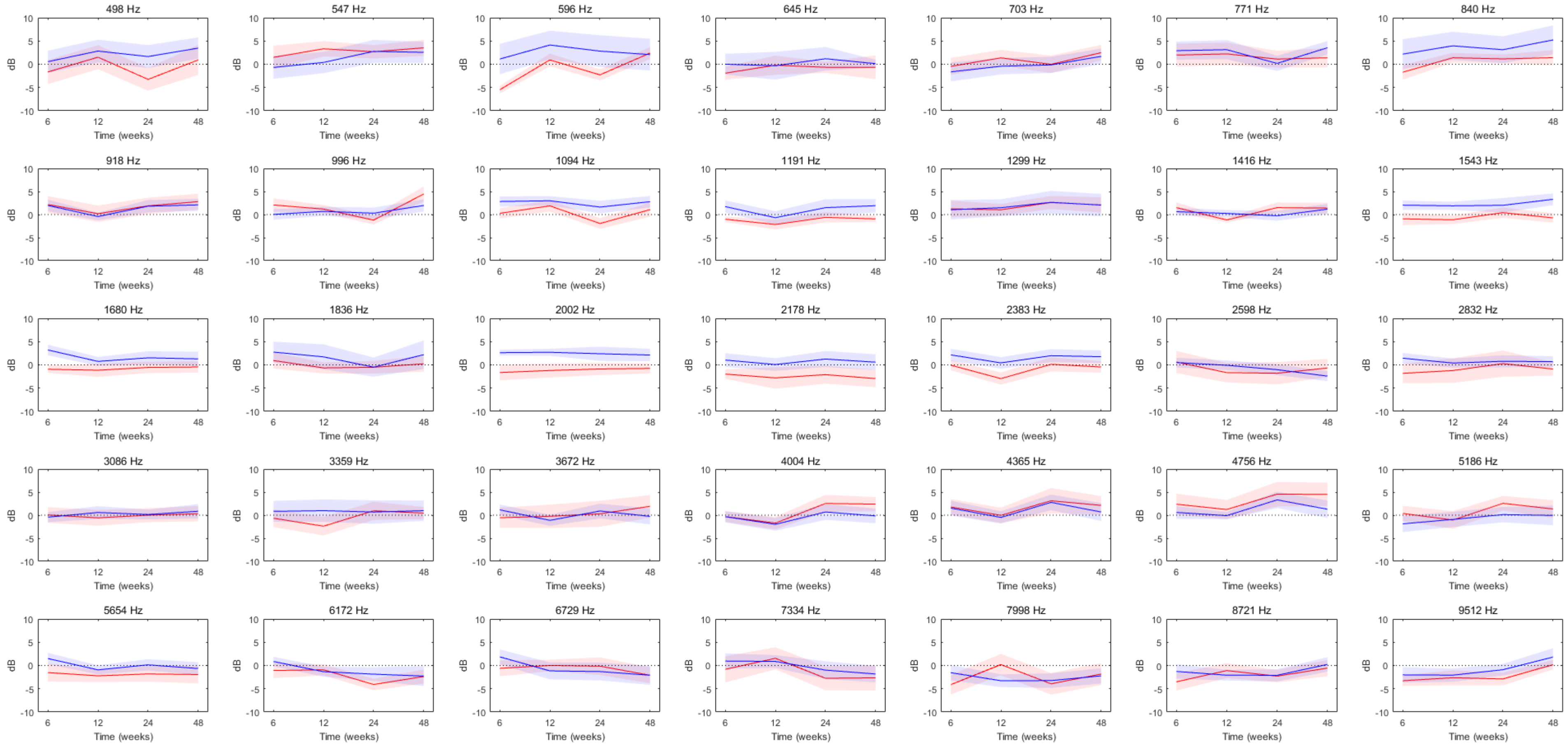

(B) DP amplitudes at high-level primaries. Mean estimated changes (shadings: +/- 2SE) from baseline at each frequency, across trial sites and along timepoints 6 weeks, 3, 6 and 12 months (namely 6, 12, 24 and 48 weeks). Red lines/shadings: treated ear, and blue lines/shadings: untreated ear.

Figure S8 “Clinically relevant” changes of the DP amplitudes and SNRs

Graded heatmap and average values of “clinically relevant” changes of the DP amplitudes and SNRs (improvements shown in green, deteriorations in red).

	Low-level primaries		High-level primaries	
	Amplitude		Amplitude	
Frequency (Hz)	Treated ear	Untreated ear	Treated ear	Untreated ear
498		2.5		
547	6.5		3.2	2.7
596		2.4		
645				
703	4.0			
771	5.4			3.3
840	2.8		1.3	4.1
918			2.3	2.0
996	4.8	2.5	1.6	
1094	5.4			2.6
1191			-1.6	
1299			2.3	
1416			1.5	
1543		2.4		2.3
1680				
1836	3.9			
2002	2.4			2.5
2178			-2.5	
2383				1.9
2598		-2.2		
2832				
3086				
3359				
3672				
4004			2.5	
4365			2.6	
4756		-2.2	4.5	
5186				
5654			-2.0	
6172			-3.2	-1.8
6729		-1.9		
7334				
7998	-2.6			-2.9
8721				-2.1
9512			-2.9	-2.5

Figure S9: (A) ‘clinically relevant’ DPOAE improvement, (B) ‘clinically relevant’ DPOAE deterioration

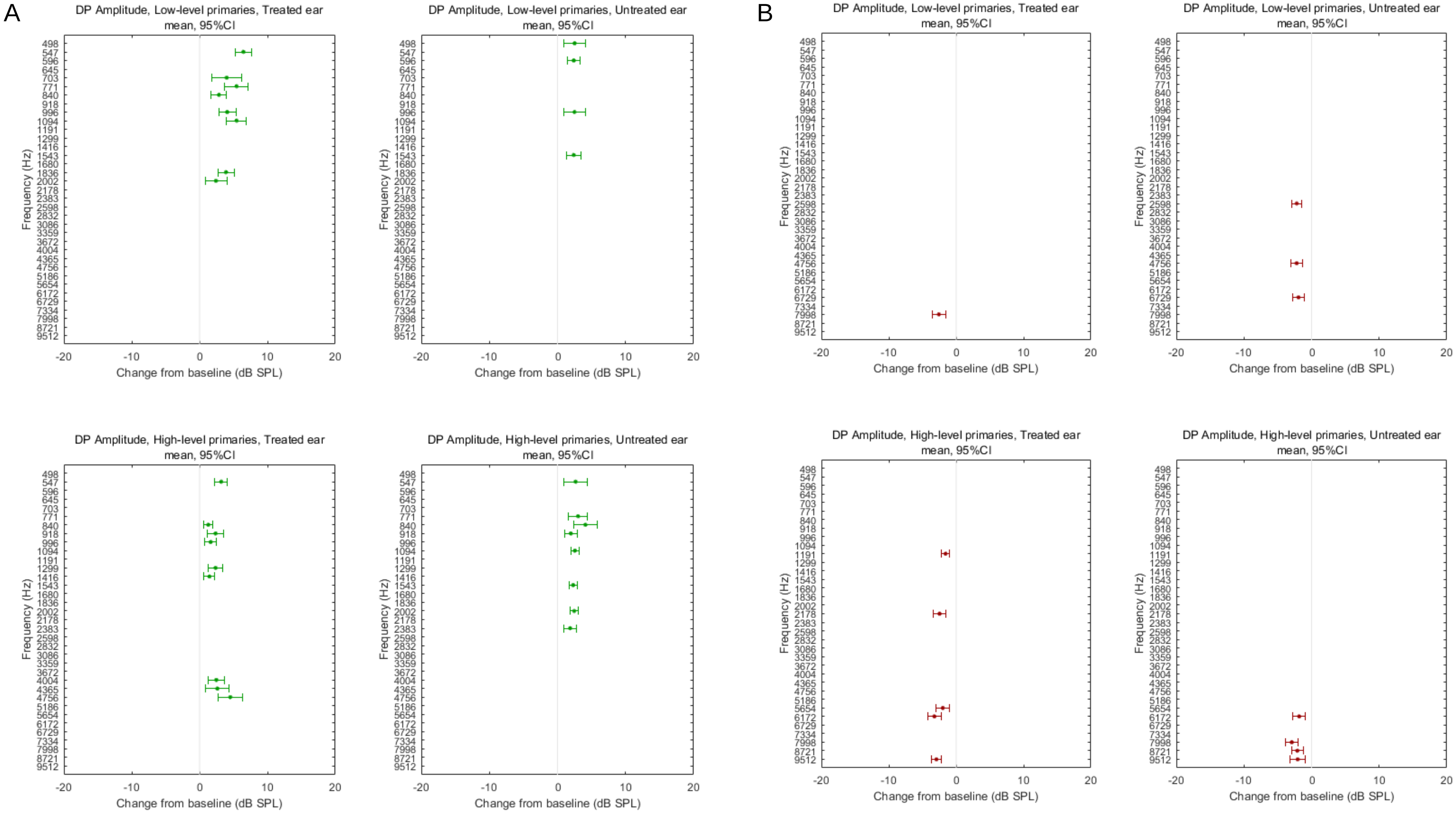

Average and 95%CI of “clinically relevant” deteriorations for DP amplitudes, low-level and high-level primaries and treated/untreated ear.

Figure S10: Runs-test for pattern randomness of (A) ‘clinically relevant DPOAE improvements, (B) ‘clinically relevant DPOAE deterioration

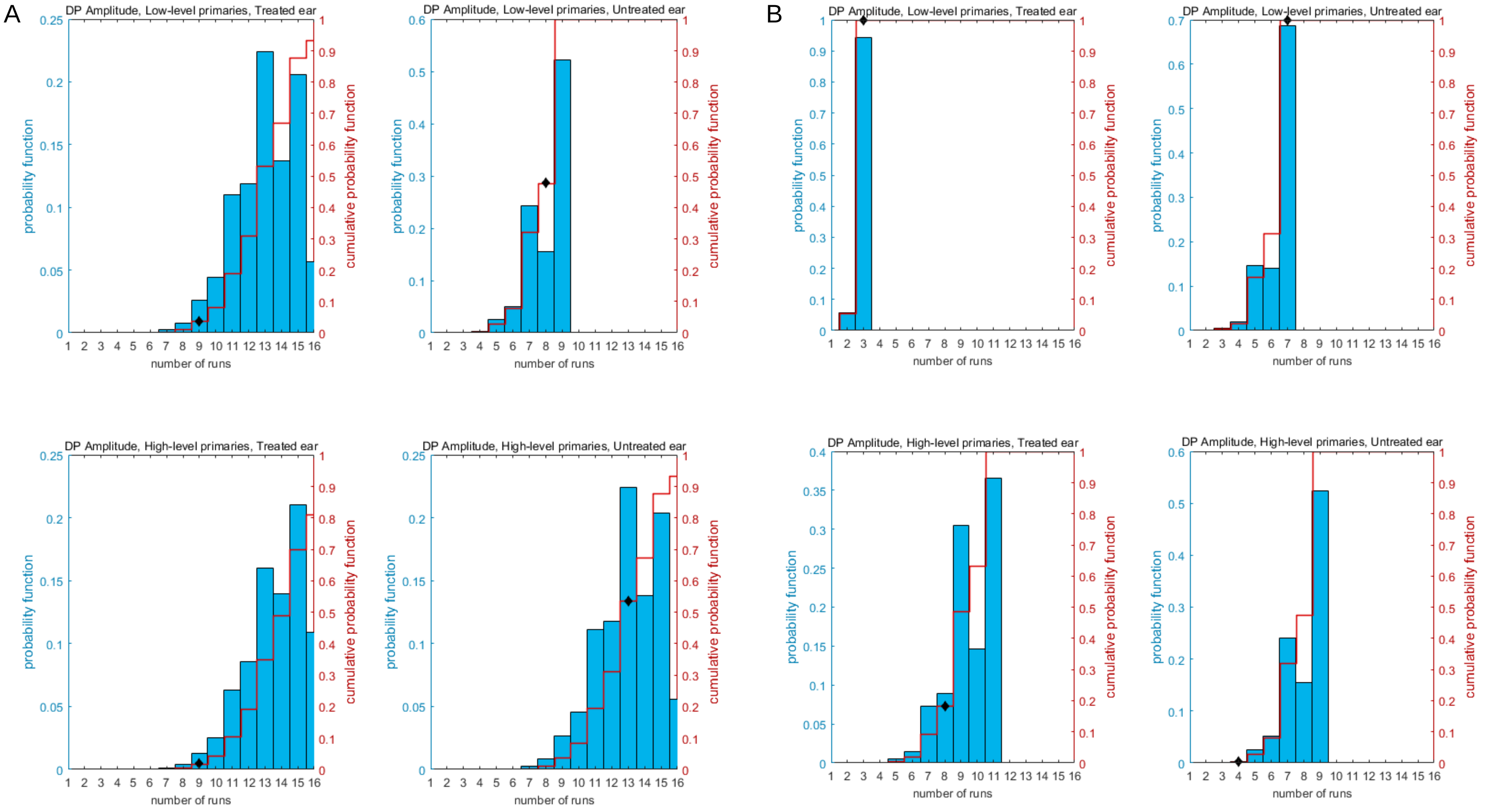

Probability functions $\boldsymbol{P}(\boldsymbol{r})$ and cumulative distribution functions $\boldsymbol{P}_{\boldsymbol{cum}}(\boldsymbol{r})$ (red lines) for the Wald-Wolfowitz Runs-test for randomness of appearance of frequency patterns of “clinically relevant” **improvements** and **deteriorations.** The diamond markers mark the $\boldsymbol{P}_{\boldsymbol{cum}}(\boldsymbol{r}_0)$, $\boldsymbol{r}_0$=number of runs in the pattern.

Figure S11 (A) Bernoulli-test for pattern randomness of ‘clinically relevant DPOAE improvement

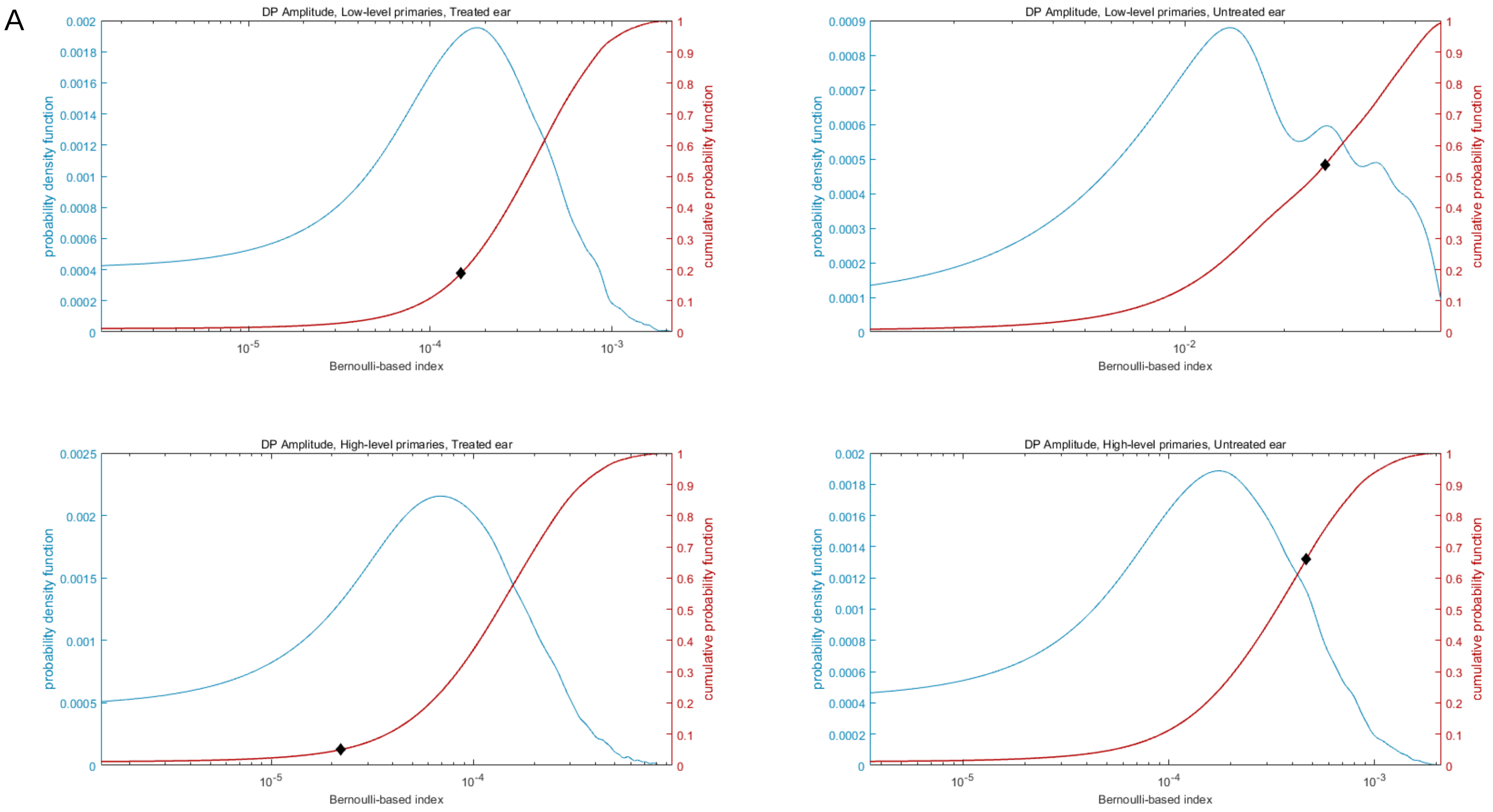

Probability functions $\boldsymbol{P}(\boldsymbol{p})$ and cumulative distribution functions $\boldsymbol{P}_{cum}(\boldsymbol{p})$ (red lines) for the Bernoulli-test for randomness of appearance of frequency patterns of “clinically relevant” **improvements.** The diamond markers indicate the $\boldsymbol{P}_{cum}(\boldsymbol{p}_0)$, $\boldsymbol{p}_0$ =Bernoulli-based index in the pattern.

Figure S11 (B) Bernoulli-test for pattern randomness of ‘clinically relevant DPOAE deterioration

B

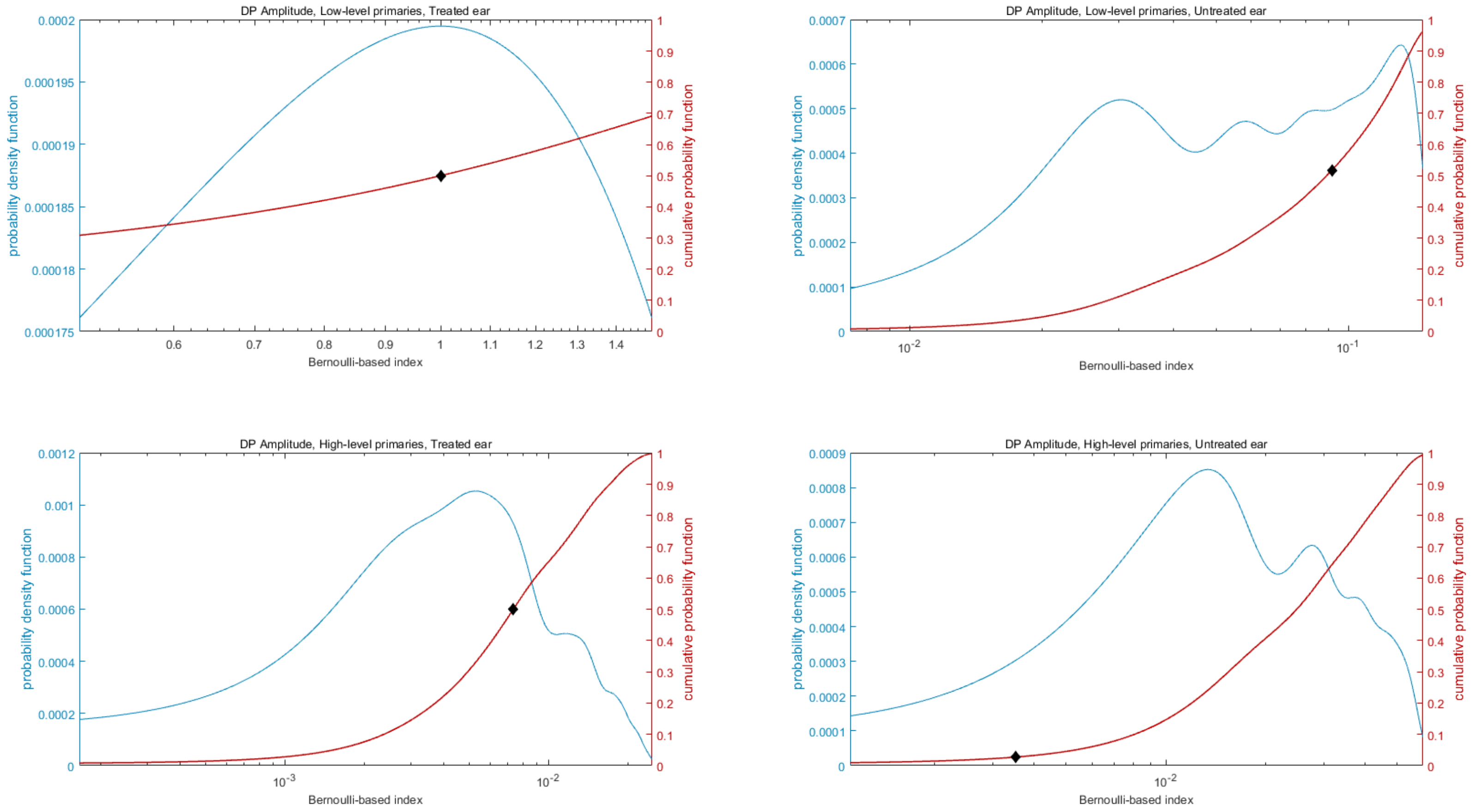

Probability functions $\boldsymbol{P}(\boldsymbol{p})$ and cumulative distribution functions $\boldsymbol{P}_{\boldsymbol{cum}}(\boldsymbol{p})$ (red lines) for the Bernoulli-test for randomness of appearance of frequency patterns of “clinically relevant” **improvements.** The diamond markers indicate the $\boldsymbol{P}_{\boldsymbol{cum}}(\boldsymbol{p}_0)$, $\boldsymbol{p}_0$ =Bernoulli-based index in the pattern

Comparative assessment of findings of Section 1 and Section 2

In the following Tables S5 and S6 we summarize the findings of the previous sections, regarding clinically-relevant improvements/deteriorations and randomness of frequency patterns, for both the original DPOAE amplitude data and the estimates of DPOAE amplitude after adaptation for SNR and averaging epochs. There is good agreement between the analyses of both data sets.

Table S5: Findings for clinically-relevant improvements/deteriorations

**Improvements**				
	Low-level primaries		High-level primaries	
	Treated ear	Untreated ear	Treated ear	Untreated ear
Original DP amplitudes	<1200Hz	-	4-5kHz	-
Adapted DP amplitudes	<1200Hz	-	<1500Hz<br>4-5kHz	<1500Hz
**Deteriorations**				
	Low-level primaries		High-level primaries	
	Treated ear	Untreated ear	Treated ear	Untreated ear
Original DP amplitudes	-	-	-	-
Adapted DP amplitudes	-	-	>5-6kHz	>5-6kHz

The – symbol indicates that there is no clear effect

Clinically-relevant improvements are observed at low-level primaries and for frequencies <1200Hz in the treated ear, and at high-level primaries for frequencies 4-5kHz in the treated ear. Improvements are also detected at high-level primaries for frequencies <1500Hz, in both the treated and untreated ears, however, only with the adapted data.
Clinically-relevant deteriorations are observed only at high-level primaries for frequencies >5-6kHz, in both the treated and untreated ears, however, only with the adapted data.
In the rest of the conditions, the patterns of improvements/deteriorations are sparse and thus no clearly observable effect can be identified.

Table S6: Test for non-randomness of patterns pf clinically relevant improvements/deteriorations

**Improvements**				
	Low-level primaries		High-level primaries	
	Treated ear	Untreated ear	Treated ear	Untreated ear
**Original DP amplitudes**				
Runs-test	+	-	+	-
Bernoulli-based test	+	-	+	-
**Adapted DP amplitudes**				
Runs-test	+	-	+	-

Bernoulli-based test	+	-	+	-
**Deteriorations**				
	Low-level primaries		High-level primaries	
	Treated ear	Untreated ear	Treated ear	Untreated ear
**Original DP amplitudes**				
Runs-test	-	-	-	-
Bernoulli-based test	-	-	-	-
**Adapted DP amplitudes**				
Runs-test	-	-	+	+
Bernoulli-based test	-	-	-	+

The + symbol indicates potentially non-random pattern, whereas the – symbol indicates random pattern

We selected values of cumulative density functions <0.3 as potentially indicative of non-random frequency patterns of distributions of clinically-relevant improvements/deteriorations. Non-random patterns of improvement are detected only in the treated ear at both low-level and high-level primaries, and for both datasets (original and adapted amplitudes) and types of statistical test (Wald-Wolfowitz and Bernoulli-based tests).
On the other hand, non-random patterns of deteriorations are only detected at the untreated ear, at high-level primaries, and only with the adapted amplitudes dataset. In the treated ear, potentially non-random deteriorations were detected only by the Wald-Wolfowitz Runs-test and the adapted amplitudes dataset.

Attachment 1: DPOAE amplitude estimates (adapted for SNR and signal averaging epochs)

We employ a data processing technique for the estimation of the “true” level of the DPOAEs, which takes into account the SNR value and the number of trials spent during the averaging process of DP recordings (which aims the reduction of recorded noise) and allows for a correction of the reported DPOAE amplitude level, especially at lower SNRs.

Although there exist several processing protocols for the recorded signal (Dillier, 2005), (Shaffer & Dhar, 2006), (Dalhoff et al., 2013) an essential part of them is some form of time-domain averaging of signal frames (Kemp, 2009), (Gorga et al., 1994), (Janssen & Müller, 2008) which are retained form the total number of recorded trials after application of some initial criterion (e.g. total level of frame (Madsen et al., 2009), signal or noise indexes/statistics (Siedlecki & Zielinski, 2015), (Long et al., 2008), (Shaffer & Dhar, 2006), (Janssen & Müller, 2008), (Giebel, 2001), etc.)

With the time-domain averaging process in mind, at a specific DP frequency, we assume that the recorded signal is $y_{DP}(n) = DP(n) + w(n)$, where $DP(n)$ is a (quasi) deterministic Distortion-Product OAE signal. $w(n)$ is a Gaussian, ergodic, zero-mean ($\mu_w = 0$) additive band-pass noise (around the specific DP frequency) with variance σ_w^2 (Pa), which is uncorrelated/incoherent with $DP(n)$ (Long et al., 2008), namely $w \sim \boldsymbol{N}(0, \sigma_w^2)$.

Then, we define the following quantities:
$\widehat{L_{true}}$: estimate of the "true" amplitude level (dB SPL) of DPOAE signal $DP(n)$
L_{noise}: the noise floor level (dB SPL)
L_{app}: the estimated amplitude (dB SPL) of DPOAE, which is reported after the time-domain averaging of the noisy recordings $y_{DP}(n)$. It holds that $\widehat{L_{true}} \leq L_{app}$ (since L_{app} "contains" also a contribution from residual noise (Giebel, 2001), (Dhar et al., 1998), (Backus, 2007))
$SNR_{app} = L_{app} - L_{noise}$: the estimated (and reported) SNR (dB SPL)
M: the retained number of trials during the time-domain averaging of the noisy recordings. The median value of M in the study was 30.
It is known that during the time-domain averaging, the contribution of noise in the estimated amplitude of the DPOAE is reduced by $10\log_{10}(M)$ dB.

It can be shown (also see (Dhar et al., 1998) and (Backus, 2007)) that:

$$L_{app} = 10\log_{10}\left(10^{\frac{\widehat{L_{true}}}{10}} + 10^{\frac{L_{noise}-10\log_{10}M}{10}}\right) \quad (1)$$

Thus, we maintain:

$$\left.\begin{array}{r} L_{app} = 10\log_{10}\left(10^{\frac{\widehat{L_{true}}}{10}} + 10^{\frac{L_{noise}-10\log_{10}(M)}{10}}\right) \\ SNR_{app} = L_{app} - L_{noise} \end{array}\right\} \Rightarrow \left.\begin{array}{r} \dots \\ L_{noise} = L_{app} - SNR_{app} \end{array}\right\} \Rightarrow$$

$$\Rightarrow 10^{\frac{\widehat{L_{true}}}{10}} = 10^{\frac{L_{app}}{10}} - 10^{\frac{L_{app}-SNR_{app}-10\log_{10}(M)}{10}} \Rightarrow$$

$$\Rightarrow \widehat{L_{true}} = 10\log_{10}\left(10^{\frac{L_{app}}{10}} - 10^{\frac{L_{app}-SNR_{app}-10\log_{10}(M)}{10}}\right) \quad (2)$$

In the rare case $SNR_{app} \leq -10\log_{10}(M)$ it holds that:

$$\left.\begin{array}{r} SNR_{app} = L_{app} - L_{noise} \\ \widehat{L_{true}} \leq L_{app} \end{array}\right\} \Rightarrow$$

$$\Rightarrow \left.\begin{array}{r} SNR_{app} + L_{noise} = L_{app} \\ \widehat{L_{true}} \leq L_{app} \end{array}\right\} \xrightarrow{SNR_{app} \leq -10\log_{10}(M)} \left.\begin{array}{r} L_{app} \leq L_{noise} - 10\log_{10}(M) \\ \widehat{L_{true}} \leq L_{app} \end{array}\right\} \Rightarrow$$

$$\Rightarrow \left.\begin{array}{r} \widehat{L_{true}} \leq L_{app} - SNR_{app} - 10\log_{10}(M) \\ \dots \end{array}\right\} \Rightarrow$$

$$\Rightarrow \widehat{L_{true}} \leq L_{app} - SNR_{app} - 10\log_{10}(M) \quad (3)$$

As $-\left(SNR_{app} + 10\log_{10}(M)\right) \geq 0$, we can choose for $\widehat{L_{true}} - L_{app}$ any value less than $-\left(SNR_{app} + 10\log_{10}(M)\right)$. We arbitrarily choose a value $\widehat{L_{true}} = L_{app} - SNR_{app} - 10\log_{10}(M) - 3$.
The number of cases for which this condition holds is very small (0.2% of total cases), and thus we expect that the above choice (although arbitrary) will not have any significant erroneous impact to the final results.

Attachment 2: Bernoulli-based test for randomness of the distribution along frequency of clinically important outcomes

A test for randomness of the pattern of clinically important outcomes at the various DP frequencies may be constructed by considering these outcomes as a sequence of independent Bernoulli trials (Papoulis, 2002), with $p = N_{pos}/N_{total}$, where N_{pos} is the number of frequencies at which clinically important outcomes are observed, and N_{total} is the total number of frequencies in the testing series (in our case N_{total}=35).

Thus, starting from the first frequency with clinically important outcome, each following similar outcome is considered a success trial in a sequence of intermediate failed trials. In this way, and since the trials are independent, the probability each such subsequence of consecutive failures and one success is computed by a Binomial distribution (Papoulis, 2002), namely $Pr\left(1; N_{sub}^{(i)}, p\right) = \binom{N_{sub}^{(i)}}{1} p(1-p)^{N_{sub}-1}$, where $N_{sub}^{(i)}$ is the length of the ith subsequence. The overall probability of the pattern is computed as the product of these probabilities, namely $Pr_{pattern}\left(1; N_{sub}^{(i)}, p\right) = \prod_i Pr\left(1; N_{sub}^{(i)}, p\right)$.

The test is actually computing the probability density function and cumulative density function of the values of $\mathrm{Pr}_{pattern}^{random}\left(1; N_{sub}^{(i)}, p\right)$ for truly random patterns using a Monte-Carlo simulation (10000 runs) with N_{pos} and N_{total}, and estimates the value of $p \leq Pr_{pattern}\left(1; N_{sub}^{(i)}, p\right)$ for the examined pattern of clinically important outcomes.

References

Backus, B. C. (2007). Bias due to noise in otoacoustic emission measurements. *The Journal of the Acoustical Society of America*, *121*(3), 1588–1603. https://doi.org/10.1121/1.2434831

Dalhoff, E., Turcanu, D., Vetešník, A., & Gummer, A. W. (2013). Two-source interference as the major reason for auditory-threshold estimation error based on DPOAE input–output functions in normal-hearing subjects. *Hearing Research*, *296*, 67–82. https://doi.org/10.1016/j.heares.2012.12.003

Dhar, S., & Hall III, J. W. (2011). *Otoacoustic Emissions: Principles, Procedures, and Protocols* (1st edition). Plural Publishing, Inc.

Dhar, S., Long, G. R., & Culpepper, N. B. (1998). The Dependence of the Distortion Product 2 $f_1 - f_2$ on Primary Levels in Non-Impaired Human Ears. *Journal of Speech, Language, and Hearing Research*, *41*(6), 1307–1318. https://doi.org/10.1044/jslhr.4106.1307

Dillier, N. (2005). *Otoacoustic emissions* [Lecture]. Medical Acoustics, Department of Otorhinolaryngology, Head and Neck Surgery at the University Hospital, University of Zurich.

Giebel, A. (2001). Applying signal statistical analysis to TEOAE measurements. *Scandinavian Audiology. Supplementum*, *30*, 130–132. https://doi.org/10.1080/010503901300007308

Gorga, M. P., Neely, S. T., Bergman, B. M., Beauchaine, K. L., Kaminski, J. R., & Liu, Z. (1994). Towards understanding the limits of distortion product otoacoustic emission measurements. *The Journal of the Acoustical Society of America*, *96*(3), 1494–1500. https://doi.org/10.1121/1.410227

Interacoustics. (2019). *A Guide to Otoacoustic Emissions* (Interacoustics a/s-8515061-2-03/2019).

Janssen, T., & Müller, J. (2008). Otoacoustic Emissions as a Diagnostic Tool in a Clinical Context. In G. A. Manley, R. R. Fay, & A. N. Popper (Eds.), *Active Processes and Otoacoustic Emissions in Hearing* (pp. 421–460). Springer. https://doi.org/10.1007/978-0-387-71469-1_13

Kemp, D. T. (2009). Otoacoustic Emissions. In L. R. Squire (Ed.), *Encyclopedia of Neuroscience* (pp. 317–326). Academic Press. https://doi.org/10.1016/B978-008045046-9.00265-5

Long, G. R., Talmadge, C. L., & Lee, J. (2008). Measuring distortion product otoacoustic emissions using continuously sweeping primaries. *The Journal of the Acoustical Society of America*, *124*(3), 1613–1626. https://doi.org/10.1121/1.2949505

Madsen, E., Kochendorfer, F. C., Roldan Ramirez, S., & Cofino Corcoles, J. (2009). *Matlab based GUI for DPOAE Signal Estimation*. Aalborg University, Acoustics Department of Electronic Systems.

Papoulis, A. (2002). *Probability, Random Variables and Stochastic Processes* (4th edition). McGraw-Hill Europe.

Reavis, K. M., McMillan, G., Austin, D., Gallun, F., Fausti, S. A., Gordon, J. S., Helt, W. J., & Konrad-Martin, D. (2011). Distortion-Product Otoacoustic Emission Test Performance for Ototoxicity Monitoring. *Ear & Hearing*, *32*(1), 61–74. https://doi.org/10.1097/AUD.0b013e3181e8b6a7

Shaffer, L. A., & Dhar, S. (2006). DPOAE Component Estimates and Their Relationship to Hearing Thresholds. *Journal of the American Academy of Audiology*, *17*(04), 279–292. https://doi.org/10.3766/jaaa.17.4.6

Siedlecki, T., & Zielinski, J. (2015). SPECTRAL KURTOSIS OF OTOACOUSTIC EMISSIONS USING THE HUSIMI TRANSFORM: A PILOT STUDY. *Journal of Hearing Science*, *5*(4), 15–25. https://doi.org/10.17430/896155

Zelle, D., Dalhoff, E., & Gummer, A. W. (2017). Time-domain analysis of distortion-product otoacoustic emissions using a hydrodynamic cochlea model. *Current Directions in Biomedical Engineering*, *3*(2), 453–456. https://doi.org/10.1515/cdbme-2017-0095
