# Supplementary material for: A phase I/IIa safety and efficacy trial of intratympanic gamma-secretase inhibitor as a regenerative drug treatment for sensorineural hearing loss
Source: Nat Commun. 2024 Mar 1;15:1896. doi: 10.1038/s41467-024-45784-0 (PMC10907343; doi:10.1038/s41467-024-45784-0)
Supplement: Supplementary file 3 — Description of Additional Supplementary Files [file 41467_2024_45784_MOESM3_ESM.pdf]

Description of Additional Supplementary Files

Supplementary Data 1: Trial protocol version 1.0 (22 March 2017)

Supplementary Data 2: Trial protocol version 5.0 (04 December 2018)

Supplementary Data 3: Statistical Analysis Plan (SAP)
