# Supplementary material for: A phase I/IIa safety and efficacy trial of intratympanic gamma-secretase inhibitor as a regenerative drug treatment for sensorineural hearing loss
Source: Nat Commun. 2024 Mar 1;15:1896. doi: 10.1038/s41467-024-45784-0 (PMC10907343; doi:10.1038/s41467-024-45784-0)
Supplement: Supplementary file 5 — Supplementary Data 2 [file 41467_2024_45784_MOESM5_ESM.pdf]

RESEARCH PROTOCOL

REGAIN

A Phase I/II multiple ascending dose open-label safety and efficacy study of the Notch Inhibitor LY3056480 in patients with mild to moderate SNHL.

(Version 5.0 – 04 December 2018)

Protocol title

A Phase I/II multiple ascending dose open-label safety and efficacy study of the Notch Inhibitor LY3056480 in patients with mild to moderate sensorineural hearing loss.

**Protocol ID**	AUT-001
**Short title**	REGAIN
**EudraCT number**	2016-004544-10
**Version**	Final version 5.0
**Date**	04 December 2018
**Clinical Phase**	II (part B)
**Coordinating investigator**	Prof Anne Schilder<br>NIHR Research Professor, Professor of Paediatric Otorhinolaryngology & Director evidENT, UCL
**Principal investigators**	Prof Shakeel Saeed, Professor of Otology and Neuro-Otology, UCL and Consultant ENT and Skullbase Surgeon & Divisional Clinical Director, RNTNEH<br>Stephan Wolpert, University of Tübingen, Universitäts klinikum Tübingen<br>Athanasios Bibas, CMath MSc (Audiol) PhD FRCSI (Otol), Assistant Professor and Consultant Surgeon at the University of Athens
**Sponsor**	Audion Therapeutics BV<br>Linnaeusparkweg 10-2<br>1098 EA, Amsterdam<br>Netherlands
**Subsidising party**	Horizon2020

PROTOCOL SIGNATURE SHEET - SPONSOR

Name	Signature	Date
**Sponsor or legal representative:**<br>Rolf Jan Rutten, CEO<br>Audion Therapeutics	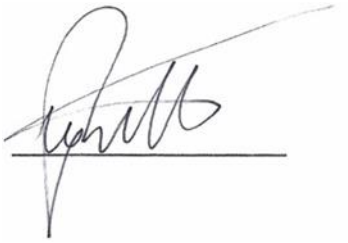	04 December 2018
**Coordinating Investigator:**<br>Prof Anne Schilder<br>University College London	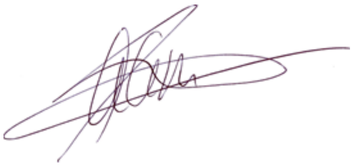	04 December 2018

SIGNATURE SHEET – PRINCIPAL INVESTIGATOR

Title: A Phase I/II multiple ascending dose open-label safety and efficacy study of the Notch Inhibitor LY3056480 in patients with mild to moderate sensorineural hearing loss (SNHL).

This study protocol was subjected to critical review. The information it contains is consistent with current knowledge of the risks and benefits of the investigational medicinal product, as well as with the moral, ethical and scientific principles governing clinical research as set out in the guidelines on Good Clinical Practice and the declaration of Helsinki.

All documentation for this study that is supplied to me and that has not been previously published will be kept in the strictest confidence. This documentation includes this study protocol, Investigator's Brochure, Case Report Forms, and other scientific data.

The study will not be commenced without the prior written approval of a properly constituted Competent Authority (CA) or Independent Ethics Committee (IEC). No changes will be made to the study protocol without the prior written approval of the Sponsor and the CA or IEC, except where necessary to eliminate an immediate hazard to the subjects.

I have read and understood and agree to abide by all the conditions and instructions contained in this protocol.

**Name**	**Signature**	**Date**
**Principal Investigator:**		

TABLE OF CONTENTS

1. INTRODUCTION AND RATIONALE 11
   - 1.1 Types and causes of hearing loss 11
   - 1.2 Current treatment options for sensorineural hearing loss 12
   - 1.3 Hair cell regeneration: Atoh1 is a master switch 12
   - 1.4 Atoh1 depends on Notch signalling 13
   - 1.5 Gamma-secretase inhibitors (GSI's) and hair cell regeneration 13
   - 1.6 Summary of findings from non-clinical studies leading to LY3056480 selection 16
   - 1.7 Relevant clinical studies 17
     - 1.7.1 Atoh1 17
     - 1.7.2 Safety profile of GSIs and LY3056480 17
     - 1.7.3 Systemic versus topical drug delivery 18
     - 1.7.4 Inner ear hair cell loss and SNHL in human patients 18
   - 1.8 Summary 19

2 SUMMARY OF REGAIN PART A TRIAL (CONCLUDED NOVEMBER 2018) 20

3 STUDY OBJECTIVES and ENDPOINTS OF REGAIN PART B TRIAL (Phase II) 21
   - 3.1 Objectives 21

4 STUDY DESIGN 22
   - 4.1 Location and procedures 22
   - 4.2 Dosage 22
   - 4.3 Endpoints 22
   - 4.4 Justification of study design 23

5 STUDY POPULATION 25
   - 5.1 Population 25
   - 5.2 Inclusion criteria 25
   - 5.3 Exclusion criteria 25
   - 5.4 Lifestyle guidelines 26

6 TREATMENT OF PARTICIPANTS 28
   - 6.1 Investigational product LY3056480 28

7 INVESTIGATIONAL PRODUCT 29
   - 7.1 Name and description of investigational product 29
   - 7.2 Summary of findings from non-clinical studies 30
   - 7.3 Summary of findings from clinical studies 30
   - 7.4 Summary of known and potential risks and benefits 30
   - 7.5 Dosages, dosage modifications and method of administration 30
   - 7.6 Efficacy study 30
   - 7.7 Preparation and labelling of Investigational Medicinal Product 30
   - 7.8 Drug accountability 31

8 METHODS 32
   - 8.1 Blinding 32
   - 8.2 Study procedures 32

8.2.1 Patient Identification ... 32
8.2.2 Informed consent ... 32
8.2.3 Screening ... 32
8.2.4 Treatment – first dose (Day 1 / Baseline) ... 33
8.2.5 Follow-up ... 33
8.2.6 Assessments ... 33
8.3 Definition of end of trial ... 37
8.4 Withdrawal of individual participants ... 37
8.5 Replacement of individual participants after withdrawal ... 37
8.6 Follow-up of participants withdrawn from treatment ... 38
8.7 Premature termination of the study/stopping rules ... 38
9 SAFETY REPORTING ... 39
9.1 Temporary halt for reasons of participant safety ... 39
9.2 Definitions ... 39
9.3 Recording Adverse Events ... 40
9.4 Assessment of Adverse Events ... 40
9.4.1 Severity ... 40
9.4.2 Causality ... 41
9.4.3 Expectedness ... 42
9.4.4 Seriousness ... 42
9.5 Abnormal test findings ... 42
9.6 AEs, SAEs and SUSARs ... 43
9.6.1 Adverse Events ... 43
9.6.2 Recording and Reporting Serious Adverse Events ... 43
9.6.3 Suspected unexpected serious adverse reactions (SUSARs) ... 43
9.7 Annual safety report ... 43
9.8 Follow-up of Adverse Events ... 44
9.9 Data Safety Monitoring Board ... 44
10 STATISTICAL METHODOLOGY AND ANALYSIS ... 45
10.1 Statistical analysis plan ... 45
10.2 Statistical power calculations ... 45
10.3 Analysis sets ... 45
10.4 Missing data ... 46
10.5 Summary statistics ... 46
10.6 Analysis of primary endpoint ... 46
10.7 Analysis of secondary endpoints ... 47
10.8 Analysis of safety endpoints ... 48
10.9 Analysis of further endpoints ... 48
10.10 Interim analysis (if applicable) ... 48
11 ETHICAL CONSIDERATIONS ... 49
11.1 Regulation statement ... 49
11.2 Recruitment and consent ... 49
11.3 Benefits and risks assessment, group relatedness ... 49

11.4 Compensation for injury 49
11.5 Incentives 50
12 ADMINISTRATIVE ASPECTS, MONITORING AND PUBLICATION 51
12.1 Handling and storage of data and documents 51
12.2 Monitoring and Quality Assurance 51
12.3 Amendments 52
12.4 Annual progress report 53
12.5 Temporary halt and (prematurely) end of study report 53
12.6 Public disclosure and publication policy 53
13 REFERENCES 55

LIST OF ABBREVIATIONS AND RELEVANT DEFINITIONS

ADL	Activities of Daily Living	IMP	Investigational Medicinal Product
AE	Adverse Event	(m)ITT	(Modified) Intention-to-Treat
AL	Audiologist	IWRS	Interactive Web-based Randomization System
ALT (SGPT)	Alanine Aminotransferase	kHz	Kilohertz
AR	Adverse Reaction	LDH	Lactate Dehydrogenase
ART	Acoustic Reflex Test	L/HDL Cholesterol	Low/High Density Liposome Cholesterol
AST (SGOT)	Aspartate Aminotransferase	MedDRA	Medical Directory for Regulatory Activities
Atoh1/Hath1	Atonal Homolog 1	MHRA	Medicines and Healthcare Products Regulatory Agency
AzD	Alzheimer's disease	MM	Medical Monitor
BUN	Blood Urea Nitrogen	MTD	Maximum Tolerated Dose
CA	Competent Authority	NBCD	Nordic Bioscience Clinical Development
Ca++	Calcium	NICD	Notch Intracellular Domain
(CB)CT	(Cone Beam) Computed Tomography	NOAEL	No-Observed-Adverse-Effect Level
CI	Coordinating Investigator	NSAID	Non-Steroidal Anti-Inflammatory Drug
CK	Creatine Kinase	PD	Pharmacodynamics
CRA	Monitor/Clinical Research Associate	PI	Principal Investigator
(e)CRF	(Electronic) Case Report Form	PK	Pharmacokinetic
CRP	C-Reactive Protein	PTA	Pure-Tone Audiometry
CTCAE	Common Terminology Criteria for Adverse Events	QoL	Quality of Life
dB	Decibel	RBC	Red Blood-Cell Count
dBHL	Decibels Hearing Level	RN	Research Nurse
DLT	Dose-Limiting Toxicity	RNTNEH	Royal National Throat, Nose and Ear Hospital – UCL Hospital
DPOAE	Distortion Product Oto-Acoustic Emissions	RWM	Round Window Membrane
DSMB	Data Safety Monitoring Board	SAE	Serious Adverse Event
ECG	Electrocardiogram	SAP	Statistical Analysis Plan
EDC	Electronic Data Capture	SAR	(Unexpected) Serious Adverse Reaction
ENT	Ear, Nose and Throat	SD	Standard Deviation
ESR	Erythrocyte Sedimentation Rate	SNHL	Sensorineural Hearing Loss
EudraCT	European drug regulatory affairs Clinical Trials	SNR	Signal to Noise Ratio
GCP	Good Clinical Practice	SOP	Standard Operating Procedure
GGT	Gamma-GT	Sponsor	See definition in Section 1.53 of ICH-GCP
GI	Gastrointestinal	SUSAR	Suspected Unexpected Serious Adverse Reaction
GS	Gamma-Secretase	TEAE	Treatment-emergent adverse events
GSI	Gamma-Secretase Inhibitor	TEN	Threshold-Equalizing-Noise
HbA1C	Hemoglobin A1c	TFI	Tinnitus Function Test
HCT	Haematocrit	UCL(H)	University College London (Hospital)
Hes1/Hes5	Hairy and Enhancer of Split-1/5	UK	United Kingdom
Hgb	Haemoglobin	US	United States
IB	Investigator's Brochure	VNG	Videonystagmography
ICH	International Conference on Harmonisation	WBC	White Blood-Cell Count
IEC	Independent Ethics Committee		

SUMMARY

Rationale:

LY3056480 may induce trans-differentiation of supporting cells into inner-ear hair cells and lead to a subsequent improvement of hearing in patients with sensorineural hearing loss (SNHL). The biological mechanism through which this occurs has been studied, the efficacy of this class of molecules was studied in animal models, and LY3056480 was tested in various *in vitro* and *ex vivo* models for hair cell regeneration. The product is locally applied which offers the opportunity to reach the target organ while exposing a subject to limited quantities of the investigational medicinal product (IMP).

In a First in Man study (REGAIN – part A), the safety and tolerability of LY3056480 in patients with NSHL was investigated. The 250µg dose was found to be a safe dose to continue to the proof of concept trial to study efficacy, part B of the REGAIN trial.

Objectives:

Objectives REGAIN - Part B
1. Main objective is to establish the efficacy of local treatment with LY3056480 in terms of hearing at 12 weeks;
2. To establish the efficacy of local treatment with LY3056480 in terms of hearing at 6 weeks;
3. To assess the safety and tolerance of local treatment with LY3056480.

Study design:

Part B is a multi-centre efficacy phase II study with one dose of LY3056480. This Part is designed to establish efficacy parameters at 250 µg of LY3056480.

Study population:

Adult volunteers with mild to moderate (25 to 60 decibels Hearing Level (dBHL)) SNHL of a 5-frequency pure-tone average (0.5 kHz, 1 kHz, 2 kHz, 4 kHz and 8 kHz) will be recruited through Adult Audiology Services in the United Kingdom (UK), Germany and Greece.

Key Inclusion Criteria
1. Male or female between 18 and 80 years of age;
2. A primary complaint of hearing loss of ≤20 years in duration, the history suggesting this hearing-loss to be of age-related, noise-induced or idiopathic origin;
3. A bilateral, symmetrical (<15 dBHL difference) SNHL with a pure-tone average threshold across the frequencies 0.5, 1, 2, 4 and 8 kHz of between 25 and 60 dBHL with 2 or more frequencies less than 60 dBHL.

Key Exclusion Criteria
1. Presenting with a primary complaint of tinnitus;

2. A ‘true’ air-bone gap ≥15 dBHL in 3 or more contiguous frequencies between 0.5, 1, 2, 4 kHz;
3. History of suspected or diagnosed genetic cause of hearing loss;
4. Suspected or known diagnosis of inner ear pathology, congenital hearing loss, fluctuating hearing loss, Ménière’s disease, or secondary endolymphatic hydrops, perilymph fistula, cochlear barotrauma, autoimmune hearing loss, radiation-induced hearing loss, retro-cochlear lesion;
5. Evidence of acute or chronic otitis media or otitis externa on examination; or a history of middle ear pathology and/or surgery;
6. Any therapy known as ototoxic within 12 months of screening.

Intervention:

Three injections of LY3056480 administered trans-tympanically into one ear.
The participants will receive the highest tolerable dose determined in Part A of the trial; 250µg of LY3056480 applied in 500 µL.

Main study parameters/endpoints:

The primary efficacy endpoint of REGAIN - Part B is:
Average change in hearing from baseline in the treated ear at 12 weeks across three frequencies (2, 4, 8 kHz), as measured by Pure Tone Audiometry (PTA) (dBHL).
The secondary efficacy endpoints are a change in hearing, balance and tinnitus.
Safety and tolerability endpoints are changes in hearing, balance and facial nerve function and the occurrence and severity of treatment and procedure related local and systemic AEs.

Nature and extent of the burden and risks associated with participation, benefit and group relatedness:

Participants in the current trial will be given the opportunity to receive a new local treatment with promising results from pre-clinical studies, to potentially reverse the SNHL. The potential to obtain treatment benefit is considered to be the main benefit of participation in the trial.
As the treatment is applied locally at a relatively low dose, the risks of systemic toxicity are considered very low. As described in the Investigators Brochure (Section 5), there was no toxicity in a dog study that involved twice weekly oral dosing (Study 8001611).
There are risks associated with the treatment administration procedure, and risks associated with the unintended pharmacological activity of the IMP in the inner ear, middle ear or in the proximity of the middle ear. The treatment procedure (trans-tympanic injection) is a well-known method of drug delivery to the inner ear, with known risks, and the risks associated with the trans-tympanic injections are evaluated as acceptable when taking the potential benefit into consideration. In the First in Man trial the IMP was found to be safe and tolerable.

1. INTRODUCTION AND RATIONALE

1.1 Types and causes of hearing loss

There are different types of hearing loss and a variety of causes. Hearing loss is either due to insufficient sound conduction from the outer to the inner ear ("conductive hearing loss") or to damage to the hair cells or auditory neurons in the cochlea ("sensorineural hearing loss") in over 90% of cases (Hong, Kerr, Poling, & Dhar, 2013). Hearing loss can occur acutely but is more often a progressive process.

Sensorineural hearing loss (SNHL) is the most common form of hearing loss and is most often due to damage or death of the hair cells that are the sensory receptor cells in the cochlea located in the inner ear (see right Figure 1 and Figure 2). Another type of hair cells (type 1 and type 2) responsible for sensory motion and spatial orientation are located in the vestibular system. Coiling on the inside of the cochlea is the organ of Corti that contains the cells responsible for hearing, the hair cells. The number of human auditory hair cells is relatively low (about 15,000 per cochlea at birth). Two types of hair cells have been identified: Inner and outer hair cells. Hair cells have stereocilia or "hairs" that stick out. The bottom of hair cells are attached to the basilar membrane, and the stereocilia are in contact with the tectorial membrane. Inside the cochlea, sound waves cause the basilar membrane to vibrate. The vibration triggers a shearing force between the basilar and tectorial membranes, causing the hair cell stereocilia to bend back and forth and leading to internal changes within the hair cells that create electrical signals. Auditory nerve fibres rest below the hair cells and pass these signals on to the brain.

Figure 1. Anatomy of the ear

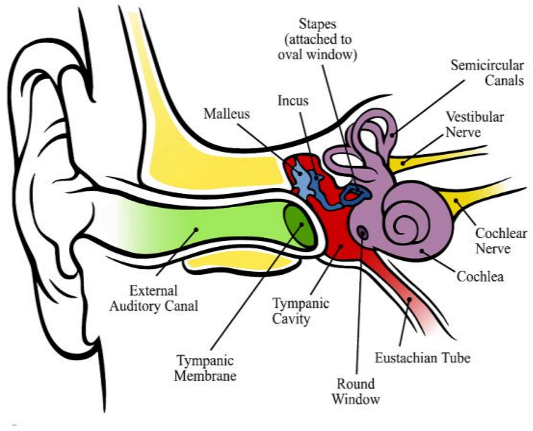

Figure 2. The Organ of Corti

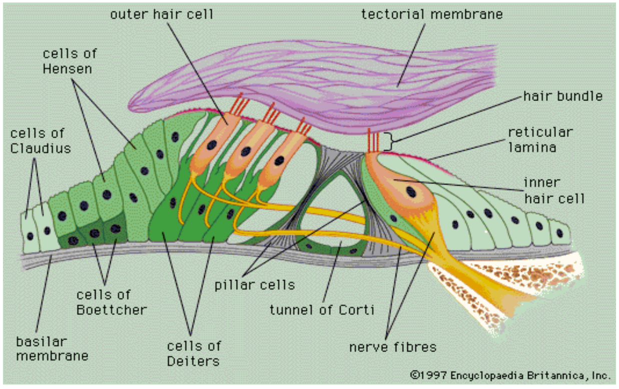

Once the cells in a particular area of the cochlea are lost, hearing at a particular frequency is compromised. In a high percentage of cases, it is the injury or loss of auditory hair cells (SNHL) which causes hearing loss. This injury or loss is mostly caused by age, exposure to noise and certain ototoxic drugs. Inner ear hair cells are not replaced after loss or damage in adult mammals (including human) (Edge & Chen, 2008; Müller & Barr-Gillespie, 2015), in contrast to lower vertebrates like birds, reptiles and fish (Rubel, Furrer, & Stone, 2013). For this reason, restoring hearing after hair cell loss has long been assumed not to be possible.

1.2 Current treatment options for sensorineural hearing loss

At this time, no small molecule therapeutics for hearing loss are on the market. The treatment of SNHL is currently limited to the use of hearing aids or cochlear implants. These instruments do not treat the underlying physiological cause of the hearing impairment. Hearing aids only amplify sounds and do not improve the intrinsic capacity to hear. They tend to work even less effectively when a larger number of hair cells has been lost and often perform poorly in noisy environments, and their uptake is disappointingly low (about 15-20%, (Knudsen, Oberg, Nielsen, Naylor, & Kramer, 2010; Lin 2012; McCormack & Fortnum, 2013). Cochlear implants bypass the hair cells and directly stimulate the auditory nerve. Cochlear implants are very costly (assessment, implantation, intensive support and maintenance for the first three years is estimated to cost $100,000 (Vio & Holme, 2005). Moreover, the procedure to implant the device is invasive and may negatively affect any residual hearing. Therefore, they are only used for a minority of people with profound hearing loss. Besides, cochlear implants are limited in their ability to reproduce the full range of sound frequencies that we hear and convey pitch, i.e. the transformation of tones and sounds. Therefore, small molecule drugs safely targeting the underlying biological causes of hearing loss will address a medical need that affects millions of patients. The approach using an inhibitor of the Notch pathway (see below), LY3056480 has the potential to add a therapeutic option to the limited options currently available.

1.3 Hair cell regeneration: Atoh1 is a master switch

In lower vertebrates, new hair cells are generated from progenitor cells that surround hair cells. These cells, called supporting cells, have the capacity to differentiate into hair cells (Stone & Rubel, 2000). Such progenitor cells are also found in the human cochlea. During embryonic development, the basic helix-loop-helix transcription factor named atonal homolog 1, or Atoh1, is both necessary and sufficient for hair cell differentiation of the mammalian inner ear. Due to this crucial role, Atoh1 is a prime candidate target for therapeutic approaches to stimulate hair cell regeneration.

This important role for Atoh1 was further validated by:

. (i) Knock-out mice of the murine equivalent of Atoh1 fail to generate hair cells (Bermingham et al., 1999);

. (ii) In an *ex vivo* cochlea, introduction of the murine Atoh1 induced formation of hair cells from non-differentiated epithelial cells (Zheng & Gao, 2000);

. (iii) In a deafened animal with damaged hair cells, expression of murine Atoh1 induced auditory hair cell formation and improved hearing (Izumikawa et al., 2005);

. (iv) *In utero* gene transfer of Atoh1 in mice resulted in the production of supernumerary hair cells in the cochlea (Gubbels, Woessner, Mitchell, Ricci, & Brigande, 2008).

1.4 Atoh1 depends on Notch signalling

While Atoh1 promotes supporting cells to turn into hair cells, proteins in the Notch signalling pathway act to block hair cell regeneration. Notch signalling is a cell-cell communication system. Signalling through the Notch receptor is responsible for a process called lateral inhibition, which is active during development of the auditory epithelium to ensure that the correct number and pattern of hair cells and supporting cells are attained. Notch accomplishes this task through a series of molecular interactions, including the activation of transcription factors that inhibit Atoh1 expression. Consequently, Notch inhibition upregulates Atoh1 (Daudet & Lewis, 2005; Kelley 2006).

Figure 3. Notch pathway in inner ear cells

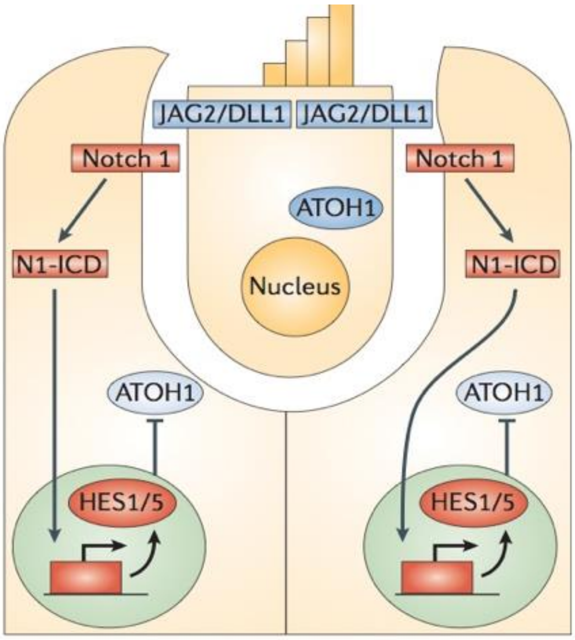

Notch receptors are expressed on the surface of developing supporting cells, and Notch binding proteins are expressed on the surface of developing hair cells. When a Notch binding protein binds to the Notch receptor, a part of the Notch receptor (called a Notch intracellular domain or NICD) is cleaved by an enzyme called gamma-secretase. The cleaved NICD translocates to the nucleus where it activates inhibitory transcription factors (e.g. HES1 and HES5) and blocks Atoh1 expression in the supporting cell. This prevents the supporting cell from committing to becoming a hair cell (see right Figure 3). Thus, blocking Notch signalling induces differentiation of supporting cells into hair cells. Mechanistic work on the role of Notch confirmed the requirement for Atoh1 for the efficacy of Notch inhibition, as preventing Atoh1 expression at the time of inhibitor treatment blocked the differentiation to hair cells (Jeon, Fujioka, Kim, & Edge, 2011). Small molecule compounds that inhibit or block Notch signalling are potential agents to promote hair cell regeneration and auditory recovery in mammals (Mizutari et al., 2013).

1.5 Gamma-secretase inhibitors (GSI's) and hair cell regeneration

Gamma-secretase (GS) is a multi-protein complex responsible for cleavage of Notch receptors, amyloid precursor protein, and others. GS is required for the first step of Notch signal transduction; the cleavage of the precursor form of Notch and subsequent activation of downstream genes activated by NICD. Therefore, inhibition of GS results in inhibition of Notch signalling**.** As inhibitors of Notch signalling, GS inhibitors (GSIs) promote hair cell differentiation from inner ear stem cells through upregulation of Atoh1. See Table 1 for an overview of the various studies with GSIs demonstrating hair cell regeneration in rodent cochlear tissue or from otic progenitor or stem cells. (Bramhall, Shi, Arnold, Hochedlinger, & Edge, 2014; Hori et al., 2007; Jeon et al., 2011; Mizutari et al., 2013; Tona et al., 2014; Yamamoto et al., 2006; Zhao et al., 2011).

Table 1. Select GSI studies demonstrating regeneration of inner ear hair cells

GSI studies demonstrating regeneration of inner ear hair cells	Species	Year	Authors
Inhibition of Notch/RBP-J signalling induces hair cell formation in neonate mouse cochleas	Mice	2006	Yamamoto et al.
Inhibition of Notch signalling with GSI and hair cell formation in mature guinea pig cochlea	Guinea pigs	2007	Hori et al.
DAPT a low potency, specificity GSI induces Hair Cell Production in Cultured Organ of Corti from Neonatal Rats	Rat	2011	Zhao et al.
In vitro evidence for hair cell formation from otic progenitors	Mice	2011	Jeon et al.
First *in vivo* evidence in adult mice for Hair cell regeneration and partial hearing recovery	Mice	2013	Mizutari et al.
Second *in vivo* evidence in adult guinea pigs for Hair cell regeneration and partial hearing recovery	Guinea pigs	2014	Tona et al.
Lineage tracing study showing Lgr5 positive supporting cells are target for GSI based Notch inhibition and transdifferentiation to new hair cells	Mice	2014	Bramhall et al.

In 2013 Audion's scientific collaborators generated non-clinical evidence that local middle ear delivery of a GSI (LY411575) to inhibit Notch signalling can be employed to regenerate lost outer hair cells *in vivo* in a noise induced hearing loss mouse model. This regeneration resulted in a small yet significant hearing recovery in midrange frequencies. This work shows validation of the potential of GSI's in regenerating hair cells and the functional restoration of hearing on three levels:

1. In vitro evidence in cell-based assays and Organ of Corti explant models

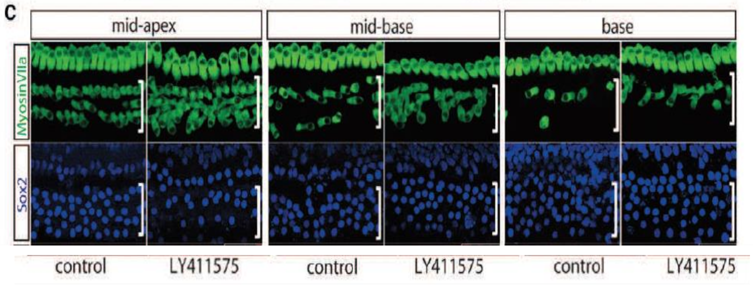

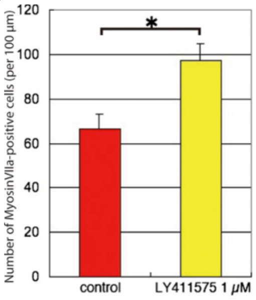

Figure 4 (left) and 5 (right). Figure 4 shows the effects of a small molecule Notch inhibitor triggering new hair cell growth after induced hair cell loss. Myosin VIIa is a hair cell marker for mature hair cells, Sox2 a supporting cell marker. The treated explants revealed an increased number of myosin VIIa-positive cells in the outer hair cell region (white bracket) compared to the carrier-treated explants across different regions of the cochlea. Moreover, they show a reduced number of Sox2 positive supporting cells which is evidence for trans-differentiation of supporting cells (Mizutari et al., 2013). Quantification per 100 µm of the cultured organ of Corti explants from P1 mice at 72 hours is shown in Figure 5 (2013).

2. Mechanistic evidence

In the same 2013 study, it was demonstrated that Hes5 expression (a downstream effector gene of Notch that inhibits Atoh1 gene expression) is suppressed and that Atoh1 expression levels are increased after treatment with a Notch inhibitor. With lineage tracing the study also showed that new hair cells were the result of trans-differentiation of supporting cells to new hair cells.

3. In vivo effects in adult mice

Local trans-tympanic administration of the GSI (LY411575) in mice with acoustic trauma, resulted in the formation of new outer hair cells and resulted in partial recovery of hearing as measured by Auditory Brainstem Responses. Even using relatively low doses, this effect was detectable after one week and lasted for at least three months (longest time measured). Local administration of GSIs as compared to systemic administration mitigates systemic toxicity, and no side effects were observed in this study. Figure 6 shows that administration of the GSI LY411575 increases the number of outer hair cells *in vivo*.

Figure 6. Myosin VIIa is a marker for hair cells and Sox2 for supporting cells

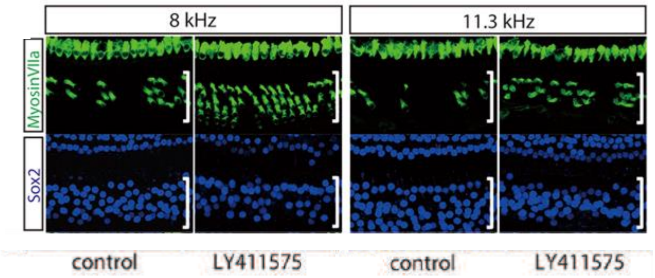

It is clear that after treatment outer hair cell numbers have increased while supporting cells have decreased. This was further illustrated when these cells were quantified across frequencies. The significant differences in the numbers of hair cells and supporting cells that were observed in the outer hair cell area at 8 and 11.3kHz regions after three months between treated and untreated ears is illustrated in Figure 7.

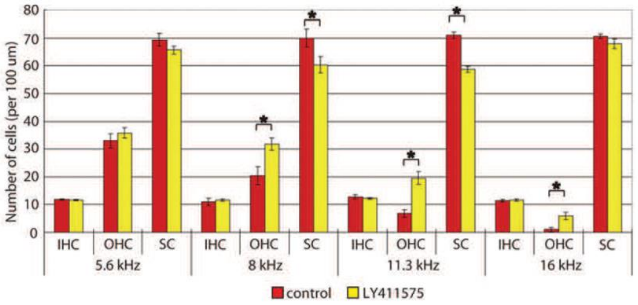

Figure 7. Significant differences in the numbers of hair cells and supporting cells in the outer hair cell area of treated (left) ears three months after treatment with LY411575 as compared to the values in the contralateral carrier-treated ear of deafened mice. Figures 4, 5, 6 and 7 are adapted from (2013).

A number of *in vitro* and *ex vivo* studies published by our collaborators and other independent groups generated additional data that support the results published in Neuron (2013). A 2014 study independently confirmed that pharmacological inhibition of Notch signalling with a different, less potent notch inhibitor, MDL28170, and in a different noise-induced hearing loss animal model (using guinea pigs instead of mice as a species) also induced outer hair cell regeneration as well as hearing recovery. In this study, direct cochlear delivery using a micro-osmotic pump instead of trans-tympanic delivery was used (Tona et al., 2014).

Bramhall et al. demonstrated (using a mouse model) that the supporting cells, which surround hair cells in the normal cochlear epithelium, are the target cells of GSI mediated inhibition of Notch and differentiate into new hair cells in the neonatal mouse following ototoxic damage (Bramhall et al., 2014). Lineage tracing experiments in this study showed that predominantly outer hair cells arise from differentiating Lgr5-positive otic progenitors cells.

1.6 Summary of findings from non-clinical studies leading to LY3056480 selection

The body of work summarised above, and in particular the *in vivo* hearing recovery results, as well as the underlying mechanistic work which proves the role of Notch inhibition by LY411575, form the rationale for the development program of LY3056480 in SNHL.

LY3056480 was selected by Audion from a set of newer generation GSIs generated by Eli Lilly. The aim was to choose a potent, yet safe molecule with characteristics that allow development into a drug product for local delivery to the human middle ear. The *in vitro* LY3056480 pharmacology studies, as well as inner ear PK studies and all non-clinical safety studies, are summarised in the LY3056480's Investigator's Brochure (IB).

Audion's inner ear pharmacology assays are largely similar to the methods used by (Mizutari et al., 2013) though they are optimised further for use in the program. They were also extended with a human intestinal cell line model measuring Atoh1 upregulation upon Notch inhibition by LY3056480. These studies provide both human activity data, as well as Whole Cochlea neonatal mouse model data supported by confocal imaging of explants (see Figure 8) after treatment with LY3056480.

LY3056480 is active with acceptable potency in a dose-dependent fashion in all cellular assays. Treatment of human intestinal cell line and all mouse neonatal primary tissue models including the organ of Corti and whole cochlea explants results in responses indicative for molecular mechanisms that could lead to trans-differentiation of supporting cells to new outer hair cells. The non-clinical inner ear PK studies showed that concentrations that are expected to efficiently inhibit Notch can be obtained in the inner ear. Detailed information is available in the current version of the IB.

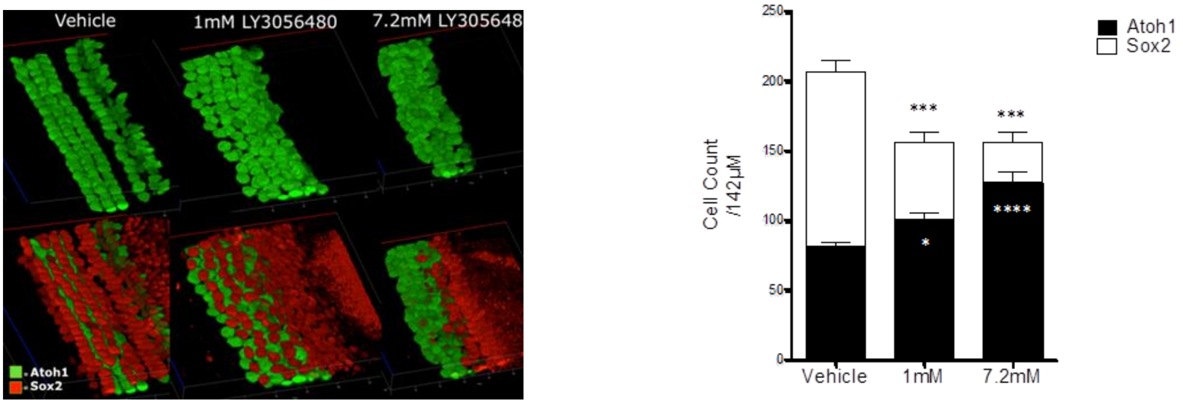

Figure 8. Dual-immunocytochemistry and quantification with confocal analysis demonstrates significant effects of LY3056480 (1mM and 7.2mM formulated in 10% Poloxamer338) on two cell types (Atoh1-positive hair cells and Sox2-positive supporting cells) in the sensory epithelia of whole cochlea cultures. Graph shows LY3056480 increases in Atoh1-positive hair cell density averaged through the all regions of the Organ of Corti (black bars), which is contrasted by decreased supporting cell number (underlying white bars) in the same regions. Dose-dependent increases in hair cell density (Atoh1-positive nuclei, green) are observed with LY3056480 at 1mM and 7.2mM. In contrast, fewer visible supporting cell (Sox2-positive nuclei, red) are observed with increasing drug treatment. These data are consistent with Notch1 inhibition of supporting cells by LY3056480 leads to gene expression changes involved in the transdifferentiation to hair cells. (N=3; *, P<0.05).

1.7 Relevant clinical studies

To date, a single First in Man phase I trial has been conducted treating 15 patients with mild to moderate NSHL with three doses. In this dosing escalation trial (REGAIN part A), no SAEs were reported, and no safety signals were identified. An independent DSMB concluded that the 250µg dose level was tolerable and considered safe to use in part B of REGAIN.

In the paragraph below, further relevant clinical data supporting our approach have been outlined. This outline consists of; a trial targeting Atoh1 upregulation, the clinical safety profile of GSI's, the systemic versus topical delivery, and the translation of pre-clinical data to human.

1.7.1 Atoh1

The concept of upregulating the expression of Atoh1 to induce regeneration of hair cells is currently being tested by Novartis using a locally administered adenoviral vector named CGF166. This gene therapy trial is being conducted in the United States (US). CGF166 is a recombinant adenovirus-5 vector carrying the human version of Atoh1, Hath1, cDNA for administration via intra-labyrinthine infusion.

1.7.2 Safety profile of GSIs and LY3056480

GSIs were originally developed to block the production of amyloid-beta peptide, or Aβ, for the treatment of Alzheimer's disease (AzD) (De Strooper, Vassar, & Golde, 2010). Several molecules have been used in clinical studies for AzD through daily systemic treatment (up to Phase III, 2-year daily administration) (Doody et al., 2013; Panza et al., 2010; Siemers et al., 2006). The fact that most GSIs also inhibit Notch signalling in other tissues, such as the gastrointestinal (GI) tract and T-lymphocytes, has been an obstacle in the systemic, high-dose, chronic, daily use for AzD. When given systemically in high doses and on a daily basis, the most common dose-limiting toxicology is the overproduction of mucus leading to (GI) effects (Henley, Sundell, Sethuraman, Dowsett, & May, 2014; Siemers et al., 2005). This side-effect is in line with the pharmacology since Notch inhibition drives precursor cells toward a secretory cell fate in the GI.

The effects of chronic use are exemplified in the safety data of a Phase III AzD study published in 2013 for semagacestat (Doody et al., 2013). The adverse event rate in this placebo-controlled study was at least twice as high in the combined active treatment groups than in the

placebo group: e.g. basal cell and squamous cell skin cancers, gastrointestinal symptoms such as decreased appetite, vomiting, and weight loss, skin rashes, and more (Henley et al., 2014). Early termination of the semagacestat treatment due to AEs, led to futility in most of the efficacy parameters, when compared to placebo in this study.

First, second and third generation inhibitors have been developed and have been used in clinical studies not only for AzD, but most recently the next generation compounds are also studied for Notch-dependent malignancies (Table 2).

Table 2. Completed clinical trials of GSIs in cancer (Yuan et al., 2015).

Treatment	Trial type and tumor	Enrollment	Primary endpoint	Therapy effect
MK0752	Phase I; advanced solid tumor	103	MTD	1 CR and 12 SD
MK0752	Phase I; normal	30	Notch response signature	NA
MK0752 + docetaxel	Phase I/II; breast cancer	30	DLT	11 PR, 9 SD, and 3 PD
MK0752 + gemcitabine	Phase I/II; pancreatic cancer	44	Safety/MTD	NA
RO4929097	Phase II; renal cell carcinoma	5	Efficacy	NA
RO4929097	Phase II; pancreatic cancer	18	Survival rate	3 SD,
RO4929097	Phase II, colorectal cancer	37	Efficacy	6 SD,
RO4929097	Phase II; NSCLC	6	Efficacy	NR
RO4929097	Phase I; advanced solid tumor	17	Pharmacokinetics study	NR
RO4929097	Phase I; advanced solid tumor	28	Safety	NR
RO4929097 + capecitabine	Phase I; refractory solid tumor	30	MTD	3 PR,
RO4929097 + gemcitabine	Phase I; advanced solid tumor	18	Safety	1 PR, 3 SD
RO4929097 + temsirolimus	Phase I; advanced solid tumor	17	Safety	11 SD,

Abbreviations: NR, not reported; MTD, maximum tolerated dose; DLT, dose limiting toxicity; NSCLC, non-small cell lung cancer.

1.7.3 Systemic versus topical drug delivery

LY3056480, the active ingredient of the drug product in this study, will not be administered systemically, but topically through a trans-tympanic injection in the middle ear to the Round Window Membrane (RWM). Unlike the daily systemic dosing in the previous clinical programs with this compound class, topical dosing frequency in this study will not be daily. Instead, subjects will receive a maximum of 3 doses in 2 weeks. Therefore, the total maximum dose (250µg) given three times in a 15-day period will be significantly lower when compared to the dose received by subjects over a 15-day period in the AzD studies (15 x 140mg). Even when assuming full systemic absorption of the drug from the middle ear, the total (systemic) body exposure is 2800 times lower than the systemic dose administered with other GSIs in AzD studies over a 15-day period. Moreover, in the Phase III AzD studies, patients were exposed to this class of drugs for much longer, even up to 76 weeks (Henley et al., 2014). Based on this rationale, as well as the LY3056480 non-clinical safety profile and the results of part A, no or limited systemic drug-related AEs are expected. The toxicological effects of the drug that could be expected are local effects as a result of the targeted delivery into the middle ear. These potential effects have been studied in the non-clinical safety studies. See the IB for details on the non-clinical safety studies and results.

1.7.4 Inner ear hair cell loss and SNHL in human patients

Otoacoustic emission studies and postmortem analyses of the cochleae from hearing impaired subjects provide us with evidence for the relation between SNHL and (outer) hair cell loss in

humans (Merchant, Adams, & Nadol, 2005). The *in vivo* models used for the non-clinical studies mimic this situation. In these non-clinical studies in mice and guinea pigs, partial hearing restoration upon GSI treatment has been demonstrated, but there is no certainty that these observations will translate to human patients. For example, the *in vivo* experiments were performed in postnatal (young adult) mice and the *in vitro* studies are performed with tissue from neonates. Regenerative capacity and responsiveness to Notch inhibitors may decline with age. Moreover, the hearing range of mice and guinea pigs is much larger than in humans and across different frequencies, which may limit the extrapolation of the results obtained in these species. Moreover, as dimensions of the cochlea are different across species, drug delivery and distribution may also differ. *In vitro* models of human hair cell formation are not well developed in the field, and this limits the ability to test compounds on human cells. We cannot be sure that these data translate to mature humans. The currently available models do not offer the opportunity to address this translational question. To overcome these challenges the next meaningful step in bringing a therapy to treat hearing loss closer to patients is to advance the non-clinical proof of concept studies to patients in a safe manner.

1.8 Summary

The limited treatment options for the growing number of patients with SNHL, make SNHL a condition of high medical need that calls for innovative, science-driven approaches. A large percentage of SNHL patients are hearing impaired due to the loss of inner ear hair cells. Regeneration of these lost hair cells by Notch inhibition with LY3056480 is a possible strategy towards treating these patients. *In vivo* and *in vitro* experiments have shown that inhibiting Notch can result in the generation of (functional) new hair cells. The local delivery strategy can circumvent systemic side effects seen with this class of molecules in earlier clinical studies. The local application also allows for delivery of small doses of the active compound thereby improving the risk/benefit ratio of this approach.

2 SUMMARY OF REGAIN PART A TRIAL (CONCLUDED NOVEMBER 2018)

Part A (an open-label single centre multiple ascending dose safety study) was designed to study the safety and tolerability of increasing doses LY3056480. The design was a conservative 3+3 design, which was found to be appropriate for drugs where toxicity is uncertain and had led to the definition of safe maximum dose.
15 adult volunteers with mild to moderate (25 to 60 decibels Hearing Level (dBHL)) SNHL of a 5-frequency pure-tone average (0.5 kHz, 1 kHz, 2 kHz, 4kHz and 8 kHz) have been recruited through Adult Audiology Services in the United Kingdom (UK).

The objectives of Part A were:

1. To assess the safety and tolerance of local treatment with LY3056480 in multiple doses, in terms of occurrence and severity of treatment and procedure-related local and systemic AEs;
2. To identify the highest, safe dose;
3. To explore the efficacy of local treatment of LY3056480 in multiple doses in adults with mild to moderate SNHL.

The main endpoints of Part A were:

- Occurrence and severity of treatment-related (IMP) local and systemic Adverse Events
- Occurrence and severity of procedure related local and systemic AEs
- Occurrence of systemic AEs as measured by potentially clinically significant changes by ECG, vital signs, physical examinations and laboratory tests
- Occurrence of injection sites reactions in and around the treated ear as assessed by otomicroscopy
- Safety endpoints focused on local effects are a change in hearing, facial nerve function and balance

The exploratory efficacy endpoints were a change in hearing, balance and tinnitus.

In Part A the participants were treated in ascending dose cohorts of 25µg, 125µg, 200µg, and 250µg applied in 500 µl. Based on the systematic review of safety data, the Data Safety Monitoring Board (DSMB) has decided that a 250µg dose level is safe.

In total 174 AEs were reported in part A of which 2 were probably related to treatment (IMP) and 52 were probably or definitely related to the procedure. No SAEs were reported, all AEs with a relation to the IMP and/or procedure were resolved.

3 STUDY OBJECTIVES and ENDPOINTS OF REGAIN PART B TRIAL (Phase II)

3.1 Objectives

The aim of REGAIN Part B is to establish the efficacy of local treatment with LY3056480 in patients with mild to moderate SNHL.

The objectives are:

Main objective

1. To establish the efficacy of local treatment with LY3056480 in terms of hearing at 12 weeks

Secondary objectives

2. To establish the efficacy of local treatment with LY3056480 in terms of hearing at 6 weeks
3. To continue to assess the safety and tolerance of local treatment with LY3056480 in multiple doses, in terms of:
   a) Occurrence and severity of IMP-related local and systemic AEs
   b) Occurrence and severity of procedure related local and systemic AEs

4 STUDY DESIGN

This is a safety and efficacy study of LY3056480 in patients with mild to moderate SNHL (SNHL).
The study is comprised of two parts:

- Part A – an open-label single-centre multiple ascending dose safety study of LY3056480 (Phase I). This study has been completed at UCL (United Kingdom).
- Part B – a multi-centre efficacy study with patients treated with 250µg of LY3056480.

4.1 Location and procedures

Part B of the REGAIN study will be set in Ear, Nose and Throat (ENT) departments at three participating sites in the UK, Germany and Greece. Forty participants will receive the same dose level of the IMP (250 µg). Each dose will be administered three times to each patient.

The worst hearing ear according to PTA and/or speech audiometry will be treated. If there is no difference in tested hearing levels between ears, patients will be asked to identify the worst hearing ear. If there is no patient preference, the ear considered by the Investigator to be best accessible for trans-tympanic administration of the IMP will be treated.
Participants provide informed consent before entering a screening period of maximum 28 days to assess eligibility for entry into the study. Upon enrollment, the first dose of study drug will be administered trans-tympanically by the PI on Day 1. Subsequent doses will be administered at Day 8 and 15.

Participants will be treated for 2 weeks and then followed for safety and efficacy assessment for an additional 10 weeks as indicated in the Schedule of Assessment (Section 7.3.7). Additional follow up visits at month 6 and 12 are optional.

4.2 Dosage

The safety and tolerability data of all participants from REGAIN Part A has been assessed by the DSMB after all participants have been administered all three doses. The DSMB has judged it is safe to continue part B of the study with the 250µg dose.

4.3 Endpoints

The primary efficacy endpoint of Part B is:
Average change in hearing from baseline in the treated ear at 12 weeks across three frequencies (2, 4, 8 kHz), as measured by PTA (dBHL).

The secondary efficacy endpoints are:

- Hearing: Change from baseline at 6 and 12 weeks (treated ear, untreated ear and difference), in terms of:

- Hearing level as tested by PTA (dBHL) at individual frequencies (0.25, 0.5, 1, 2, 3, 4, 6, 8, 12.5 and 16 kHz)
- Average change in hearing level across three frequencies (2, 4, 8 kHz), as measured by PTA (dBHL) (at 6 weeks only)
- Speech audiometry as tested by speech in noise testing to determine signal to noise ratio loss shift
- Middle ear immittance as tested by tympanometry and ART to determine middle ear pressure, volume and compliance values and acoustic threshold reflex shift
- DPOAE - SNR and absolute levels
- Cochlear dead regions as tested by the Threshold Equalising Noise test
- Hearing specific quality of life (per patient), as measured by the (HHIA/E) questionnaire
- Level of tinnitus as measured by the TFI
- Change in Hearing Aid use as measured by the Hearing Aid Outcome Questionnaire (at month 6 and 12 optional visits)

• Balance: Change from baseline at 12 weeks, as measured by a clinical balance assessment - including History and Examination (Eye Movements, Head Thrust, modified Romberg, Unterberger, Bithermal Air Calorics using VNG), and Dizziness Handicap Inventory

Safety and tolerability endpoints are:

• Hearing and balance as defined in the above endpoints
• Facial nerve function: Change from baseline at the treated side up to week 12, in terms of:
  - Facial nerve function as measured by the House-Brackman grading scale
  - Taste, as reported by the participant (no change, altered taste, loss of taste)
• Occurrence and severity of IMP-related local and systemic AEs up to 12 weeks
• Occurrence and severity of procedure related local and systemic AEs up to 12 weeks
• Occurrence of systemic AEs as measured by potentially clinically significant changes in ECG, vital signs, physical examinations and laboratory tests up to 12 weeks
• Occurrence of injection sites reactions in and around the treated ear as assessed by otomicroscopy up to 12 weeks

4.4 Justification of study design

As previously noted in Section 1 of this protocol, there are good indications that the IMP, LY3056480, may lead to trans-differentiation of supporting cells into inner ear hair cells and a subsequent improvement of hearing in patients with SNHL. The biological mechanism through which this occurs has been studied, the efficacy of this class of molecules was studied in animal models, and LY3056480 was tested in various *in vitro* and *ex vivo* models for hair cell regeneration. Local application of the drug to the RWM offers the opportunity to reach the target organ while exposing a subject to limited quantities of the IMP (Juan & Linthicum, 2010).

The design of Part B establishes efficacy parameters at 250 µg.

Application of the IMP via trans-tympanic injection requires local anaesthetic, in line with standard clinical care at participating sites. Considering the potential risks associated with any

trans-tympanic procedure, one ear will be treated with the active drug, and the other ear left untreated, instead of administering a placebo.

By administering the IMP three times in total, allowing one week between doses, maximum exposure through this route of administration is achieved in a safe manner.

Due to the possible risks involved with the procedure and treatment, participants are not healthy volunteers, but patients with mild to moderate SNHL. In patients with severe and profound SNHL, it is expected that there is a near total loss of not only hair cells but also supporting cells (from which hair cells are to be regenerated) due to degeneration. This would make it impossible to monitor any effects (adverse or otherwise) of the IMP on these hair cells and the absence of supporting cells would have a significant negative impact on the potential for the drug to be efficacious. It is, therefore, reasonable to treat participants with mild to moderate SNHL, for whom this near total loss of hair and supporting cells is not expected.

As LY3056480 will have a very low systemic exposure, it is not feasible to measure it in the blood. The most logical way to localise the product would be in the perilymph. It is not possible to safely sample perilymph from the inner ear making it impossible to perform a relevant PK/PD analysis in this study.

5 STUDY POPULATION

5.1 Population

Forty adult volunteers with a mild to moderate (25 to 60 dBHL) SNHL of a 5-frequency pure tone average (0.5 kHz, 1 kHz, 2 kHz, 4 kHz and 8 kHz) recruited in the UK, Germany and Greece.

5.2 Inclusion criteria

In order to be eligible to participate in this study, participants must meet all of the following criteria:

1. Male or female between 18 and 80 years of age
2. A primary complaint of hearing loss of ≤ 20 years in duration, the history suggesting this hearing loss to be of age-related, noise induced or idiopathic origin
3. A bilateral, symmetrical (<15 dBHL difference) SNHL (SNHL) with a pure tone average threshold across the frequencies 0.5, 1, 2, 4 and 8 kHz of between 25 and 60 dBHL with 2 or more frequencies less than 60 dBHL
4. Participants must have been offered the option of hearing aids prior to being approached to participate in this trial. Use of new hearing aids, defined as first-time use, or upgrades are not permitted from three months prior to trial entry until the last follow-up assessment
5. Able to understand and follow study personnel instructions, read and understand study documents and provide informed consent
6. Willing and able to attend all study visits
7. Willing and able to use adequate hearing protection and to refrain from engaging in exposure to loud noise if sufficient hearing protection is not possible
8. Willing to refrain from wearing the hearing aid in the treated ear from the first day of IMP administration until the last study visit
9. Willing and able to protect ear canal and middle ear from water exposure from the first day of IMP administration to one week after the last administration
10. Are not pregnant or breast feeding and do not plan to become pregnant or father a child during the study. Female participants (of childbearing potential) must be willing to use a highly effective method of contraception throughout the study and must be willing to submit to pregnancy test(s). Male participants must agree to ensure the use of a highly effective method of contraception method with their female partners throughout the study as described above, unless they have had a prior vasectomy.

5.3 Exclusion criteria

A potential participant who meets any of the following criteria will be excluded from participation in this study:

1. Presenting with a primary complaint of tinnitus

2. A ‘true’ air-bone gap ≥15 dBHL in 3 or more contiguous frequencies between 0.5, 1, 2, 4 kHz
3. History of suspected or diagnosed genetic cause of hearing loss
4. Suspected or known diagnosis of inner ear pathology, congenital hearing loss, fluctuating hearing loss, Meniere disease or secondary endolymphatic hydrops, perilymph fistula, cochlear barotrauma, radiation-induced hearing loss, retro-cochlear lesion (including vestibular schwannoma)
5. Evidence of acute or chronic otitis media or otitis externa on examination; or a history of middle ear pathology and/or surgery (history of ventilation tubes allowed)
6. Any therapy known as ototoxic (e.g. aminoglycosides, cisplatin, loop diuretics, quinine, etc.) within 12 months of screening
7. Ongoing or planned systemic or local drug-based therapy for inner ear hearing loss or tinnitus during the study
8. Ongoing or planned anticoagulative medication. Aspirin and NSAID use is permitted prior to and during the study
9. Participants with a history of cancer in the past five years, except for adequately treated basal cell or squamous cell carcinoma of the skin, or in women having resected cervical atypia, or resected carcinoma in situ of the cervix
10. Participants considered by the Investigator to have an ear canal too narrow to allow for trans-tympanic drug application
11. Participants who are physically unable to complete the bi-thermal air caloric testing using VNG
12. Documented history of alcohol and drug abuse within 12 months of screening
13. Any clinically significant co-morbidity that would interfere with trial participation
14. Known hypersensitivity, allergy or intolerance to the study medication or vehicle or any history of severe abnormal drug reaction
15. Concurrent participation in another clinical trial or participation in another clinical trial within 30 days prior to study entry
16. Prior participation in this trial

5.4 Lifestyle guidelines

If hearing aids are used, participants will be reminded to refrain from wearing a hearing aid in the treated ear from the first day of IMP administration until the week 12 study visit.

If needed, an audiologist will remove any hearing aids on day 1 and reinstate after the last dosing. If a change in hearing occurs, hearing aids will be re-tuned and the settings recorded. Any other re-tuning of hearing aids outside of the study, e.g. scheduled appointments with Audiology Services, is not permitted.

The treated ear must be kept dry from the first day of IMP administration to one week after the last IMP administration, and patients are recommended to use cotton wool with Vaseline to create an earplug for protection when showering. Underwater exposure (such as via swimming or diving) is not permitted. Patients should also avoid forceful nose blowing, closed-mouth

sneezing, or auto-inflation of the ears to prevent pressure to the injection site and thereby reduce the risk of a tympanal membrane perforation.

Risks to the male reproductive system are unknown at this time. There were no testis effects in male dogs administered oral doses of up to 40 times the maximum planned human trans-tympanic dose. Male participants must take precautions and agree to ensure the use of a highly effective contraception method with their partner for a period of 13 weeks after the last study drug treatment, unless the participant has had a prior vasectomy.
Female participants of childbearing potential must agree to use a highly effective method of contraception throughout the study and must be willing to submit to pregnancy test(s).
Please refer the appendix A for the definition of women of childbearing potential and of fertile men, and the list of highly effective contraception methods.

6 TREATMENT OF PARTICIPANTS

6.1 Investigational product LY3056480

Part B of the REGAIN study is designed to be an open-label efficacy and safety study of the Notch Inhibitor LY3056480 in patients with mild to moderate SNHL (SNHL). The investigational product is an injectable formulation of LY3056480 an inhibitor of gamma-secretase supplied in 2 mL vials as described in detail in Section 6.1. LY3056480 is locally administered by trans-tympanic injection into the middle ear, in the round window niche by a trained ENT surgeon. This procedure will be detailed in the IMP administration manual. Participants will receive 3 injections of LY3056480 in one ear. The control ear will be left untreated. The treatment will be given over the course of two weeks.

In Part B participants will receive the highest tolerable dose that resulted from Part A of the trial, 250µg.

This study has no restrictions on the use of other medication, or escape medication.

7 INVESTIGATIONAL PRODUCT

7.1 Name and description of investigational product

LY3056480 is an inhibitor of gamma-secretase with the following characteristics:

Molecular weight:	467.49
Molecular/Empirical formula:	$C_{23}H_{28}F_3N_3O_4$
Description:	Off-white to yellow solid
cLog P	3.3
Specific Rotation, $[\alpha]^{25}_D$:	$[\alpha]^{25}_D = 101.4°$
Permeability	2.06×10^{-6}/cms^{-1} Caco-2
Stability:	LY3056480 drug substance is stable at ambient temperature (15 to 30 °C). As described in Section 4.2.2, the drug product should be store at ambient temperature (15 to 30 °C).
Solubility:	See the table 3. for solubility results

Table 3. Solubility of LY3056480

Solvent	Solubility (mg/mL)	Solubility Description[a]
Water	0.111	Practically insoluble
0.1 N HCl	0.094	Practically insoluble
pH 7.5 phosphate	0.085	Practically insoluble
Ethanol	>10	Sparingly soluble

[a] Solubility descriptions are consistent with the US Pharmacopeial Convention, European Pharmacopoeia, and Japanese Pharmacopoeia

The investigational product consists of an injectable formulation of LY3056480, 0.5 mg/mL for Injection. Products are supplied for clinical use as single-use clear solution formulation in 2 mL glass vials. Each vial of LY3056480 for Injection, 0.5 mg/mL contains 1.0 mL of 0.5 mg/mL LY3056480 solution. LY3056480 for Injection, 0.5 mg/mL drug product is composed of LY3056480 and the excipients ethanol, dimethyl sulfoxide, poloxamer 188 and sterile water for injection.

The label text will be open-label as the pharmacy is unblinded.
All study medication supplied must be stored at ambient temperature conditions of 15 °C to 30 °C in a secure area with limited access.

7.2 Summary of findings from non-clinical studies

Preclinical studies relevant to the use of LY3056480 have been conducted. Further information is available in the current version of the LY3056480 IB.

7.3 Summary of findings from clinical studies

A First in Man trial has been conducted treating 15 patients with mild to moderate NSHL with three doses of LY3056480 in 15 Days. In this dosing escalation trial (REGAIN part A), no SAEs were reported, and no safety signals were identified. An independent DSMB concluded that the 250µg dose level was tolerable and considered safe to use in part B of the trial.

7.4 Summary of known and potential risks and benefits

Potential risks with the compound class have been described in Section 1.7.2. These risks mainly relate to systemic exposure which has been significantly reduced by the local delivery of the compound. The LY3056480 associated risks have been investigated in non-clinical toxicity studies focussing on systemic and local delivery and have not resulted in the identification of additional risks. Following part A of the trial (the First-in-Man study investigating LY3056480), the DSMB has judged the compound to be considered adequately safe for further evaluation in clinical trials.

7.5 Dosages, dosage modifications and method of administration

In Part B participants will be treated with 250 µg applied in 500 µL.

7.6 Efficacy study

From Part A of the study, the highest safe dose has been determined. As no MTD has been reached in Part A, the study is a single dose study.

Data obtained in the study will be reviewed on a regular basis by the Sponsor and MM for the ongoing analysis of safety. At each safety review session, the cumulative safety data for all the prior treated subjects is reviewed. TEAEs that, at least, could be related to the administration of LY3056480 will be assessed for each subject at each visit. AE considered to be related to the drug at a Grade 3 or 4 (using CTCAE version 5.0) would be considered sufficient to lead to review and possible discussion with members of the DSMB.
The MM will also review SAEs in an expedited fashion.

7.7 Preparation and labelling of Investigational Medicinal Product

In Part B of the study, either the PI or pharmacy staff will be responsible for the preparation of the drug for administration. Further details on IMP preparation will be described in separate IMP preparation manuals.

Example label:

LY3056480 for Injection, 0.5mg/mL

Carton label:

Lot No: XXXXXX
Re-test date: MM/YYYY
Protocol: AUT-001
EudraCT number: XXXXXXXXXXX
Investigator: _________________
This carton contains XX vials each containing 1.0 ml of LY3056480 for Injection, 0.5mg/mL, single use vial.
For transtympanic injection use only.
Store at room temperature at 15° to 30°C.
See Pharmacy Manual for handling and dosing directions.
For clinical trial use only.
Sponsor: Audion Therapeutics BV
Linnaeusparkweg 10-2, 1098 EA, Amsterdam, Netherlands
Phone: + XX XXXXXXXX

Vial label:

Lot No: XXXXXX
Protocol: AUT-001
Contains 1.0 ml LY3056480 for Injection, 0.5mg/mL, single use vial.
For transtympanic injection use only.
Store at room temperature at 15° to 30°C.
Audion Therapeutics BV

7.8 Drug accountability

After all the necessary regulatory approvals and upon Sponsor's request, defined quantities of study medication (investigational product) will be shipped to the clinical centres in appropriate conditions in order to guarantee the quality of the investigational medicinal products. Relative humidity and temperature will be controlled during the IMP shipment, if needed using a data logger.

In accordance with the International Conference on Harmonisation (ICH) Good Clinical Practice (GCP) guidelines, after each study medication receipt, the research staff of each centre will account for all study medication. Details of receipt, storage, dosing preparation, administration and return will be recorded. Investigational products will be provided to study participants only. An adequate quantity of reserve samples for the investigational medicinal products will be retained. The packaging, storage conditions and retention period of the reserve samples shall comply with the current version of the applicable regulations.

During the study and at termination, both the unused and partially unused investigational products (except for the retention samples) will be destroyed. This activity will not be performed until final drug accountability of the batch to be destroyed by the monitor has been performed.

8 METHODS

8.1 Blinding

This study is not blinded.

8.2 Study procedures

8.2.1 Patient Identification

Participants will be identified via

- ethically approved advertisements and existing databases of people interested in taking part in hearing-loss research.
- existing clinical care team at participating sites and patient identification centres and asked to consent to contact by the study team.

8.2.2 Informed consent

Potential participants will be sent information regarding the study and asked to visit the research site to provide informed consent and carry out screening procedures to assess eligibility (visit 1). All participants will be given adequate time (at least 24 hours) to consider participation in the study and ask any questions they may have before providing consent to take part.

It is the responsibility of the PI, or a person delegated by the PI to obtain written informed consent from each subject prior to participation in the trial, following adequate explanation of the aims, methods, anticipated benefits and potential hazards of the study. All persons involved in the informed consent procedure will be delegated by the PI, GCP trained, suitably qualified, experienced and will be on the delegation log held at the site.

The PI or designee will explain to the participants that they are no obligation to enter the trial and that they can withdraw at any time during the trial, without having to give a reason.

No clinical trial procedures will be conducted prior to taking consent from the participant. Consent does not denote enrolment into the trial. A copy of the signed Informed Consent form will be given to the participant. The original signed form is retained at the study site in the site file and a copy placed in the medical notes. If new safety information results in significant changes in the risk/benefit assessment, the patient information sheet and consent form will be reviewed and updated if necessary and participants will be asked to re-consent as appropriate.

8.2.3 Screening

Tests and assessments will be performed at the initial visit to assess eligibility for the study. All patients are entered onto a screening log. After all test results have been reviewed and eligibility confirmed by the PI, participants will be asked to return to the site within 28 days for visit 2 (baseline/treatment).

8.2.4 Treatment – first dose (Day 1 / Baseline)

Participants will be admitted to the hospital outpatient ward on the morning of the dosing, following local procedures for admittance.

Prior to dosing, baseline assessments will be carried out in line with the schedule of assessments. If a hearing aid is used, participants will be reminded to refrain from wearing a hearing aid in the treated ear from the first day of IMP administration until the week 12 visit detailed in the lifestyle guidelines.

Details of the procedure, the anaesthetic and the drug administration are covered in the IMP administration manual.

8.2.5 Follow-up

Participants will be followed up per the Schedule of Assessments (Table 4, 5 and 6).

Additionally, participants will be asked to come back to the clinic after 6 and 12 months for a general safety follow up. This follow up is optional and separate Informed Consent will be asked.

8.2.6 Assessments

The following assessments will be taken as indicated in the Schedule of Assessment (Table 4).

- Full/brief physical examination
- Demographics – date of birth, race, height, weight, smoking history, socio economic status
- Vital signs – heart rate, blood pressure, respiration rate, ECG and body temperature
- Laboratory assessments (haematology, biochemistry, urinalysis, pregnancy test)
- Concomitant medication
- AEs
- Otomicroscopy - injection site
- PTA
    - air conduction measured at 0.25 kHz, 0.5 kHz, 1 kHz, 2 kHz, 3 kHz, 4 kHz, 6kHz, 8 kHz, 12.5 kHz and 16 kHz
    - bone conduction measured at 0.5 kHz, 1 kHz, 2 kHz, and 4 kHz
- Speech audiometry as tested by speech in noise testing - signal to noise ratio loss shift
- DPOAE - SNR and absolute levels
- Cochlear dead regions - TEN Test
- Middle ear immittance - Tympanometry and ART
- Tinnitus assessment – history, if positive; patient-reported Tinnitus Functional Index (TFI)
- Clinical balance assessment – History and Examination (Eye Movements, Head Thrust, modified Romberg, Unterberger),

- Full clinical balance assessment – History and Examination (Eye Movements, Head Thrust, modified Romberg, Unterberger, Bithermal Air Calorics using VNG), Dizziness Handicap Inventory
- Facial nerve function - House-Brackman scale
- Taste Assessment –patient-reported (no change, altered taste, loss of taste)
- Disease-specific - HHIA/E questionnaire and Hearing Aid Questionnaire

Table 4. Part B Schedule of Assessments

		Assessor[1]	Screening period	Treatment period				Follow up period		Optional visits	
No.	**Activity** *Visit window*		*Day -28 to 0*	**Day 1**		**Day 8** *(+/- 1 day)*	**Day 15** *(+/- 2 days)*	**Week 6** (+/- 2 days)	**Week 12** (+/- 7 days)	**Month 6**	**Month 12**
				**Baseline**	**Dose 1**	**Dose 2**	**Dose 3**				
Study Assessments											
1	Informed Consent	RN/PI	X								
2	Review of in/exclusion criteria	RN/PI	X	X							
3	Medical history and demography, drug record	PI	X								
4	Vital signs	RN	X	X	X	X	X	X	X		
5	Physical examination[2]	ENT	X	X		X	X	X	X	X	X
6	12-lead ECG	RN	X		X	X	X	X	X		
7	Laboratory assessments [3, 4]	RN	X		X	X	X	X	X		
8	Clinical balance assessment [5].	ENT			X	X	X	X		X	X
9	Full balance assessment [6]	AL	X						X		
10	Facial nerve function	ENT	X		X	X	X	X	X	X	X
11	Taste assessment	ENT			X	X	X	X	X	X	X
12	Otomicroscopy	ENT	X	X		X	X	X	X	X	X
13	PTA [7]	AL	X		X	X	X	X	X	X	X
14	Speech Audiometry [8]	AL	X					X	X	X	X
15	DPOAE, SNR and absolute	AL		X				X	X	X	X
16	Cochlear dead region assessment (TEN test)	AL		X				X	X		
17	Middle ear immittance - ART	AL		X					X		
18	Middle ear immittance - tympanometry	AL	X						X	X	X
19	Tinnitus assessment [9]	AL	X	X	X	X	X	X	X	X	X
20	QoL (HHIA/E questionnaire)	AL	X					X	X	X	X
21	Hearing Aid questionnaire	AL	X							X	X
22	Drug dosing - Injection	PI			X	X	X				
23	Pregnancy testing	RN	X	X		X	X	X	X		
24	Concomitant medication	RN	X	X	X	X	X	X	X	X	X
25	Adverse Events	RN	X	X	X	X	X	X	X	X	X

Footnotes Table 4

1. RN: Research Nurse, PI: Principal Investigator, ENT: Ear, Nose, and Throat, AL: Audiologist
2. Head, ears (otomicroscopy), eyes, nose and throat; thyroid; lymph nodes; heart; chest; lungs; abdomen; skin; and extremities; musculoskeletal and neurological, cardiovascular, respiratory & gastrointestinal systems. Full at screening, brief at the other visits.
3. Extensive lab Day 1, Day 8 and Day 15: haematology: Hgb, HCT, RBC, WBC, differential count, platelet count, ESR. Urinalysis: glucose, and creatinine. chemistry: Sodium, potassium, chloride, total bilirubin, alkaline phosphatase (ALP), ALT (SGPT), AST (SGOT), gamma-GT (GGT), LDH, CRP, CK, BUN, creatinine, glucose, uric acid, calcium (Ca++), phosphorous, total protein, albumin, HbA1C, cholesterol, HDL-cholesterol, LDL-cholesterol, triglycerides. Pregnancy test serum at screening, urine other visits
4. Limited lab: haematology: Hgb, HCT, RBC, WBC, differential count, platelet count, ESR. Urinalysis: glucose. Chemistry: sodium, potassium, chloride, ALT, AST, CRP, creatinine, glucose, HbA1C, HDL-cholesterol, LDL-cholesterol, triglycerides.
5. All visits: Clinical balance assessment –History and Examination (Eye Movements, Head Thrust, modified Romberg, Unterberger).
6. Screening and week 12: Full balance assessment – History and Examination (Eye Movements, Head Thrust, modified Romberg, Unterberger. Bithermal Air Calorics using VNG), Dizziness Handicap Inventory. DHI also when dizziness develops during trial. Additional balance tests may be conducted, if balance is affected.
7. Air conduction measured at frequencies; 0.25, 0.5, 1, 2, 3, 4, 6, 8, 12.5 and 16 kHz. Bone conduction measured at 0.5, 1, 2, 4kHz
8. Speech Discrimination and Speech Discrimination in Noise - Score and Signal to Noise Ratio (SNR)
9. Tinnitus assessment –Patient-reported TFI at screening, week 6 and 12 and if tinnitus worsens or develops during trial. At all visits short question (no change, louder, less loud)

Table 5. Assessments – first dose Part B

**Assessment**	**Assessor**	*+ 1 hr*	*+ 2 hr*	*+ 4 hr*
Vital signs	RN	X	X	X
AE assessment	RN	X	X	X
Laboratory assessments	RN			X
Brief neurological examination	ENT			X
12 lead ECG (triplicate)	RN			X
PTA (air and bone conduction in both ears)	AL			X
Taste assessment	RN			X
Facial Nerve Function	ENT			X
Clinical Balance Assessment	ENT			X
Tinnitus assessment (question)	RN	X	X	X

Table 6. Assessments – second and third dose Part B

**Assessment**	**Assessor**	*Pre-dose*	*+ 1 hr*	*+ 4hr*
Vital signs	RN	X	X	X
AE assessment	RN	X	X	X
Laboratory assessments	RN			X
Brief neurological examination	ENT			X
12 lead ECG	RN			X
PTA (air and bone conduction in both ears)	AL	X		X
Facial Nerve Function	ENT			X
Taste assessment	RN			X
Clinical Balance Assessment	ENT			X
Tinnitus assessment (question)	RN	X	X	X

Participants will receive a follow-up call from a member of the study team 24 hours after drug administration to capture any AEs during this period.

8.3 Definition of end of trial

The end of the trial is defined as the last visit of the last participant enrolled in the study (Part B).

8.4 Withdrawal of individual participants

Participants can leave the study at any time for any reason if they wish to do so without any consequences. The Investigator can decide to withdraw a subject from the study for urgent medical reasons.

Reasons for participants to be withdrawn from the study include, but are not limited to;

- The subject withdraws consent.
- Violation of eligibility criteria (if the violation is detected prior to drug administration).
- At the PI's or MM's discretion to protect subject safety and well-being.
- Participants may also be discontinued due to the following: change in compliance with inclusion/exclusion criterion that is clinically relevant and affects safety, the occurrence of AEs, etc.

Reasons for discontinuation will be recorded.

Once the dosing is complete, a subsequent violation of the protocol should not be considered grounds for withdrawal. All participants treated should be followed up for safety evaluation, if at all possible. If withdrawn due to an AE, participants must be followed until resolution of all their AEs or until the unresolved AEs are judged by the Investigator is stabilised.

In all cases, the date the subject is withdrawn from the study and the reason(s) for withdrawal must be recorded on the electronic case report form (eCRF).

If a subject is withdrawn from the study for any reason, the Investigator must make every effort to perform the evaluations described for the week 12/Early Termination Visit. Participants withdrawn because of adverse experiences will undergo a physical examination and laboratory tests planned at the follow-up visit (if required). A follow-up of AEs will also be undertaken.

8.5 Replacement of individual participants after withdrawal

Participants who are not evaluable may be replaced. A total of 40 participants are aimed to complete part B of the study.

8.6 Follow-up of participants withdrawn from treatment

All participants will be followed up for a total of 12 weeks from first treatment with study medication. Additional follow up at month 6 and month 12 are optional.

8.7 Premature termination of the study/stopping rules

The study may be discontinued at the discretion of the CI, PI, Sponsor, or IEC based on the occurrence of the following (but not limited to):

- AEs unknown to date with respect to their nature, severity, and duration
- Increased frequency and/or severity and/or duration of AEs
- Medical or ethical reasons affecting the continued performance of the study
- Cancellation of drug development
- Notification by regulatory authorities

The written information concerning premature termination of the study will be provided to applicable recipients, such as Investigators, Sponsor, or IECs.

9 SAFETY REPORTING

9.1 Temporary halt for reasons of participant safety

In accordance with regulations, the Sponsor will suspend the study if there is sufficient ground that continuation of the study will jeopardise participant health or safety. The Sponsor will notify the CA and IEC without undue delay of a temporary halt including the reason for such an action. The study will be suspended pending a further positive decision by the IEC. The investigator will take care that all participants are kept informed.

The Sponsor or investigator may take other appropriate urgent safety measures in order to protect research participants against any immediate hazard to their health or safety, without prior authorisation from a regulatory body. If any urgent safety measures are taken, the Investigator/Sponsor shall immediately, and in any event, no later than 3 days from the date the measures are taken, give written notice to the CA and the relevant IEC of the measures taken and the circumstances giving rise to those measures.

9.2 Definitions

Table 7. Safety Terms and Definitions

Term	Definition
**Adverse Event (AE)**	Any untoward medical occurrence in a patient or clinical trial participant administered a medicinal product and which does not necessarily have a causal relationship with this treatment.
**Adverse Drug Reaction**	All noxious and unintended responses to a medicinal product related to any dose should be considered adverse drug reactions.
**Adverse Reaction (AR)**	**Definition**
**Serious adverse event (SAE), serious adverse reaction (SAR) or unexpected serious adverse reaction**	Any adverse event, adverse reaction or unexpected adverse reaction, respectively, that:<br>• results in death,<br>• is life-threatening,<br>• requires hospitalisation or prolongation of existing hospitalisation*,<br>• results in persistent or significant disability or incapacity, or<br>• consists of a congenital anomaly or birth defect.
**Important Medical Event**	These events may jeopardise the participant or may require an intervention to prevent one of the above characteristics/consequences. Such events should also be considered 'serious'.
**Unexpected adverse reaction**	An adverse reaction the nature and severity of which is not consistent with the information about the medicinal product in question set out:<br>a) in the case of a product with a marketing authorization, in the summary of product characteristics for that product,<br>b) in the case of any other IMP, in the IB relating to the trial in question.

**SUSAR**	Suspected Unexpected Serious Adverse Reaction

*Hospitalisation is defined as an event requiring at least one overnight stay in a hospital unit. An overnight stay due to convenience (e.g. the subject resides far away from the treating clinic) rather than medical necessity does not meet the seriousness criterion. Surgical procedures planned prior to the enrolment in the trial are not considered SAEs unless the procedure was re-scheduled due to significant deterioration of the condition indicating the procedure.

9.3 Recording Adverse Events

All AEs will be recorded in the medical records and in the CRF following the moment of informed consent. Recording of AEs will include clinical symptoms, description of the event and medical management, including dates as appropriate. All AEs will be categorised according to their severity, as defined in Section 8.4.

AEs will be coded using the Medical Dictionary for Regulatory Activities (MedDRA) dictionary and tabulated according to body system and preferred term. Participants with TEAEs during the post−treatment period will be summarised by body system and preferred term. TEAEs are defined as:

- Events that start on or after the first day of treatment but were not present before treatment commenced
- Events that start before the day of the first treatment but increase in severity on or after the initial day of treatment.

9.4 Assessment of Adverse Events

Each adverse event will be assessed for the following criteria by a medical doctor:

9.4.1 Severity

Grade refers to the severity of the AE. The grades used in this trial will be taken from the general guidelines included in the CTCAE version 5.0.

Table 8. Adverse Event Grading

Grade	Definition
**1 - Mild**	Asymptomatic or mild symptoms; clinical or diagnostic observations only; intervention not indicated.
**2 - Moderate**	Minimal, local or non-invasive intervention indicated; limiting age-appropriate instrumental Activities of Daily Living (ADL)*.
**3 - Severe**	Medically significant but not immediately life-threatening; hospitalisation or prolongation of hospitalisation indicated; disabling; limiting self-care ADL**.
**4 - Life-threatening consequences**	Urgent intervention indicated.

**5 - Death**	Death related to AE.

*Instrumental ADL refer to preparing meals, shopping for groceries or clothes, using the telephone, managing money, etc.

** Self-care ADL refer to bathing, dressing and undressing, feeding self, using the toilet, taking medications, and not bedridden.

9.4.2 Causality

The assessment of relationship of AEs to the administration of IMP is a clinical decision based on all available information at the time of the completion of the case report form. The following categories will be used to define the causality of the adverse event:

Table 9. Adverse Event Causality

Category	Definition
**Definitely**	There is clear evidence to suggest a causal relationship, and other possible contributing factors can be ruled out.
**Probably**	There is evidence to suggest a causal relationship, and the influence of other factors is unlikely
**Possibly**	There is some evidence to suggest a causal relationship (e.g. the event occurred within a reasonable time after administration of the trial medication). However, the influence of other factors may have contributed to the event (e.g. the patient's clinical condition, other concomitant events).
**Unlikely**	There is little evidence to suggest there is a causal relationship (e.g. the event did not occur within a reasonable time after administration of the trial medication). There is another reasonable explanation for the event (e.g. the patient's clinical condition, other concomitant treatments).
**Not related**	There is no evidence of any causal relationship.
**Not Assessable**	Unable to assess on information available.

According to ICH-E2A-Guideline Clinical safety data management": definition and standards for expedited reporting, there are two categories: "related" and "not related". Not related is categorized as "unlikely or "not related".

9.4.3 Expectedness

Table 10. Adverse Event Expectedness

Category	Definition
Expected	An adverse reaction, the nature or severity of which is consistent with the applicable product information (e.g., Investigator's Brochure for an unapproved investigational medicinal product).
Unexpected	An adverse reaction, the nature or severity of which is not consistent with the applicable product information (e.g., Investigator's Brochure for an unapproved investigational medicinal product).

The reference document to be used to assess expectedness against the IMP is the most current IB.

9.4.4 Seriousness

Seriousness will be assessed as defined in Section 8.2.

Collection, recording and reporting of AEs to the Sponsor will be completed in line with Sponsor or delegate's SOPs.

9.5 Abnormal test findings

The criteria for determining whether an abnormal objective test finding should be reported as an AE are as follows:

- Test result is associated with accompanying symptoms, and/or
- Test result requires additional diagnostic testing or medical/surgical intervention, and/or
- Test result leads to a change in study dosing (outside of protocol-stipulated dose adjustments) or discontinuation from the study, significant additional concomitant drug treatment, or other therapy, and/or
- Test result is considered to be an AE by the investigator or Sponsor.

Merely repeating an abnormal test, in the absence of any of the above conditions, does not constitute an AE. Any abnormal test result that is determined to be an error does not require reporting as an AE.

9.6 AEs, SAEs and SUSARs

9.6.1 Adverse Events

AEs are defined as any undesirable experience occurring to a participant during the study, whether or not considered related to the investigational product, the trial procedure or the experimental intervention. All AEs reported spontaneously by the participant or observed by the investigator, or his staff, will be recorded.

9.6.2 Recording and Reporting Serious Adverse Events

The Investigator will report all serious adverse events (SAEs) to the Sponsor within 24 hours of awareness. The Investigator will respond to any SAE queries raised by the Sponsor as soon as possible.

9.6.3 Suspected unexpected serious adverse reactions (SUSARs)

Adverse reactions are all untoward and unintended responses to an investigational product related to any dose administered.

Unexpected adverse reactions are SUSARs if the following three conditions are met:

1. the event must be serious (see Section 8.2);
2. there must be a certain degree of probability that the event is a harmful and undesirable reaction to the medicinal product under investigation, regardless of the administered dose;
3. the adverse reaction must be unexpected, that is to say, the nature and severity of the adverse reaction are not in agreement with the product information as recorded in the IB.

The Sponsor will report SUSARs in an expedited manner to EudraVigilance, Competent Authorities and IECs in accordance with local legislation.

A SUSAR, which is fatal or life threatening, will be reported as soon as possible and within 7 calendar days of Sponsor awareness. Additional information will be sent within 8 calendar days of the first report. All other SUSARs should be reported as soon as possible, and within 15 calendar days of Sponsor awareness.

9.7 Annual safety report

In addition to the expedited reporting of SUSARs, the Sponsor will submit, once a year throughout the clinical trial, a safety report to the IEC, in accordance with local legislation, and competent authorities of the concerned Member States.

This safety report consists of:

- a list of all suspected (unexpected or expected) serious adverse reactions, along with an aggregated summary table of all reported serious adverse reactions, ordered by organ system, per study;
- a report concerning the safety of the participants, consisting of a complete safety analysis and an evaluation of the balance between the efficacy and the harmfulness of the medicine under investigation.

9.8 Follow-up of Adverse Events

All AEs will be followed until the event or its sequelae resolve, or until a stable situation has been reached in the opinion of the Investigator. Depending on the event, follow-up may require additional tests or medical procedures as indicated, and/or referral to the general physician or a medical specialist.

All AEs will be recorded and reported until the end of the study, as defined in Section 7.4. Any SUSAR related to the IMP will need to be reported to the Sponsor irrespective of how long after IMP administration the reaction has occurred.

9.9 Data Safety Monitoring Board

In Part A, an DSMB that included appropriately qualified members independent of the Sponsor was constituted to consider safety data generated during the study relevant to the escalation of dosing i. The DSMB had the responsibilities for oversight of safety and to provide guidance to the study Sponsor. The procedures and responsibilities for the collection, analysis, and review of the data by the DSMB are described in a separate DSMB charter.

The DSMB will be provided with a summary of the collected safety and tolerability assessments available for each participant at a frequency as indicated in the DSMB Charter. The DSMB will be informed expeditiously if any participant has withdrawn due to a TEAE or if a SUSAR occurs in any of the treated participants during Part A. The DSMB may amend the Charter and request additional data as per their requirements.

10 STATISTICAL METHODOLOGY AND ANALYSIS

10.1 Statistical analysis plan

A detailed Statistical Analysis Plan (SAP) will be written and finalised before database lock. The SAP will provide full details of all planned data analyses and data display.

10.2 Statistical power calculations

Part A: No formal power calculation was performed to estimate sample size as this dose escalation study was using a 3+3 design.

Part B:. A total of 40 evaluable participants will be recruited to test one dose.

The analysis will allow for direct investigation of changes in hearing from the baseline in the treated ear at 12 weeks as measured by PTA.

This phase I/II study is the first to investigate the safety and efficacy of the GSI injection with regards to the possible recovery of hearing loss and as such there are no previous trials from which to estimate the effect size of the treatment. In a recent trial that investigated intra-tympanic injections using a different compound the sample size estimation was based on an effect size of 0.60 (Suckfuell et al., 2014). Using this effect size as a reference, the sample size estimation in the present trial is based on effect sizes within the range of 0.45 to 0.65. An effect size of 0.50 will correspond to an improvement in hearing function of 10 dB with an associated standard deviation of 20 dB. For this trial, an improvement in hearing function of at least 10dB as measured by PTA has been determined as clinically relevant.

The Table 12 below shows the resulting power calculations for detecting an improvement in hearing using a two-sided test and a significance level of 5% for effect sizes between 0.45 to 0.65.

Table 11. Power calculation

**Power to detect improvement in hearing for effect sizes from 0.45 to 0.65**					
Effect size	0.45	0.50	0.55	0.60	0.65
n=40	80%	87%.	92%	96%	98%

Assuming that 40 participants complete the trial there is a power of 87% to detect an improvement in hearing corresponding to an effect size 0.5.

10.3 Analysis sets

All analyses will be based on planned treatment regimen rather than the actual treatment given in case of any differences. The following analysis sets are defined:

Part B: Intention-to-treat analysis set (full analysis set)
The ITT analysis set will include all participants.

Part B: Modified intention-to-treat analysis set
The mITT analysis set will include participants from the ITT analysis set who have a baseline and at least one post-dose PTA assessment available.

Part B: Per-protocol analysis set
The per-protocol analysis set will include all participants from the mITT analysis set who have been treated according to the study protocol and fulfil the following criteria:

- Compliance with all entry criteria
- Absence of major protocol deviations with respect to factors likely to affect the efficacy of treatment
- Adequate treatment compliance, defined as two or more doses of the study drug

Part B: Safety analysis set
The safety analysis set will include all participants undergoing receiving at least one dose of study treatment.

10.4 Missing data

As a general rule, all data collected and available will be used in the analysis. No imputation of missing data will be applied independently of the statistical methods used to analyse the data.

Handling of missing data from questionnaires will depend on whether only single items are missing or a complete questionnaire is missing. If only single items are missing, the rules defined by the authors of the questionnaires (if any) will be followed. The details on the handling of missing data will be described in the SAP.

10.5 Summary statistics

The summary statistics presented for quantitative variables will be the number of observations (n), the number of missing values (missing), mean, standard deviation (SD), median, first and third quartiles (Q1, Q3), and minimum (min), and maximum values (max).

The summary statistics presented for categorical data will be the number of observations (n), the number of missing values (missing), and the count and percentage of participants in each category.

10.6 Analysis of primary endpoint

The primary endpoint of Part B is:

- Change in hearing from baseline in the treated ear at 12 weeks across three frequencies (2 kHz, 4 kHz, 8 kHz) as measured by PTA

The primary efficacy endpoint analysis will be performed using the mITT population. The hearing in dBHL will be summarised by time point along with the absolute change from baseline. The baseline value is defined as the last value prior to first dose of study treatment.

The treatment effect on the primary endpoint will be assessed through a mixed-effect model for repeated measures analysis on absolute change from baseline, including baseline value, and the time point as factors. Subject will be included as random factor.

The primary efficacy analysis will consist of testing the effect at week 12. The significance level will be set at 5%. Adjustments for multiplicity will not be applied.

For part B of the study, the null and the alternative hypotheses of treatment effect in the dose group is:

H_0: *Mean = 0* (i.e. no statistically significant change in hearing after treatment)
H_1: *Mean ≠ 0* (i.e. a statistically significant change in hearing after treatment)

where *Mean* refers to the mean difference in hearing function between baseline and post-treatment. If the null hypothesis is rejected, the alternative hypothesis will be accepted.

The primary efficacy analysis will also be performed for subjects in the per-protocol population to support the findings from the mITT population.

10.7 Analysis of secondary endpoints

The secondary endpoints will be summarised by time point along with the absolute change from baseline for continuous variables and shift tables for categorical variables. The baseline value is defined as the last value prior to first dose of study treatment.

A mixed-effect model for repeated measures similar to that used for the primary endpoint will be used to assess the treatment effect on continuous secondary endpoints such as the additional assessment based on the PTA measurements, speech audiometry signal to noise ratio, middle ear immittance, middle ear pressure, volume and acoustic threshold reflex shift, DPOAE - SNR and absolute levels, hearing by cochlear dead regions as tested by the TEN test, HHIA/E questionnaire total points, social points, and emotional points, Dizziness Handicap Inventory questionnaire summary scores of total, quality of life, and symptoms, and symptom sub-scale scores of dizziness, anxiety, and motion-provoked dizziness.

A chi-square test will be used to assess the treatment effect on categorical data such as shifts in:

- DPOAE classified into one of three categories of ‘Present and normal’, ‘Present but abnormal’, ‘Absent (SNR<6)’

- HHIA/E questionnaire total score, social score, and emotional score classified into one of the three categories of ‘No handicap’, ‘Mild-Moderate Handicap’, ‘Significant Handicap’
- TFI classified into one of the three categories of ‘Low’, ‘Medium’, ’Severe’
- Hearing Aid outcome questionnaire classified into one of the three categories of ‘Low’, ‘Medium’, ’Severe’
- Eye Movements classified into one of two categories of ‘Normal’, ‘Abnormal’
- Modified Romberg’s test classified into one of the two categories of ‘Positive’, ‘Negative’ (with ‘Positive’ being associated with a loss of balance)
- Unterberger test classified into one of the two categories of ‘Positive’, ‘Negative’ (with ‘Positive’ being associated with a loss of balance)
- Head Thrust Test classified into one of two categories of ‘Normal vestibulo-ocular reflex gain’ Reduced vestibulo-ocular reflex gain’
- VNG Air Calorics classified into one of three categories of ‘Hyperactive’, ‘Normal’, ‘Hypoactive’ (with both ‘Hyperactive and Hypoactive’ being associated with problems in balance)’

10.8 Analysis of safety endpoints

The safety data will be summarised descriptively overall using the safety analysis set. The analysis will be performed as described above..

10.9 Analysis of further endpoints

The following will be described overall using summary statistics:

- Disposition of participants and discontinuations
- Demographics and other baseline characteristics
- Treatment exposure as assessed by the number of doses received

Medical history and surgical history will only be listed.

Further details of the exploratory and/or safety endpoints of Facial nerve function and Taste will be defined in the SAP.

10.10 Interim analysis (if applicable)

No interim analysis planned.

11 ETHICAL CONSIDERATIONS

11.1 Regulation statement

The study will be conducted according to the principles of the Declaration of Helsinki (64th Word Medical Association General Assembly, Fortaleza, Brazil, October 2013, see for the most recent version: www.wma.net), GCP and in accordance with all local guidelines, regulations, and Acts.

11.2 Recruitment and consent

Participants will be recruited from the investigational sites based on the eligibility criteria provided in the protocol. They will be informed about the study as detailed in Section 7.3.2.

11.3 Benefits and risks assessment, group relatedness

Participants in the current trial will be given the opportunity to receive a new local treatment with promising results from animal studies, to potentially reverse the SNHL. The potential to obtain treatment benefit is considered to be the main benefit of participation in the trial.

As the treatment is applied locally at a relatively low dose, the risks of systemic toxicity are considered very low.

There are risks associated with the treatment administration procedure, and risks associated with the unintended pharmacological activity of the IMP in the inner ear, middle ear or in the proximity of the middle ear. The treatment procedure (trans-tympanic injection) is a well-known method of drug delivery to the inner ear, with known risks, and the risks associated with the trans-tympanic injections are evaluated as acceptable when taking the potential benefit into consideration.

11.4 Compensation for injury

The Sponsor and/or Investigator has a liability insurance which is in accordance with relevant legislation. In the event of any suffering, deterioration in health or well-being or any harmful susceptibility or toxicity caused to participants' participation in the trial; the participant will receive appropriate compensation irrespective of the question of legal liability.

The Sponsor and/or Investigator has an insurance which is in accordance with the legal requirements in the participating countries. This insurance provides cover for damage to research participants through injury or death caused by the study. The insurance applies to the damage that becomes apparent during the study or within 3 years after the end of the study. Each partner will ensure proper insurance to cover their liability, and damage for participants participating in their country.

11.5 Incentives

In addition to compensation for travel costs, a financial compensation will be provided to the participants when completing study visits if regulatory approval is obtained in the participating country.

12 ADMINISTRATIVE ASPECTS, MONITORING AND PUBLICATION

12.1 Handling and storage of data and documents

Participant medical information obtained during this study is confidential, and disclosure to third parties other than those noted below is prohibited. With the participant's permission, medical information may be given to his/her personal physician or other appropriate medical personnel responsible for his/her welfare.

The information required by the protocol is entered from the patient file into an electronic data capture (EDC) system. The patient files are to be considered source data in addition to the automatic print outs.

The eCRF will be set up by Nordic Bioscience Clinical Development (NBCD) data management. Detailed information on the eCRF completion will be provided to the sites during the site initiation visits. Each site will also be provided with an eCRF completion manual. In general, all users who have access to the EDC system will be trained. The access to the EDC system is controlled by username and password. Edit checks will be added in the eCRF according to the data validation plan to raise queries to the sites on possible data entry errors. These inconsistencies as well as other discrepancies in the eCRF data identified during data cleaning or monitoring of data will be queried to the investigators via the EDC system. Answers to queries or changes of the data will directly be documented in the system. After all data are entered, all queries are solved, and all external data (e.g. data from the central laboratory) have been received and reconciled, the database will be closed.

Data generated by this study must be available for inspection by representatives of other national and local health authorities, the Sponsor, and the IEC for each study site, if appropriate.

Participants will be identified on eCRFs and other documents submitted to the Sponsor or organisations working on behalf of the Sponsor by their subject number, not by name or initials. Documents not to be submitted to the Sponsor or organisations working on behalf of the Sponsor that identify the subject (e.g., the signed informed consent) must be maintained in confidence by the Investigator.

12.2 Monitoring and Quality Assurance

Clinical trial management and monitoring will be performed by NBCD with the assistance of a local CRA in Greece. In order to ensure the clinical trial is managed and conducted properly NBCD will:

- Set up workflows, timelines, project specific procedures and tracking tools;
- Monitor timelines and milestones on an ongoing basis and communicate the project status to the project team throughout the duration of the entire project;
- Conduct regular team meetings to discuss project status, activities, address issues, coordination of project specific training;
- Maintain regular contact with principal investigators;
- Provide status reports to PI, regular site contacts, back-up according to agreed project structures;
- Provide periodic internal study status reports to key staff;
- Maintain files according to the SOPs of NBCD
- Supporting the study sites with protocol and study-related questions;

- Initiate actions to address project issues.

During the study, the monitor/clinical research associate (CRA) will visit the investigational site regularly to check the completeness of patient records, the accuracy of entries in the eCRF, the adherence to the protocol and to GCP, the progress of enrolment, and to ensure that study drug is being stored, dispensed, and accounted for according to specifications. Key trial personnel must be available to assist the monitor during these visits. The investigator must maintain source documents for each participant in the study, consisting of case and visit notes (hospital or clinic medical records) containing demographic and medical information, laboratory data and the results of any other tests or assessments. All information on eCRFs must be traceable to these source documents in the patient's file. The investigator must also keep the original of the signed informed consent form. The investigator must give the monitor access to all relevant source documents to confirm their consistency with the CRF entries. Specific monitoring requirements will be described in the study specific monitoring guideline provided to the CRA prior to his/her first site visit.

12.3 Amendments

A 'substantial amendment' is defined as an amendment to the terms of the IEC application, or to the protocol or any other supporting documentation, that is likely to affect to a significant degree:

- the safety or physical or mental integrity of the participants of the trial;
- the scientific value of the trial;
- the conduct or management of the trial; or
- the quality or safety of any intervention used in the trial.

All substantial amendments will be notified to the IEC and to the CA.

Non-substantial amendments will not be notified to the IEC and the CA but will be recorded and filed by the Sponsor.

12.4 Annual progress report

The Sponsor/PI/delegated person will submit a summary of the progress of the trial to the IEC once a year in accordance with local requirements. Information will be provided on the date of inclusion of the first participant, numbers of participants included and numbers of participants that have completed the trial, SAEs/SARs, other problems, and amendments.

12.5 Temporary halt and (prematurely) end of study report

The Sponsor will notify the IEC and the CA of the end of the study within a period of 90 days. The end of the study is defined as the last patient's last visit.

The Sponsor will notify the IEC and the CA immediately of a temporary halt of the study, including the reason of such an action. In case the study is ended prematurely, the Sponsor will notify the IEC and the CA within 15 days, including the reasons for the premature termination. Within one year after the end of the study, the investigator/Sponsor will submit a final study report with the results of the study, including any publications/abstracts of the study, to the IEC and the CA.

12.6 Public disclosure and publication policy

The final report will be written in English in a Word format, and its structure will follow a template based upon the ICH E3, guidelines unless otherwise specified by the Sponsor in the financial agreement.

Suggested inclusions in the report are: study objectives, materials and methods (including any deviations from the study protocol), evaluation of the study results, observations by the investigator as to the value of the study drug per se, and a discussion of all adverse experiences with interpretation by the investigator as to the study drug involvement.

All information concerning the tested drug and the Sponsor's operation, such as patent applications, formulae, manufacturing processes, basic scientific data and formulation information supplied by the Sponsor and not previously published are considered confidential and shall remain the sole property of the Sponsor. The investigator agrees to use this information only in accomplishing this study and will not use it for other purposes without written consent from the Sponsor.

It is understood by the investigator that the information from the clinical study will be used by the Sponsor in connection with the development of the tested drug and, therefore, may be disclosed as required to other clinical Investigators or government agencies. In order to allow for the use of the information derived from the clinical studies, it is understood that there is an obligation to provide the Sponsor with complete test results and all data developed in the study.

The trial drug and the information in this document, and in any future information supplied, contain trade secrets and commercial information that are privileged or confidential and may not be disclosed unless such disclosure is required by law or regulations.

In any event, persons to whom the information is disclosed must be informed that the information is privileged or confidential and may not be further disclosed by them.

Publication rules will be consistent with local regulation and will be addressed in the study contract and should not be in contradiction with the text in the protocol.

Furthermore, a trial publication policy will be formulated by the REGAIN Consortium.

13 REFERENCES

Bermingham, N. A., Hassan, B. A., Price, S. D., Vollrath, M. A., Ben-Arie, N., Eatock, R. A., Zoghbi, H. Y. (1999). Math1: An essential gene for the generation of inner ear hair cells. *Science*, *284*(5421), 1837-41.

Bramhall, N. F., Shi, F., Arnold, K., Hochedlinger, K., & Edge, A. S. (2014). Lgr5-positive supporting cells generate new hair cells in the postnatal cochlea. *Stem Cell Reports*, *2*(3), 311-22. doi:10.1016/j.stemcr.2014.01.008

Daudet, N., & Lewis, J. (2005). Two contrasting roles for notch activity in chick inner ear development: Specification of prosensory patches and lateral inhibition of hair-cell differentiation. *Development (Cambridge, England)*, *132*(3), 541-51. doi:10.1242/dev.01589

Doody, R. S., Raman, R., Farlow, M., Iwatsubo, T., Vellas, B., Joffe, S., . . . Semagacestat Study Group. (2013). A phase 3 trial of semagacestat for treatment of alzheimer's disease. *The New England Journal of Medicine*, *369*(4), 341-50. doi:10.1056/NEJMoa1210951

Edge, A. S., & Chen, Z. Y. (2008). Hair cell regeneration. *Current Opinion in Neurobiology*, *18*(4), 377-82. doi:10.1016/j.conb.2008.10.001

El Sabbagh, N. G., Sewitch, M. J., Bezdjian, A., & Daniel, S. J. (2016). Intratympanic dexamethasone in sudden sensorineural hearing loss: A systematic review and meta-analysis. *The Laryngoscope.* doi:10.1002/lary.26394

Gubbels, S. P., Woessner, D. W., Mitchell, J. C., Ricci, A. J., & Brigande, J. V. (2008). Functional auditory hair cells produced in the mammalian cochlea by in utero gene transfer. *Nature*, *455*(7212), 537-41. doi:10.1038/nature07265

Hansen, A. R., Graham, D. M., Pond, G. R., & Siu, L. L. (2014). Phase 1 trial design: Is 3+ 3 the best? *Cancer Control: Journal of the Moffitt Cancer Center*, *21*(3), 200. Retrieved from Google Scholar.

Henley, D. B., Sundell, K. L., Sethuraman, G., Dowsett, S. A., & May, P. C. (2014). Safety profile of semagacestat, a gamma-secretase inhibitor: IDENTITY trial findings. *Current Medical Research and Opinion*, *30*(10), 2021-32. doi:10.1185/03007995.2014.939167

Hong, O., Kerr, M. J., Poling, G. L., & Dhar, S. (2013). Understanding and preventing noise-induced hearing loss. *Disease-a-month : DM*, *59*(4), 110-8. doi:10.1016/j.disamonth.2013.01.002

Hori, R., Nakagawa, T., Sakamoto, T., Matsuoka, Y., Takebayashi, S., & Ito, J. (2007). Pharmacological inhibition of notch signaling in the mature guinea pig cochlea. *Neuroreport*, *18*(18), 1911-1914. Retrieved from Google Scholar.

Izumikawa, M., Minoda, R., Kawamoto, K., Abrashkin, K. A., Swiderski, D. L., Dolan, D. F., Raphael, Y. (2005). Auditory hair cell replacement and hearing improvement by atoh1 gene therapy in deaf mammals. *Nature Medicine*, *11*(3), 271-6. doi:10.1038/nm1193

Jeon, S. J., Fujioka, M., Kim, S. C., & Edge, A. S. (2011). Notch signaling alters sensory or neuronal cell fate specification of inner ear stem cells. *The Journal of Neuroscience : The Official Journal of the Society for Neuroscience*, *31*(23), 8351-8. doi:10.1523/JNEUROSCI.6366-10.2011

Juan, I. D., & Linthicum, F. H. (2010). Round window fibrous plugs. *Otology & Neurotology : Official Publication of the American Otological Society, American Neurotology Society [and] European Academy of Otology and Neurotology*, *31*(8), 1354-5. doi:10.1097/MAO.0b013e3181c4c2bb

Kelley, M. W. (2006). Regulation of cell fate in the sensory epithelia of the inner ear. *Nature Reviews. Neuroscience*, *7*(11), 837-49. doi:10.1038/nrn1987

Knudsen, L. V., Oberg, M., Nielsen, C., Naylor, G., & Kramer, S. E. (2010). Factors influencing help seeking, hearing aid uptake, hearing aid use and satisfaction with hearing aids: A review of the literature. *Trends in Amplification*, *14*(3), 127-54. doi:10.1177/1084713810385712

Lin, F. R. (2012). Hearing loss in older adults: Who's listening? *JAMA*, *307*(11), 1147-8. doi:10.1001/jama.2012.321

McCormack, A., & Fortnum, H. (2013). Why do people fitted with hearing aids not wear them? *International Journal of Audiology*, *52*(5), 360-8. doi:10.3109/14992027.2013.769066

Merchant, S. N., Adams, J. C., & Nadol, J. B. (2005). Pathology and pathophysiology of idiopathic sudden sensorineural hearing loss. *Otology & Neurotology : Official Publication of the American Otological Society, American Neurotology Society [and] European Academy of Otology and Neurotology*, *26*(2), 151-60.

Mizutari, K., Fujioka, M., Hosoya, M., Bramhall, N., Okano, H. J., Okano, H., & Edge, A. S. (2013). Notch inhibition induces cochlear hair cell regeneration and recovery of hearing after acoustic trauma. *Neuron*, *77*(1), 58-69. doi:10.1016/j.neuron.2012.10.032

Müller, U., & Barr-Gillespie, P. G. (2015). New treatment options for hearing loss. *Nature Reviews. Drug Discovery*, *14*(5), 346-65. doi:10.1038/nrd4533

Panza, F., Frisardi, V., Imbimbo, B. P., Capurso, C., Logroscino, G., Sancarlo, D., . . . Solfrizzi, V. (2010). REVIEW: Γ-Secretase inhibitors for the treatment of alzheimer's disease: The current state. *CNS Neuroscience & Therapeutics*, *16*(5), 272-84. doi:10.1111/j.1755-5949.2010.00164.x

Rubel, E. W., Furrer, S. A., & Stone, J. S. (2013). A brief history of hair cell regeneration research and speculations on the future. *Hearing Research*, *297*, 42-51. doi:10.1016/j.heares.2012.12.014

Siemers, E., Skinner, M., Dean, R. A., Gonzales, C., Satterwhite, J., Farlow, M., . . . May, P. C. (2005). Safety, tolerability, and changes in amyloid beta concentrations after administration of a gamma-secretase inhibitor in volunteers. *Clinical Neuropharmacology*, *28*(3), 126-32.

Siemers, E. R., Quinn, J. F., Kaye, J., Farlow, M. R., Porsteinsson, A., Tariot, P., . . . Knopman, D. S. (2006). Effects of a gamma-secretase inhibitor in a randomized study

of patients with alzheimer disease. *Neurology*, *66*(4), 602. Retrieved from Google Scholar.

Stone, J. S., & Rubel, E. W. (2000). Temporal, spatial, and morphologic features of hair cell regeneration in the avian basilar papilla. *The Journal of Comparative Neurology*, *417*(1), 1-16.

De Strooper, B., Vassar, R., & Golde, T. (2010). The secretases: Enzymes with therapeutic potential in alzheimer disease. *Nature Reviews. Neurology*, *6*(2), 99-107. doi:10.1038/nrneurol.2009.218

Suckfuell, M., Lisowska, G., Domka, W., Kabacinska, A., Morawski, K., Bodlaj, R., . . . Meyer, T. (2014). Efficacy and safety of AM-111 in the treatment of acute sensorineural hearing loss: A double-blind, randomized, placebo-controlled phase II study. *Otology & Neurotology : Official Publication of the American Otological Society, American Neurotology Society [and] European Academy of Otology and Neurotology*, *35*(8), 1317-26. doi:10.1097/MAO.0000000000000466

A Three-part, Multicenter, Open Label, Single Dose Study to Assess the Safety, Tolerability, and Efficacy of Intra Labyrinthine (IL) CGF166 in Patients with Severe-to-profound Hearing Loss. (n.d.). A three-part, multicenter, open label, single dose study to assess the safety, tolerability, and efficacy of intra labyrinthine (IL) CGF166 in patients with severe-to-profound hearing loss. [Web page]. Retrieved from https://clinicaltrials.gov/ct2/show/study/NCT02132130?term=CFG166&rank=1

Tona, Y., Hamaguchi, K., Ishikawa, M., Miyoshi, T., Yamamoto, N., Yamahara, K., . . . Nakagawa, T. (2014). Therapeutic potential of a gamma-secretase inhibitor for hearing restoration in a guinea pig model with noise-induced hearing loss. *BMC Neuroscience*, *15*, 66. doi:10.1186/1471-2202-15-66

Vio, M. M., & Holme, R. H. (2005). Hearing loss and tinnitus: 250 million people and a US 10 billion potential market. *Drug Discovery Today*, *10*(19), 1263. Retrieved from Google Scholar.

Yamamoto, N., Tanigaki, K., Tsuji, M., Yabe, D., Ito, J., & Honjo, T. (2006). Inhibition of notch/RBP-J signaling induces hair cell formation in neonate mouse cochleas. *Journal of Molecular Medicine (Berlin, Germany)*, *84*(1), 37-45. doi:10.1007/s00109-005-0706-9

Yuan, X., Wu, H., Xu, H., Xiong, H., Chu, Q., Yu, S., . . . Wu, K. (2015). Notch signaling: An emerging therapeutic target for cancer treatment. *Cancer Letters*, *369*(1), 20-27. Retrieved from Google Scholar.

Zhao, L. D., Guo, W. W., Lin, C., Li, L. X., Sun, J. H., Wu, N., . . . Yang, S. M. (2011). Effects of DAPT and atoh1 overexpression on hair cell production and hair bundle orientation in cultured organ of corti from neonatal rats. *PloS One*, *6*(10), e23729. doi:10.1371/journal.pone.0023729

Zheng, J. L., & Gao, W. Q. (2000). Overexpression of math1 induces robust production of extra hair cells in postnatal rat inner ears. *Nature Neuroscience*, *3*(6), 580-6. doi:10.1038/75753

APPENDIX A

Definition of women of childbearing potential and of fertile men
A woman is considered of childbearing potential, following menarche and until becoming post-menopausal unless permanently sterile. Permanent sterilisation methods include hysterectomy, bilateral salpingectomy and bilateral oophorectomy. A postmenopausal state is defined as no menses for 12 months without an alternative medical cause.

A man is considered fertile after puberty unless permanently sterile by bilateral orchidectomy.

Birth control methods which may be considered as highly effective
Methods that can achieve a failure rate of less than 1% per year when used consistently and correctly are considered as highly effective birth control methods. Such methods include:

- combined (estrogen and progestogen containing) hormonal contraception associated with inhibition of ovulation:
  - oral
  - intravaginal
  - transdermal
- progestogen-only hormonal contraception associated with inhibition of ovulation 1:
  - oral
  - injectable
  - implantable
- intrauterine device
- intrauterine hormone-releasing system
- bilateral tubal occlusion
- vasectomised partner
- sexual abstinence (sexual abstinence is considered a highly effective method only if defined as refraining from heterosexual intercourse during the entire period of risk associated with the study treatments. The reliability of sexual abstinence needs to be evaluated in relation to the duration of the clinical trial and the preferred and usual lifestyle of the subject.)
