# Supplementary material for: A phase I/IIa safety and efficacy trial of intratympanic gamma-secretase inhibitor as a regenerative drug treatment for sensorineural hearing loss
Source: Nat Commun. 2024 Mar 1;15:1896. doi: 10.1038/s41467-024-45784-0 (PMC10907343; doi:10.1038/s41467-024-45784-0)
Supplement: Supplementary file 6 — Supplementary Data 3 [file 41467_2024_45784_MOESM6_ESM.pdf]

Statistical Analysis Plan

Protocol identification No.:	AUT-001
Protocol version:	5.0
Protocol date:	04-Dec-2018
EudraCT number:	2016-004544-10
Study title:	A Phase I/II multiple ascending dose open-label safety and efficacy study of the Notch Inhibitor LY3056480 in patients with mild to moderate sensorineural hearing loss.
Investigational product:	LY3056480
Development phase:	II (part B)
Sponsor:	Audion Therapeutics BV<br>Linnaeusparkweg 10-2<br>1098 EA, Amsterdam<br>Netherlands
Coordinating investigator	Prof Anne Schilder<br>NIHR Research Professor, Professor of Paediatric Otorhinolaryngology & Director evidENT, UCL
Author of SAP:	Yesilda Balavarca<br>Senior Biostatistician<br>Staburo GmbH<br>Aschauer Straße 26b<br>81549 München, Germany
SAP version / status:	Final v02
Date of SAP:	07-Aug-2020

CONFIDENTIAL: May not – in full or in part – be passed on, be reproduced, published or otherwise used without the express permission of the sponsor.

1 Version Control

Version	Date	Section	Description
Final v01	31-Oct-2019	ALL	Initial release
Final v02	07-Aug-2020	Section 9.7.3.4	In page 20, a typo mistake “12 months” instead of “12 weeks” has been corrected.
		Section 9.7.3.18	This section is now developed in section 9.7.4.1.
		Section 9.7.4.1	New section to describe the efficacy analysis for follow-up of endpoints at 6 and 12 months.
		Section 9.7.5	Corrected to consider the pooled analysis of speech audiometry with the method of Words in babble.

2 Statistical Analysis Plan Approval

Author approval:

Biostatistician<br>Staburo GmbH<br>Aschauerstr. 26b<br>81549 Munich, Germany		
13 AUG 2020	Yesilda Balavarca	[signature]
Date (dd/mmm/yyyy)	Name	Signature

Statistics approval:

Biostatistician<br>Staburo GmbH<br>Aschauerstr. 26b<br>81549 Munich, Germany		
13AUG2020	Laura Schlieker	[signature]
Date (dd/mmm/yyyy)	Name	Signature

Sponsor approval:

Sponsor or legal representative<br>Audion Therapeutics BV<br>Hogeweg 54<br>1098 CE, Amsterdam, Netherlands		
13AUG2020	Rolf Jan Rutten, CEO	[signature]
Date (dd/mmm/yyyy)	Name	Signature

Clinical Investigator approval:

National Kapodistrian University of Athens<br>1st Department of Otorhinolaryngology – Head & Neck Surgery, National and Kapodistrian University of Athens, Greece<br>Vasilissis Sofias 114, 115 27, Athens, Greece	Athanasios Bibas	
Date (dd/mmm/yyyy)	Name	Signature

Investigator approval:

National Kapodistrian University of Athens<br>1st Department of Otorhinolaryngology – Head & Neck Surgery, National and Kapodistrian University of Athens, Greece<br>Vasilissis Sofias 114, 115 27, Athens, Greece	Konstantinos Pastiadis	
Date (dd/mmm/yyyy)	Name	Signature

Clinical Investigator approval:

National Kapodistrian University of Athens<br>1st Department of Otorhinolaryngology – Head & Neck Surgery, National and Kapodistrian University of Athens, Greece<br>Vasilissis Sofias 114, 115 27, Athens, Greece	Athanasios Bibas	
Date (dd/mmm/yyyy)	Name	Signature

Investigator approval:

National Kapodistrian University of Athens<br>1st Department of Otorhinolaryngology – Head & Neck Surgery, National and Kapodistrian University of Athens, Greece<br>Vasilissis Sofias 114, 115 27, Athens, Greece	Konstantinos Pastiadis	
Date (dd/mmm/yyyy)	Name	Signature

3 Table of Contents

1 Version Control....2
2 Statistical Analysis Plan Approval....3
3 Table of Contents....5
4 List of Abbreviations....7
5 Introduction....8
6 Study Objectives and Endpoints....9
6.1 Study Objectives....9
6.1.1 Primary objective....9
6.1.2 Secondary objectives....9
6.2 Endpoints....9
6.2.1 Primary Efficacy Endpoint....9
6.2.2 Secondary Efficacy Endpoints....9
6.2.3 Safety and tolerability Endpoints....10
6.2.4 Further Endpoints....10
7 Study Methods....11
7.1 General Study Design and Plan....11
7.2 Inclusion and Exclusion Criteria....11
7.3 Investigational Product / Study Discontinuation....11
7.4 Randomisation and Blinding....11
7.5 Sample Size....11
7.6 Interim Analysis....12
7.7 Pooling of Centres....12
8 General Considerations Pertaining to the Statistical Analysis....13
8.1 Analysis Sets....13
8.1.1 Analysis Sets for Efficacy Analyses....13
8.1.2 Analysis Sets for Safety Analyses....13
8.2 Missing Data....13
8.3 Baseline Definition....14
The baseline value is defined as the last value prior to first dose of study treatment.....14
9 Statistical Analysis....15
9.1 Specifications Related to the Whole Analysis....15

9.1.1 Analysis Sets ... 15
9.1.2 Listings ... 15
9.1.3 Tables ... 15
9.2 Patient Disposition ... 15
9.3 Demographics and Baseline Characteristics ... 16
9.4 Medical/Surgical History ... 16
9.5 Previous and Concomitant Medication ... 16
9.6 Compliance ... 16
9.7 Efficacy Analyses ... 16
9.7.1 Descriptive Analyses of Efficacy Endpoint ... 16
9.7.2 Primary Efficacy Analysis ... 18
9.7.3 Secondary Analyses ... 19
9.7.4 Exploratory Efficacy Analyses ... 23
9.7.5 Subgroup Analyses ... 24
9.8 Safety Analysis ... 24
9.8.1 Physical Examination ... 24
9.8.2 Vital Signs ... 24
9.8.3 ECG ... 24
9.8.4 Laboratory measurements ... 24
9.8.5 Adverse Events ... 25
10 Reporting Conventions ... 26
11 Technical Details ... 27
12 Summary of Changes to the Protocol ... 28
13 Contents of Tables, Figures and Listings ... 29
14 References ... 30

4 List of Abbreviations

AE	Adverse Event
ART	Acoustic Reflex Test
(e)CRF	(Electronic) Case Report Form
dB	Decibel
dBHL	Decibels Hearing Level
DHI	Dizziness Handicap Inventory
DPOAE	Distortion Product Oto-Acoustic Emissions
ECG	Electrocardiogram
EudraCT	European drug regulatory affairs Clinical Trials
GCP	Good Clinical Practice
HHIA/E	Hearing Handicap Inventory Adults / Elderly
HHS	Hearing Handicap Score
ICH	International Conference on Harmonisation
IMP	Investigational Medicinal Product
(m)ITT	(Modified) Intention-to-Treat
kHz	Kilohertz
PI	Principal Investigator
PTA	Pure-Tone Audiometry
SAE	Serious adverse event
SAP	Statistical Analysis Plan
SiN	Speech in Noise
SNHL	Sensorineural Hearing Loss
SNR	Signal to Noise Ratio
SOP	Standard Operating Procedure
Sponsor	See definition in Section 1.53 of ICH-GCP
SUSAR	Suspected Unexpected Serious Adverse Reaction
TEN	Threshold-Equalizing-Noise
TFI	Tinnitus Function Test
UCL(H)	University College London (Hospital)
UK	United Kingdom
VNG	Videonystagmography
WiB	Words in Babble

5 Introduction

This document presents the Statistical Analysis Plan (SAP) for study REGAIN – part B based on the clinical study protocol version 5.0 from 04 December 2018 (hereinafter referred to as “the protocol”). Pertinent excerpts from the protocol will be given as enquoted italicised text wherever appropriate.

The SAP follows the principles of the Guidelines ICH (International Conference on Harmonisation of Technical Requirements for Registration of Pharmaceuticals for Human Use) Topic E3 [1] and ICH Topic E9 [2]. It gives all details for the final statistical analysis of this study.

The present study is a multi-centre efficacy phase II study in adult volunteers with mild to moderate sensorineural hearing loss (SNHL). The study is designed to establish efficacy parameters at 250µg of the investigational medicinal product (IMP) LY3056480.

6 Study Objectives and Endpoints

6.1 Study Objectives

Protocol Section 3:

The aim of REGAIN Part B is to establish the efficacy of local treatment with LY3056480 in patients with mild to moderate SNHL.

The objectives are:

6.1.1 Primary objective

1. *To establish the efficacy of local treatment with LY3056480 in terms of hearing at 12 weeks.*

6.1.2 Secondary objectives

2. *To establish the efficacy of local treatment with LY3056480 in terms of hearing at 6 weeks*
3. *To continue to assess the safety and tolerance of local treatment with LY3056480 in multiple doses, in terms of:*
   a) Occurrence and severity of IMP-related local and systemic adverse events (*AEs)*
   b) Occurrence and severity of procedure related local and systemic AEs

6.2 Endpoints

Protocol Section 4.3:

6.2.1 Primary Efficacy Endpoint

Average change in hearing from baseline in the treated ear at 12 weeks across three frequencies (2, 4, 8 kHz), as measured by Pure-Tone Audiometry (*PTA) (*Decibels Hearing Loss (*dBHL)).*

6.2.2 Secondary Efficacy Endpoints

- *Hearing: Change from baseline at 6 and 12 weeks (treated ear, untreated ear and difference), in terms of:*
  - Hearing level as tested by PTA (dBHL) at individual frequencies (0.25, 0.5, 1, 2, 3, 4, 6, 8, 12.5 and 16 kHz).
  - Average change in hearing level across three frequencies (2, 4, 8 kHz), as measured by PTA (dBHL) (at 6 weeks only).
  - Speech audiometry as tested by speech in noise testing to determine signal to noise ratio (SNR) *loss shift.*

- *Middle ear immittance as tested by tympanometry and* Acoustic Reflex Test *(ART) to determine middle ear pressure, volume and compliance values and acoustic threshold reflex shift.*
- Distortion Product Oto-Acoustic Emissions (*DPOAE) - SNR and absolute levels.*
- *Cochlear dead regions as tested by the Threshold Equalising Noise test* (TEN).
- *Hearing specific quality of life (per patient), as measured by the* Hearing Handicap Inventory Adults/Elderly (*HHIA/E) questionnaire.*
- *Level of tinnitus as measured by the* tinnitus function test *(TFI).*
- *Change in Hearing Aid use as measured by the Hearing Aid Outcome Questionnaire (at month 6 and 12 optional visits).*

• *Balance: Change from baseline at 12 weeks, as measured by a clinical balance assessment*
- *including History and Examination (Eye Movements, Head Thrust, modified Romberg, Unterberger, Bithermal Air Calorics using* Videonystagmography *(VNG)), and Dizziness Handicap Inventory.*

6.2.3 Safety and tolerability Endpoints

• *Hearing and balance as defined in the above endpoints.*
• *Facial nerve function: Change from baseline at the treated side up to week 12, in terms of:*
- *Facial nerve function as measured by the House-Brackman grading scale.*
- *Taste, as reported by the participant (no change, altered taste, loss of taste).*

• *Occurrence and severity of IMP-related local and systemic AEs up to 12 weeks.*
• *Occurrence and severity of procedure related local and systemic AEs up to 12 weeks.*
• *Occurrence of systemic AEs as measured by potentially clinically significant changes in* Electrocardiogram (*ECG), vital signs, physical examinations and laboratory tests up to 12 weeks.*
• *Occurrence of injection sites reactions in and around the treated ear as assessed by otomicroscopy up to 12 weeks.*

6.2.4 Further Endpoints

<u>Protocol Section 10.9:</u>

• *Disposition of participants and discontinuations.*
• *Demographics and other baseline characteristics.*
• *Treatment exposure as assessed by the number of doses received.*
• *Medical history and surgical history.*
• Other exploratory and/or safety endpoints of Facial nerve function and Taste.

7 Study Methods

7.1 General Study Design and Plan

For a more detailed description of study design and procedures than the brief abstract given below, refer to Section 4.1 and to schedule of assessments in Table 4 of the protocol.

Protocol Section 4, 4.1:

REGAIN Part B is a multi-centre efficacy study with patients with mild to moderate SNHL *treated with 250 µg of LY3056480.*

The worst hearing ear according to PTA and/or speech audiometry will be treated. Upon enrollment, the first dose of study drug will be administered trans-tympanically by the principal investigator (*PI) on Day 1. Subsequent doses will be administered at Day 8 and 15.*

Participants will be treated for 2 weeks and then followed for safety and efficacy assessment for an additional 10 weeks. Additional follow up visits at month 6 and 12 are optional.

7.2 Inclusion and Exclusion Criteria

See Sections 5.2 and 5.3 of the protocol for a detailed description of the inclusion and exclusion criteria.

Male or female patients aged 18 – 80 years, who meet all inclusion criteria, will be included. Patients with certain prior or concomitant illnesses, medications and procedures are excluded to limit confounding factors that may complicate the interpretation of the study results and to protect patients whose safety could be compromised by participation in the study.

7.3 Investigational Product / Study Discontinuation

For description of the investigational product refer to Section 7.1 of the protocol. For details on study discontinuation refer to Section 8.4 and 8.7 of the protocol.

7.4 Randomisation and Blinding

The present study is a non-randomized and non-blinded study.

7.5 Sample Size

A total of 40 evaluable patients are aimed to complete the study. For a two-sided test and a significance level of 5%, this size relates to a power of 87% to detect an improvement in hearing corresponding to an effect size of 0.5.

Details on statistical power calculations are provided in Section 10.2 of the clinical protocol.

Patients who are not evaluable may be replaced to increase the chance of having at least 40 patients completing the study and entering in the main statistical analysis.

7.6 Interim Analysis

No interim analysis will be conducted.

7.7 Pooling of Centres

All statistical analyses will be performed on pooled patients from all centres.

In addition, per centre analyses will be considered for the following endpoints:

Pure-Tone Audiometry (PTA): Due to differences across sites in procedures to measure this primary endpoint, additional analysis per site is needed.

Speech in noise (QuickSiN and WiB): Due to differences across sites in conducting speech in noise (difference in method and language), a pooled analysis would not be appropriate for this secondary endpoint. For this reason, speech in noise is evaluated per site. In an effort to streamline tests across sites the German site implemented the QuickSiN with sentences from the standard German test (the OLSA). We found that the results from this German QuickSiN are not comparable to those used in the UK as the OLSA sentences result are too easy to interpret in the QuickSiN format. Therefore, it is proposed that instead of using the German QuickSiN data we will use the OLSA data in a post-hoc analysis.

8 General Considerations Pertaining to the Statistical Analysis

8.1 Analysis Sets

Protocol Section 10.3:

8.1.1 Analysis Sets for Efficacy Analyses

All analyses will be based on planned treatment regimen rather than the actual treatment given in case of any differences. The following analysis sets are defined:

Intention-to-treat analysis set (full analysis set)
The ITT analysis set will include all eligible *participants.*

Modified intention-to-treat analysis set
The mITT analysis set will include participants from the ITT analysis set who have a baseline and at least one post-dose PTA assessment available.

Per-protocol analysis set
The per-protocol analysis set will include all participants from the mITT analysis set who have been treated according to the study protocol and fulfil the following criteria:

- *Compliance with all entry criteria.*
- *Absence of major protocol deviations with respect to factors likely to affect the efficacy of treatment.*
- *Adequate treatment compliance, defined as two or more doses of the study drug.*

8.1.2 Analysis Sets for Safety Analyses

Safety analysis set
The safety analysis set will include all eligible *participants undergoing receiving at least one* (out of three) *dose of study treatment.*

8.2 Missing Data

Protocol Section 10.4:

As a general rule, all data collected and available will be used in the analysis. No imputation of missing data will be applied independently of the statistical methods used to analyse the data.
Handling of missing data from questionnaires will depend on whether only single items are missing or a complete questionnaire is missing. If only single items are missing, the rules defined by the authors of the questionnaires (if any) will be followed.

Rules for generation of total score and subscale scores with missing response items for endpoint TFI are available in the respective questionnaire for tinnitus functional index. Briefly, for the total TFI score to be valid, the respondent must answer at least 19 items (out of 25). For subscale scores to be valid, no more than 1 item (out of 3 or 4) should be omitted.

8.3 Baseline Definition

The baseline value is defined as the last value prior to first dose of study treatment.

9 Statistical Analysis

The final statistical analysis will be performed after data base lock.

9.1 Specifications Related to the Whole Analysis

9.1.1 Analysis Sets

The primary and secondary endpoint analyses will be performed using the mITT population. Unless otherwise specified, all analyses which are conducted using the mITT set will be repeated using the per-protocol set, if the two analysis sets differ (e.g., ≥ 10% difference in patients belonging to the sets).

9.1.2 Listings

Data in the electronic Case Report Form (eCRF) as well as all relevant generated and transformed variables (if any) will be listed. Unless otherwise specified, treatment variable (treated/untreated ear) will be included in listings when appropriate, and listings will be sorted by patient identifier and then by visit number and/or a relevant date (e.g. date of onset of AE).

9.1.3 Tables

Descriptive summary tables will be grouped by treatment (e.g. for treated/untreated ear, when it applies) and visit as appropriate.

For quantitative data, number of patients, number of missing values, mean, standard deviation, minimum, first quartile, median, third quartile, and maximum will be shown. Categorical data will be displayed in frequency tables showing the number of patients, number of missing values, absolute frequencies, and relative frequencies (in %) of patients in each category.

Abbreviations will not be displayed without any explanations. They will be either spelled out in the table or explained in footnotes (whatever will be more reasonable from programming point of view).

9.2 Patient Disposition

Patients in the ITT analysis set will be listed with name of study site, date of informed consent, eligibility status, visit dates, study completion status, occurrence of temporary and/or permanent investigational product discontinuation, and reasons for study or product discontinuation (if applicable). Ineligible patients will be listed with pertinent inclusion and/or exclusion criteria. For patients who provide informed consent and are not included into the study, the reason for withdrawal will also be recorded, if given. Protocol deviations will be listed if available in deviation logs or the clinical database.

Assignment of patients of the ITT set to the sets mITT, per-protocol and safety, and the reasons for exclusion from any of these analysis sets, if available, will be listed with name of study site.

Visit completion status will be summarised by phase visit for the mITT set.

9.3 Demographics and Baseline Characteristics

Name of study site, demographics (date of birth, race, height, weight, smoking history, and socio economic status), and baseline characteristics (e.g. cause and duration of hearing loss) will be listed and summarised for the ITT set and for the safety set, if the two sets differ (e.g., ≥ 10% difference in patients belonging to the sets).

9.4 Medical/Surgical History

Medical history and surgical history will be listed with date of onset, date of resolution, and treatment (if available) for the ITT set. If a coding is available, then coded entities will each be summarised overall for the ITT.

9.5 Previous and Concomitant Medication

Previous and concomitant medications will be listed with date of screening, indication, start date, stop date, dose, frequency, form, and route for the ITT set. If a coding is available, then coded previous medication and coded concomitant medication entities will separately be summarised for the ITT set and safety set, if the two sets differ.

9.6 Compliance

Treatment compliance with respect to the whole doses will be listed and summarised for the ITT set, if data is available.

9.7 Efficacy Analyses

Efficacy analyses will be done using the mITT population. All tests, including the test of the primary efficacy endpoint, will be two-sided at a significance level of 5%. All confidence intervals will be two-sided at 95% confidence level. Adjustments for multiplicity will not be applied due to the exploratory characteristic of this study.

9.7.1 Descriptive Analyses of Efficacy Endpoint

9.7.1.1 PTA – primary endpoint

For the quantitative efficacy PTA endpoint, baseline measurements (using the baseline definition given in Section 8.3) and measurements at 6 and 12 weeks will be listed by patient and by frequency (in kHz), for the treated and untreated ear. An analogous listing will be produced using baseline measurements and changes from baseline to week 6 and week 12.

Clinically relevant improvements in hearing level, which is defined by a decrease of at least 10dB (i.e. change ≤ -10 dB), will be identified. The number of improvements (responses) per patient will be counted across frequencies, and by time point. These summaries will be produced for the treated and untreated ear.

Further, changes in treated ear versus changes in untreated ear will be calculated, and the number of improvements in the treated over the untreated ear will be summarized across frequencies, by time point for each patient.

The following steps summarize the abovementioned calculations of changes in PTA level for each patient:

Treated and untreated ear, separately:

1. $Change_{0\text{-}12}$ = (PTA_{12} – PTA_0); for changes from 0 to 12 weeks
2. If $change_{0\text{-}12}$ ≤ -10, then response=1
3. Repeat 1 and 2 for every frequency kHz.
4. $Response_{12}$ = {number of times when response=1, across all frequencies}
5. Similarly, repeat 1 to 4 to calculate $Response_6$, related to changes from 0 to 6 weeks.

Treated versus untreated ear:

1. $Diff\_change_{0\text{-}12}$ = $(PTA_{12} – PTA_0)_T$ - $(PTA_{12} – PTA_0)_U$
2. If $diff\_change_{0\text{-}12}$ ≤ -10, then response=1
3. Repeat 1 and 2 for every frequency kHz.
4. $Response_{12,T/U}$ = {number of times when response=1, across all frequencies}
5. Similarly, repeat 1 to 4 to calculate $Response_{6,T/U}$, related to diff_change from 0 to 6 weeks.

Graphical displays will include:

A. Line plots (Audiogram) of individual patient's hearing level (in dB) against frequencies (in kHz) by time points at baseline, 6 weeks, and 12 weeks, for the treated ear.

B. Line plots for all patients or at least for patients showing response ≥1 in any post-treatment change from baseline.

B1. Line plots of individual patient's hearing level (in dB) against time points at baseline, 6 weeks, and 12 weeks, per frequencies (in kHz), for the treated ear.

B2. Line plots of individual differences in change between treated and untreated ear across frequencies (in kHz), and against time points at baseline, 6 weeks and 12 weeks.

Alternatively, if line plots in B1 or B2 are not clearly showing the individual changes, bar graphs will be used as complementary display.

C. Summary of patient's hearing level, by PTA domain (average of high and low frequencies):

C1. Summary boxplots of patient's hearing level in treated ear at two frequency groups, in average PTA at 2, 4, 8 kHz (PTA high frequencies) and 0.25, 0.5, 1 kHz (PTA low frequencies), at baseline, 6 weeks and 12 weeks.

C2. Summary boxplots of patient's hearing level in untreated ear, similar as in C1.

C3. Summary boxplots of difference in patient's hearing level between treated and untreated ear, similar as in C1.

C4. Summary bar graphs of difference in changes from baseline to 6 and 12 weeks, between treated and untreated ear, in average PTA at 2, 4, 8 kHz (PTA high frequencies) and 0.25, 0.5, 1 kHz (PTA low frequencies).

D. Summary of patient's hearing level, by PTA frequency:

D1. Summary boxplots of patient's hearing level in treated ear, by PTA frequency (0.25, 0.5, 1, 2, 3, 4, 6, 8, 12.5, 16 Hz), and by time point (baseline, 6 weeks and 12 weeks).

D2. Summary boxplots of patient's hearing level in untreated ear, similar as in D1.

D3. Summary boxplots of difference in patient's hearing level between treated and untreated ear, similar as in D1.

D4. Summary bar graphs of difference in changes from baseline to 6 and 12 weeks, between treated and untreated ear, by PTA frequency.

E. Similar plots as produced in D but stratified by patients with mild and moderate SNHL. A patient is classified as mild SNHL if (5-freq-PTA_{right} ≤ 40 dBHL and 5-freq-PTA_{left} ≤ 40 dBHL), while a patient is classified as moderate SNHL if either 5-freq-PTA_{right} or 5-freq-PTA_{left} is greater than 40 dBHL, where 5-freq-PTA is the average across five frequencies (0.5, 1, 2, 4 and 8 kHz) for each right and left ear, respectively.

F. Summary of responses (change from baseline ≤-10)

F1. Summary of the total number of responses across all participants in the treated ear will be produced per time point (6 and 12 weeks). These summaries will be generated in total, by frequency, and by frequency domain (PTA high and PTA low frequencies).

F2. Summary of the total number of responses in the untreated ear, similar as in F1.

9.7.2 Primary Efficacy Analysis

9.7.2.1 PTA average

The general null and alternative hypotheses to test the treatment effect is:

H_0: Mean=0 (i.e. no statistical evidence of difference in hearing function before/post-treatment)
H_1: Mean≠0 (i.e. statistical difference in hearing function before/post-treatment)

The primary endpoint will be analysed through a mixed-effect model for repeated measures on the average change in hearing level (in dB) in the treated ear across three frequencies (2, 4, 8 kHz) from baseline to 12 weeks. The model will include baseline value and age of patient as fixed continuous factors, and time (two time points: 6 and 12 weeks) as the continuous factor of repeated measures. Subject will be included as random factor. Unstructured covariance matrix will be used. The average change, and its 95% CI, between baseline and post-treatment will be obtained using the coefficient estimate for the time factor.

The respective line plot for the mean change from baseline to post-treatment will be displayed.

9.7.2.2 PTA domain

Comparison of average change in hearing level in the treated ear between high (from 2, 4, 8 kHz) and low frequencies (from 0.25, 0.5, 1 kHz) from baseline to 12 weeks will be performed using a mixed-effect model for repeated measures. The model will include baseline value and age of patient as fixed continuous factors, the group variable (high/low) as fixed categorical factor, and time (two time points: 6 and 12 weeks) as the continuous factor of repeated measures. Subject will be included as random factor. The difference, and its 95% CI, of average change from baseline to post-treatment between high and low group will be obtained from the coefficient estimate for the group factor.

The above primary efficacy analyses will also be performed using the per-protocol population to support the findings from the mITT population.

9.7.3 Secondary Analyses

The secondary endpoints will be summarised by time point (baseline, 6 weeks and 12 weeks), and by frequency when appropriate, along with the absolute change from baseline for continuous variables, and shift tables for categorical variables.

A mixed-effect model for repeated measures will be used (where appropriate) to assess the treatment effect, separately per frequency (where it applies), on continuous secondary endpoints. The model will include baseline value and age of patient as fixed continuous factor, and time (two time points: 6 weeks, and 12 weeks) as the continuous factor of repeated measures. Subject will be included as random factor. Unstructured covariance matrix will be used. The average change, and its 95% CI, between baseline and post-treatment will be obtained from the coefficient estimate for the time factor.

The McNemar’s Test for paired samples with two categories (or extended McNemar’s Test if more than two categories) will be used to assess the treatment effect on shifts of categories between baseline and post treatment of secondary endpoints.

9.7.3.1 Speech audiometry (Speech in noise)

Bisyllabic words in babble (WiB): Change from baseline to 6 and 12 weeks in SNR 50%.
Quick speech in noise (QuickSiN)**:** Change from baseline to 6 and 12 weeks in SNR as determined by QuickSiN methodology (SNR in dB).

Both endpoints will be evaluated using mixed-effect model for the treated ear as described in section 9.7.3. Summary tables for the number of responses, defined by a change of ≤ -2 dB, will be produced by treated/untreated ear. A summary of the difference in changes between treated and untreated ear will also be produced. These results will be presented per time point, at individual level (per patient) and group level (overall patients).

Graphical displays (line plots, boxplots and bar graphs) similar as those described in section 9.7.1.1. for PTA, will be produced to display the changes in SNR as appropriate.

9.7.3.2 Immitance (ART)

Change from baseline of the continuous ART endpoint in the treated ear will be evaluated using mixed-effect model as described in section 9.7.3.

Categorical ART endpoint comprises two categories (present ART, absent ART), where ART is classified as present, if ART>100 dB, and absent if ART≤100 dB, for all centres. Shift of categories in ART will be evaluated with the McNemar's Test.

Evaluation of ART endpoint will be considered at each frequency 0.5, 1, 2 and 4 kHz.

Graphical displays (line plots, boxplots and bar graphs), similar as described in section 9.7.1.1. for PTA, will be produced to display the changes in ART as appropriate.

9.7.3.3 Tympanometry: middle ear pressure, volume, and tympanogram type

The endpoints to evaluate are: continuous middle ear pressure, continuous middle ear volume, and categorical type of tympanogram with three categories (A, B, C).

Change from baseline to 12 weeks in a continuous endpoint in the treated ear will be analyzed using either a paired T-test or mixed-effect model as described in section 9.7.3, depending on the sample size.

Change from baseline to 12 weeks in the categorical endpoint will be evaluated with the McNemar's Test.

9.7.3.4 DPOAE (group level)

The following changes from baseline to 12 weeks will be evaluated:

- Change from baseline to 12 weeks in number of patients in the three categories (present and normal; present and abnormal; absent (cut off point SNR<6)) at the following frequencies: 500, 547, 596, 645, 703, 771, 840, 918, 996, 1094, 1299, 1416, 1543, 1680, 1836, 2002, 2178, 2383, 2598, 2832, 3086, 3359, 3672, 4004, 4365, 4756, 5186, 5654, 6172, 6729, 7334, 7998, 8721, 9512 in 65/55 and 70/70 dB SPL stimuli levels, for both the treated and untreated ear.

  These changes will be evaluated using the McNemar's Test, separately for treated and untreated ear. In addition, a summary table with the percentage of patients who changed categories from baseline to 12 weeks will be produced. The total percentage of changes will be compared between treated and untreated ear using the Z-Test for two proportions.

- Change from baseline to 12 weeks in SNRs (dB SNR) at the following frequencies: 500, 547, 596, 645, 703, 771, 840, 918, 996, 1094, 1299, 1416, 1543, 1680, 1836, 2002, 2178, 2383, 2598, 2832, 3086, 3359, 3672, 4004, 4365, 4756, 5186, 5654, 6172, 6729, 7334, 7998, 8721, 9512 in 65/55 and 70/70 dB SPL stimuli levels, for both the treated and untreated ear.

These changes will be evaluated following a mixed-effect model as described in section 9.7.3. Line plots displaying the individual patient's change at baseline, 6 weeks and 12 weeks will be produced.

- Change from baseline to 12 weeks in absolute threshold (dB) at the following frequencies: 500, 547, 596, 645, 703, 771, 840, 918, 996, 1094, 1299, 1416, 1543, 1680, 1836, 2002, 2178, 2383, 2598, 2832, 3086, 3359, 3672, 4004, 4365, 4756, 5186, 5654, 6172, 6729, 7334, 7998, 8721, 9512 in 65/55 and 70/70 dB SPL stimuli levels, for both the treated and untreated ear.

  These changes will be evaluated following a mixed-effect model as described in section 9.7.3. Similar evaluation will be performed for changes from baseline to 6 weeks.

Line plots displaying the patient's SNR (dB) against the frequencies, at baseline, 6 weeks and 12 weeks will be produced.

9.7.3.5 Threshold-Equalizing-Noise (TEN), (group level)

The endpoint to evaluate is the categorical TEN dead region with two categories (Yes, No).

Changes from baseline to post-treatment (6 and 12 weeks) between the two categories will be evaluated with the McNemar's Test.

9.7.3.6 Hearing Handicap Score

The endpoints to evaluate are outcome questionnaire continuous scores: total score, social score, and emotional score. Each score will be classified into three categories: no handicap, mild-moderate handicap, and significant handicap.

The change from baseline to 6 and 12 weeks in the categorical scores of the subjects will be evaluated with the McNemar's Test.

The change from baseline to 6 and 12 weeks in the total continuous score of the subjects will be evaluated with either a paired T-test or mixed-effect model, depending on sample size. Line plots (alternatively, bar graphs) comparing the individual patient's total score at baseline, 6 weeks and 12 weeks will be produced.

9.7.3.7 Dizziness Handicap Inventory (DHI)

The endpoints to evaluate are summary continuous scores: total score, quality of life score, and symptoms score. Other scores are symptom sub-scale scores: dizziness score, anxiety score, and motion-provoked dizziness score.

Change from baseline to 12 weeks in each of the scores of the subjects will be evaluated with either paired T-test or mixed-effect model, depending on sample size.

Line plots (alternatively, bar graphs) comparing the individual patient's total score at baseline and 12 weeks will be produced.

9.7.3.8 Tinnitus Functional Index (TFI)

The endpoints to evaluate are continuous total TFI score, and categorical TFI score with three categories (Low, Medium, Severe).

Changes from baseline to 12 weeks in the categorical TFI score will be evaluated with a McNemar's Test. Change from baseline to 12 weeks in total TFI score will be evaluated with either a paired T-test or mixed-effect model, depending on sample size.

Graphical display as described in section 9.7.3.7.

9.7.3.9 Hearing Aid Outcome Questionnaire

The endpoints to evaluate are: continuous total Hearing Handicap Score (HHS) score, and categorical HHS score with three categories (Low, Medium, Severe).

Change from baseline to 12 weeks in the categorical HHS score will be evaluated with a McNemar's Test. Change in the continuous HHS score will be evaluated with either a paired T-test or mixed-effect model, depending on sample size.

Graphical display as described in section 9.7.3.7.

9.7.3.10 Eye Movements

The endpoint to evaluate is the eye movement with two categories (Normal, Abnormal). Change from baseline to 6 and 12 weeks in the eye movement category will be evaluated with McNemar's Test, respectively.

9.7.3.11 Modified Romberg

The endpoint to evaluate is the modified Romberg with two categories (Positive, Negative; with Positive being associated with loss of balance). Change from baseline to 6 and 12 weeks in the modified Romberg category will be evaluated with McNemar's Test, respectively.

9.7.3.12 Unterberger

The endpoint to evaluate is the Unterberger with two categories (Positive, Negative; with Positive being associated with loss of balance). Change from baseline to 6 and 12 weeks in the unterberger category will be evaluated with the McNemar's Test, respectively.

9.7.3.13 Head Thrust Test

The endpoint to evaluate is the head thrust test with two categories (Normal vestibulo-ocular reflex gain, Reduced vestibulo-ocular reflex gain). Change from baseline to 6 and 12 weeks in the categories of vestibulo-ocular reflex gain will be evaluated with the McNemar's Test, respectively.

9.7.3.14 VNG Air Calorics

The endpoint to evaluate is the VNG Air Calorics with three categories (Hyperactive, Normal, Hypoactive; with both Hyperactive and Hypoactive being associated with problems in balance).

Change from baseline to 12 weeks in the above categories will be evaluated with the McNemar's Test.

9.7.3.15 Facial nerve function

The endpoint to evaluate is the facial nerve function with two categories (Normal, Abnormal). Change from baseline to 6 and 12 weeks in the above categories will be evaluated with the McNemar's Test, respectively.

9.7.3.16 Taste Assessment

The endpoint to evaluate is the taste with three categories (Loss of taste, Altered taste, No change). Change from baseline to 6 and 12 weeks in the taste categories will be evaluated with the McNemar's Test, respectively.

9.7.3.17 Tinnitus Assessment

The endpoint to evaluate is the tinnitus with three categories (No change, Louder, Less loud). Change from baseline to 6 and 12 weeks in the tinnitus categories will be evaluated with the McNemar's Test, respectively.

9.7.4 Exploratory Efficacy Analyses

9.7.4.1 Follow-up of endpoints at 6 and 12 months

As optional follow-up visits at month 6 and 12 will be available, similar analyses as described in sections 9.7.1 and 9.7.2.1 for the primary endpoint, and section 9.7.3.1 for the secondary endpoint will be performed for publication purposes. For this analysis, the time to follow-up will be considered as a categorical repeated measure variable, and treatment status of the ear will be added in the model. The interaction of time with treatment will also be accounted for.

The objective of this analysis is to evaluate the efficacy of treatment at time of follow-up visit, with respect to the baseline. Moreover, evaluation of changes compared to the untreated ear will be conducted.

The analysis will be performed using a mixed-effect model for repeated measures of the endpoint at each follow-up visit. The model will include a fixed, categorical effect of treatment (treated/untreated), categorical repeated measures of time visits (e.g. 6 weeks, 12 weeks, 6 months, and 12 months, if available), the interaction of treatment and time. The model will consider adjustment for fixed, continuous covariates: baseline and age of patient. Subject will be included as random factor. Unstructured covariance matrix will be used.

The adjusted estimated effect of treatment and its 95% CI will be obtained using the coefficient estimates of the factors from the mixed-effect model. The respective line plots for the estimated adjusted means, per treatment category, will be displayed.

Other exploratory analyses will not be considered in this version and will be added later if necessary.

9.7.5 Subgroup Analyses

Endpoints for pure-tone audiometry (PTA) and for speech audiometry (Words in babble) will be analysed per site, in addition to the pooled analysis.

Endpoints for speech audiometry (QuickSiN) will be analysed only per site, as a pooled analysis is not appropriate. See section 7.7.

Descriptive subgroup analyses by patients with mild and moderate hearing loss level will be performed for PTA endpoint. See section 9.7.1.1, E.

9.8 Safety Analysis

The safety data will be summarised descriptively overall using the safety analysis set. The following items will be considered: Hearing protection (statement only), Physical Exam including neuro (listed only), Otomicroscopy (listed as physical exam), Hearing Aid use (statement only), Contraception (statement only), Ototoxic drugs (statement only).

9.8.1 Physical Examination

Physical examination results (normal, abnormal – not clinically significant, abnormal – clinically significant) and description of abnormality will be listed by visit. Physical examination results will be summarised by organ system and visit. Shift in examination results categories will be presented.

9.8.2 Vital Signs

For each recorded vital sign (heart rate, blood pressure, respiration rate, and body temperature), all available measurements, and changes from baseline to all post-treatment visits will be listed. Vital sign measurements, and changes from baseline to post-treatment visits, will be summarised by visit.

9.8.3 ECG

ECG assessments will be listed with date-time, ECG parameters, interpretation, and abnormalities (if any). ECG interpretation (normal, abnormal – not clinically significant, abnormal – clinically significant) will be summarised by visit.

9.8.4 Laboratory measurements

For all recorded laboratory parameters (haematology, biochemistry, urinalysis, pregnancy test), the number and proportion of measurements below the lower limit of quantification will be tabulated.

For all recorded laboratory parameters, all available measurements will be listed. Changes from baseline to all post-treatment visits will be listed for quantitative parameters. Laboratory parameter measurements will be summarised for all visits. Changes from baseline to post-treatment visits will be summarised for quantitative parameters. For laboratory parameters

where normal ranges are available, shift from baseline will be tabulated for all post-treatment visits.

9.8.5 Adverse Events

An overall listing of AEs by patient with information on reported term, system organ class, preferred term, start and end date-time, actions taken, severity, expectedness, seriousness, relatedness to study product and procedure, and outcome will be produced. Listing of AEs within 24 hours after injection will be included. Analogous listings will be produced containing only deaths, serious adverse events (SAEs), suspected unexpected serious adverse reactions (SUSARs) and AEs leading to study discontinuation.

The total number of AEs per patient, as well as the proportion of patients with at least one AE will be summarised by each of system organ class, preferred term, relatedness to study product and procedure, and severity. Analogous summaries will be produced for deaths, SUSARs, SAEs, and AEs leading to study discontinuation.

10 Reporting Conventions

In general, descriptive statistics will be presented with the following number of decimal places:

- Absolute frequencies, number of missing values: no decimal places
- Relative frequencies [in %]: rounded to one decimal place
- Minimum, mean, 25%-quantile, median, 75%-quantile, maximum: the same number of decimal places as the original data
- P-values will be reported to three (3) decimal places; p-values less than 0.001 will be reported as “<0.001”.
- All other statistics will be reported to one (1) decimal place greater than the original data.

11 Technical Details

The analysis will be carried out according to standard operating procedure (SOP) SOP001_PROGRAMMING [3]. The statistical analysis will be performed using SAS® 9.4 or higher.

SAS® programming will be performed according to Staburo GmbH standards as defined in SOP001_PROGRAMMING [3] and related work instructions. Special attention will be paid to planning and performance of quality control measures as documented in the quality control plan for the analysis of this study (see also SOP002_PROGRAM_QC [4]).

12 Summary of Changes to the Protocol

- Endpoints for speech in noise will be analysed separately per site, no pooled analysis will be performed, as justified in section 7.7.
- Descriptive analyses at individual patient level and per frequency (where applicable) have been added, based on insights from REGAIN – part A.
- Age of patient has been added as covariate in the mixed-effect models.
- Comparison between PTA domain (lower frequency average and higher frequency average) has been added.
- Given the paired sample feature for the analysis of switching categories between time-points, the Chi-square Test has been replaced by the McNemar's Test for the analysis of shift categories from baseline to post-treatment (6 and 12 weeks) in secondary endpoints.

13 Contents of Tables, Figures and Listings

Based on this SAP, an excel file is developed which covers all tables, figures and listings to be included in the clinical study report. This document is appended to this SAP and will be available upon request. In accordance with ICH E3 [1] the statistical output is organised in Tables and Figures as follows:

14 TABLES, FIGURES AND GRAPHS REFERRED TO BUT NOT INCLUDED IN THE TEXT

- 14.1 Demographic data and baseline characteristics
- 14.2 Efficacy data
- 14.3 Safety data

16 APPENDICES

- 16.2 PATIENT DATA LISTINGS

14 References

[1] International Conference on Harmonisation. Note for Guidance on Structure and Content of Clinical Study Reports (ICH E3). CPMP/ICH/137/95, 1996.

[2] International Conference on Harmonisation. Note for Guidance on Statistical Principles for Clinical Trials (ICH E9). CPMP/ICH/363/96, 1998.

[3] SOP001_PROGRAMMING, "Standard Operating Procedure for Statistical Programming", current version

[4] SOP002_PROGRAM_QC, "Standard Operating Procedure for Quality Control of programs", current version
