# Supplementary material for: A phase I/IIa safety and efficacy trial of intratympanic gamma-secretase inhibitor as a regenerative drug treatment for sensorineural hearing loss
Source: Nat Commun. 2024 Mar 1;15:1896. doi: 10.1038/s41467-024-45784-0 (PMC10907343; doi:10.1038/s41467-024-45784-0)
Supplement: Supplementary file 7 — Reporting Summary [file 41467_2024_45784_MOESM7_ESM.pdf]

nature portfolio

Corresponding author(s): Stephan Wolpert

Last updated by author(s): Nov 21, 2023

Reporting Summary

Nature Portfolio wishes to improve the reproducibility of the work that we publish. This form provides structure for consistency and transparency in reporting. For further information on Nature Portfolio policies, see our Editorial Policies and the Editorial Policy Checklist.

Statistics

For all statistical analyses, confirm that the following items are present in the figure legend, table legend, main text, or Methods section.

n/a	Confirmed	
☐	☒	The exact sample size (*n*) for each experimental group/condition, given as a discrete number and unit of measurement
☐	☒	A statement on whether measurements were taken from distinct samples or whether the same sample was measured repeatedly
☐	☒	The statistical test(s) used AND whether they are one- or two-sided<br>*Only common tests should be described solely by name; describe more complex techniques in the Methods section.*
☐	☒	A description of all covariates tested
☐	☒	A description of any assumptions or corrections, such as tests of normality and adjustment for multiple comparisons
☐	☒	A full description of the statistical parameters including central tendency (e.g. means) or other basic estimates (e.g. regression coefficient) AND variation (e.g. standard deviation) or associated estimates of uncertainty (e.g. confidence intervals)
☐	☒	For null hypothesis testing, the test statistic (e.g. *F*, *t*, *r*) with confidence intervals, effect sizes, degrees of freedom and *P* value noted<br>*Give P values as exact values whenever suitable.*
☐	☒	For Bayesian analysis, information on the choice of priors and Markov chain Monte Carlo settings
☐	☒	For hierarchical and complex designs, identification of the appropriate level for tests and full reporting of outcomes
☐	☒	Estimates of effect sizes (e.g. Cohen's *d*, Pearson's *r*), indicating how they were calculated

Our web collection on statistics for biologists contains articles on many of the points above.

Software and code

Policy information about availability of computer code

Data collection	No software was used
Data analysis	Analyses were performed with SAS (v9.4), SPSS (v26), and R (v3.3.1)

For manuscripts utilizing custom algorithms or software that are central to the research but not yet described in published literature, software must be made available to editors and reviewers. We strongly encourage code deposition in a community repository (e.g. GitHub). See the Nature Portfolio guidelines for submitting code & software for further information.

Data

Policy information about availability of data

All manuscripts must include a data availability statement. This statement should provide the following information, where applicable:

- Accession codes, unique identifiers, or web links for publicly available datasets
- A description of any restrictions on data availability
- For clinical datasets or third party data, please ensure that the statement adheres to our policy

The final study protocol and statistical analysis plan are provided as Supplementary Note. Pure tone audiometry data is given in the supplemetary appendix, additional data that support the findings of this study can be made available from the REGAIN consortium upon reasonable request, responses will be sent within 2 weeks. Restriction apply to the availability of these data due to planned regulatory submission.

Research involving human participants, their data, or biological material

Policy information about studies with human participants or human data. See also policy information about sex, gender (identity/presentation), and sexual orientation and race, ethnicity and racism.

Reporting on sex and gender	Gender of the participants was determined on self-report.
Reporting on race, ethnicity, or other socially relevant groupings	Race, ethnicity are not reoprted in the manuscript.<br>Years of schooling and education are listed in a table, but were not analyzed further.
Population characteristics	Adult patients with a primary complaint of hearing loss, their history suggesting an age-related, noise-induced or unknown cause, and diagnosed at the trial screening visit with bilateral symmetrical mild to moderate SNHL (mean pure-tone hearing threshold 25 to 60 decibel hearing level (dB HL) across frequencies 0.5, 1, 2, 4 and 8 kHz) were eligible for the Phase I/II trial. Patients over the age of 80, with a history of fluctuating hearing loss or of chronic or ongoing middle ear disease were excluded.
Recruitment	Study participants were recruited independently via the three trial centers in London, Athens and Tübingen. Recruitment took place via the outpatient clinic, referring colleagues and via the media.
Ethics oversight	The trial protocol was approved by national regulatory authorities, medical ethical committees and local R&D authorities in London (London, Central REC Committee, REC Number: 17/LO/0632), Athens (Hellenic Republic Ministry of Heath National Ethics Committee - Reg. No.: 83052/2018) and Tübingen (Ethik-Kommission der Medizinischen Fakultät und am Universitätsklinikum Tübingen, Proj. No.: 592/2018AMG1).

Note that full information on the approval of the study protocol must also be provided in the manuscript.

Field-specific reporting

Please select the one below that is the best fit for your research. If you are not sure, read the appropriate sections before making your selection.

☒ Life sciences ☐ Behavioural & social sciences ☐ Ecological, evolutionary & environmental sciences

For a reference copy of the document with all sections, see nature.com/documents/nr-reporting-summary-flat.pdf

Life sciences study design

All studies must disclose on these points even when the disclosure is negative.

Sample size	For Phase IIa we set a recruitment target of 40 patients, based on 87% power to detect a 10 dB(HL) change (standard deviation 20 dB(HL)) corresponding to an effect size of 0.50.
Data exclusions	No data was excluded.
Replication	In order to guarantee repeatability, (audiometric) measurements were carried out according to a defined protocol (see METHODS) and by trained personnel.
Randomization	Since there was only one (treatment) group in the study, no randomization was possible.
Blinding	Since there was only one (treatment) group in the study, also no blinding was possible.

Reporting for specific materials, systems and methods

We require information from authors about some types of materials, experimental systems and methods used in many studies. Here, indicate whether each material, system or method listed is relevant to your study. If you are not sure if a list item applies to your research, read the appropriate section before selecting a response.

Materials & experimental systems

n/a	Involved in the study
☒	☐ Antibodies
☒	☐ Eukaryotic cell lines
☒	☐ Palaeontology and archaeology
☒	☐ Animals and other organisms
☐	☒ Clinical data
☒	☐ Dual use research of concern
☒	☐ Plants

Methods

n/a	Involved in the study
☒	☐ ChIP-seq
☒	☐ Flow cytometry
☒	☐ MRI-based neuroimaging

Clinical data

Policy information about clinical studies

All manuscripts should comply with the ICMJE guidelines for publication of clinical research and a completed CONSORT checklist must be included with all submissions.

Clinical trial registration

ISRCTN59733689, EudraCT Number: 2016-004544-10

Study protocol

The final study protocol and statistical analysis plan are provided as Supplementary Note.

Data collection

Phase 1: first patient enrolled – 24 January 2018, last patient last dose – 17 October 2018 (
Phase 2a: first patient enrolled - January 30, 2019, last patient last dose - August 05, 2019,
last assessment in August 2020.
Phase 1: Royal National Throat Nose and Ear Hospital, University College London Hospital Trust, London, UK
Phase 2a:
- Royal National Throat Nose and Ear Hospital, University College London Hospital Trust, London, UK
- 1st Department of Otolaryngology, Hippocration Hospital Athens, National & Kapodistrian University of Athens, Greece
- Department of Otolaryngology, Head and Neck Surgery, University of Tübingen, Germany

Outcomes

The primary outcome of Phase I was safety and tolerability in terms of occurrence and severity of treatment and procedure related local and systemic Adverse Events (AEs). Secondary outcome was the optimal dose of LY3056480 for Phase IIa. Local safety outcomes included changes in hearing in the treated ear, tinnitus, balance and facial nerve function.

The Phase IIa primary efficacy outcome was change in the mean pure-tone air conduction threshold (in dB HL) across 2, 4 and 8 kHz in the treated ear from baseline to 3 months with a mean improvement of ≥10 dB(HL) considered clinically significant. Secondary efficacy outcomes included change from baseline at 3, 6 and 12 months, in the treated and untreated ear, for: 1) mean pure-tone air conduction threshold across 2, 4 and 8 kHz (dB HL); 2) pure-tone air conduction thresholds at individual frequencies (dB HL); 3) dB SNR of 50% speech reception threshold (SRT); 4) distortion product otoacoustic emissions (DPOAE) amplitude and SNR, for both low-level (65/55 dB SPL) and high-level (70/70 dB SPL) tone primaries; 5) Hearing Handicap Inventory for Adults/Elderly (HHIA/E) score.

For Phase IIa we set a recruitment target of 40 patients, based on 87% power to detect a 10 dB(HL) change (standard deviation 20 dB(HL)) corresponding to an effect size of 0.50.

Plants

Seed stocks

n.a.

Novel plant genotypes

n.a.

Authentication

n.a.
